# Identification of RAD51–BRCA2 Inhibitors Using *N*-Acylhydrazone-Based Dynamic Combinatorial Chemistry

**DOI:** 10.1021/acsmedchemlett.2c00063

**Published:** 2022-07-28

**Authors:** Greta Bagnolini, Beatrice Balboni, Fabrizio Schipani, Dario Gioia, Marina Veronesi, Francesca De Franco, Cansu Kaya, Ravindra P. Jumde, Jose Antonio Ortega, Stefania Girotto, Anna K. H. Hirsch, Marinella Roberti, Andrea Cavalli

**Affiliations:** † Computational & Chemical Biology (CCB), Istituto Italiano di Tecnologia (IIT), 16163 Genova, Italy; ‡ Department of Pharmacy and Biotechnology (FaBiT), University of Bologna, 40126 Bologna, Italy; § Helmholtz Centre for Infection Research (HZI), Helmholtz Institute for Pharmaceutical Research Saarland (HIPS), 66123 Saarbrücken, Germany; ∥ Structural Biophysics and Translational Pharmacology, Istituto Italiano di Tecnologia (IIT), 16163 Genova, Italy; ⊥ D3-PharmaChemistry, Istituto Italiano di Tecnologia (IIT), 16163 Genova, Italy; # TES Pharma Srl, I-06073 Corciano, Perugia Italy; 7 Department of Pharmacy, Saarland University, 66123 Saarbrücken, Germany

**Keywords:** RAD51, DNA repair, ptDCC, hit-identification strategy, protein−protein interaction inhibitor

## Abstract

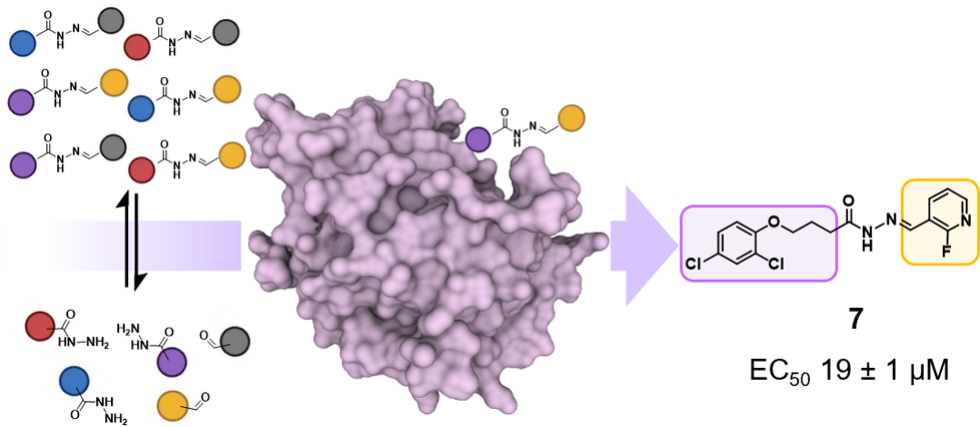

RAD51 is an ATP-dependent recombinase, recruited by BRCA2 to mediate DNA double-strand breaks repair through homologous recombination and represents an attractive cancer drug target. Herein, we applied for the first-time protein-templated dynamic combinatorial chemistry on RAD51 as a hit identification strategy. Upon design of *N*-acylhydrazone-based dynamic combinatorial libraries, RAD51 showed a clear templating effect, amplifying 19 *N*-acylhydrazones. Screening against the RAD51–BRCA2 protein–protein interaction via ELISA assay afforded 10 inhibitors in the micromolar range. Further ^19^F NMR experiments revealed that **7** could bind RAD51 and be displaced by BRC4, suggesting an interaction in the same binding pocket of BRCA2. These results proved not only that ptDCC could be successfully applied on full-length oligomeric RAD51, but also that it could address the need of alternative strategies toward the identification of small-molecule PPI inhibitors.

RAD51 is a central protein in homologous recombination (HR), a high-fidelity repair of DNA double-strand breaks (DSBs). HR dysregulation contributes to cancer development and progression. RAD51 overexpression and elevated RAD51-mediated HR rate are both observed in a variety of cancers^1,2^ and positively correlate with drug resistance.^3^ Thus, RAD51 represents an attractive target for anticancer treatments. Indeed, reducing RAD51 activity has been proposed as strategy to sensitize cancer cells and overcome resistance to existing DNA-damaging therapies. In recent years, modulators of RAD51 activity or expression have been developed and comprehensively reviewed.^4−6^ Importantly, in the context of synthetic lethality (SL), clinical trial NCT03997968 is now studying RAD51 inhibitor CYT-0851 to treat malignancies with overexpression of DNA-damaging cytidine deaminases.^7^

RAD51 is localized in the cytoplasm in self-assembled filaments and recruited at the site of DNA DSBs by BRCA2 through a protein–protein interaction (PPI). Once at the DSB, BRCA2 assists RAD51 nucleofilaments formation on DNA. Thus, RAD51–BRCA2 PPI represents a critical event for DSB repair. In BRCA defective cells, poly(ADP-ribose) polymerase inhibitors (PARPis) trigger selective cancer cell death through SL. Nowadays, the clinical example is the use of the PARPi olaparib for ovarian, breast, and pancreatic tumors with BRCA mutations.^8−10^

In this context, to extend the use of PARPi, the interaction surface between RAD51 and BRCA2 has been proposed as target in BRCA2-proficent tumors, and several small-molecule disruptors have been identified.^11−13^ Recently, we reported a series of dihydroquinolone pyrazoline-based small-molecules able to disrupt the RAD51–BRCA2 interaction and inhibit HR in BxPC-3 cell line. Ultimately, the synergy with olaparib triggered the dubbed “fully small-molecule-induced SL”. Despite the compounds’ promising results, the low solubility and bioavailability prevented their application in *in vivo* cancer models.^14^

In continuation with our work, we applied for the first-time protein-templated dynamic combinatorial chemistry (ptDCC) as an elegant alternative approach to identify new structurally diverse RAD51 ligands, potentially able to inhibit RAD51–BRCA2 PPI.

ptDCC has evolved as innovative and an efficient hit-identification tool for proteins of therapeutic interest.^15−18^ Its potential relies on the possibility for the target protein to select in situ its own high affinity ligands. In ptDCC, compounds are generated through reversible and biocompatible chemical reactions. The continuous interconversion of building blocks and products forms the dynamic combinatorial libraries (DCLs), regulated by a thermodynamic equilibrium. In presence of the protein as template, a molecular recognition process takes place through noncovalent interactions, named as the templating effect. Indeed, if one or more library components bind to the protein, the equilibrium will be shifted according to Le Châtelier’s principle, amplifying concentrations of high-affinity binders, leaving behind weak or nonbinding components (Figure 1).

**Figure 1:**
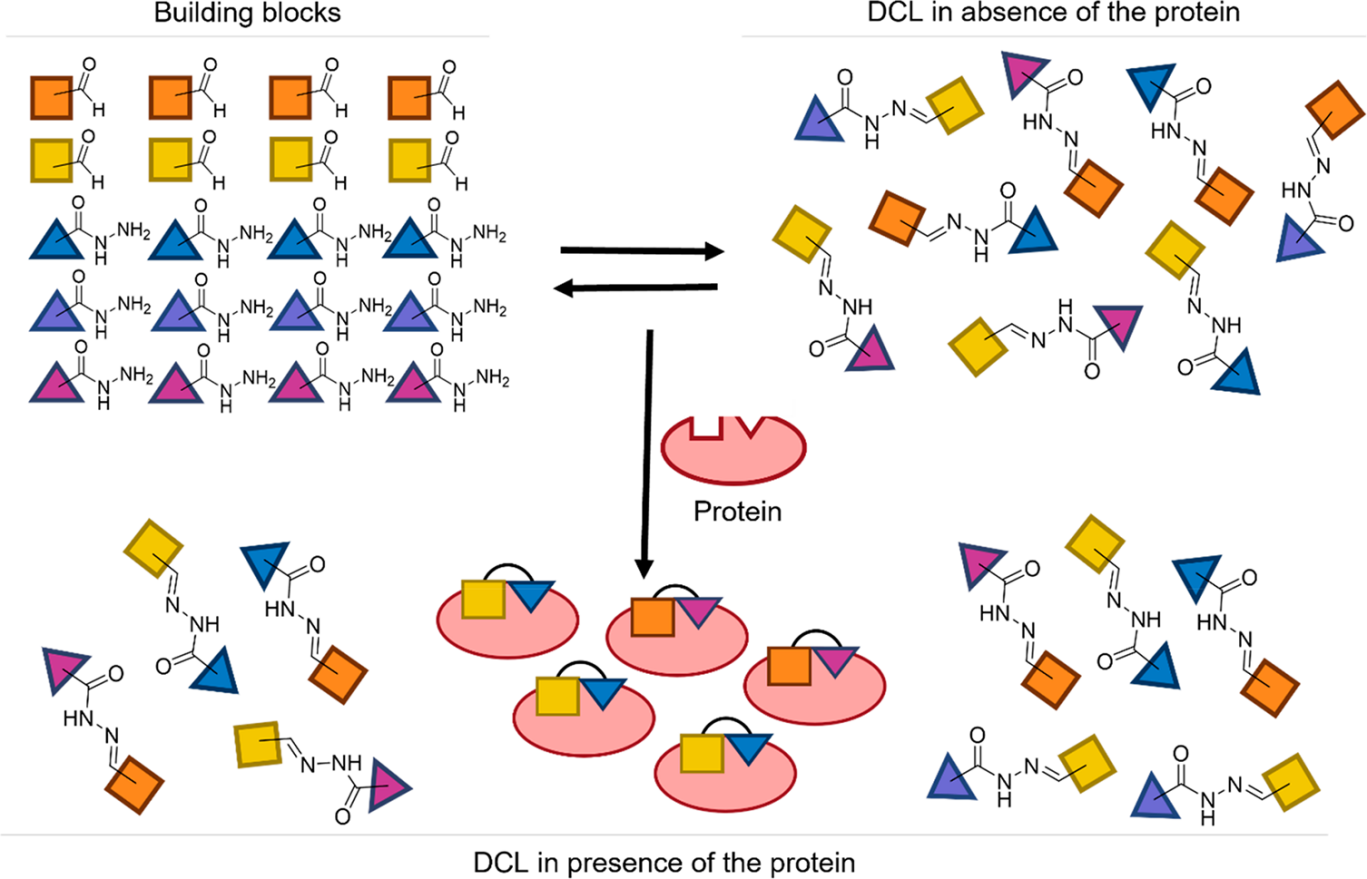
Schematic illustration of *N*-acylhydrazone-based ptDCC.

Ultimately, this allows the identification of the best protein binders, reducing synthesis, purification, and characterization efforts. So far, ptDCC has been employed on a wide range of proteins at various stages of medicinal chemistry.^19,20^ Notably, ptDCC could represent a useful alternative to explore targets featuring poorly defined pockets, not easily addressed by structure-based drug discovery (SBDD). Here, through ptDCC, we identified new RAD51 binders based on the *N*-acylhydrazone scaffold (**1**–**19**). Additionally, as suggested by biochemical ELISA and ^19^F-NMR assays, they could act as RAD51–BRCA2 inhibitors.

Among the arsenal of ptDCC chemical reactions,^15−17^ we chose the *N*-acylhydrazones (NAHs) formation, occurring between *N*-acylhydrazides and aldehydes. The reaction is reversible in acidic media, the equilibrium can be easily freezed by increasing pH to allow the analysis of the DCL composition. As an advantage, *N*-acylhydrazone linkage property to resemble an amide functionality promises to contribute to protein binding, as hydrogen-bond acceptor and donor.^18,21^

Here, RAD51 was used as wild-type full-length oligomeric form to better resemble its physiological cellular state.^22^ Protein preparation was achieved according to previously reported protocol.^14^

As prerequisite, we checked RAD51 stability in different buffers and pH values by measuring its melting temperature (*T*_m_) *via* thermal-shift assay/differential scanning fluorimetry (TSA/DSF) (Supporting Information (SI), Figure S1). From the screening, we selected a RAD51 storage buffer (SI, section 1) as it guaranteed protein stability up to 48 h (SI, Figure S1). Herein, we employed RAD51 in substoichiometric amount to prevent protein precipitation while still ensuring the protein-templating effect.^18^ Notably, as the chosen pH value was higher than 6.0, the use of aniline as nucleophilic catalyst was strictly required to keep the reversibility of *N*-acylhydrazones formation.^16,18^

The first DCL (DCL1) probed the applicability of RAD51 as DCC templating protein. To broaden the structural diversity of potential RAD51 binders, we designed DCL1 made of three aldehydes (**A1**–**A3**) and eight *N*-acylhydrazides (**H1**–**H8**), including structurally heterogeneous substituents, with different flexibility and conjugation degree, size, with electrodonor and withdrawing substituents, and H-bond donors and acceptors groups (Scheme 1). Specifically, DCL1 combined building blocks (BBs) with substituted phenyl rings (**A1**, **H2**, **H4**), mono- (**H1**) and biheterocycles (**A2**, **H5**, **H6**, **H7**), and partially (**A3**, **H3**) or fully aliphatic (**H8**) moieties (Scheme 1). As standard protocol, DCC experiments were set up as two reactions in parallel: (i) a protein-templated reaction with BBs reacting in the presence of RAD51, and (ii) a “blank” reaction in which only BBs were present (Scheme 1; SI, Table S1).

**Scheme 1:**
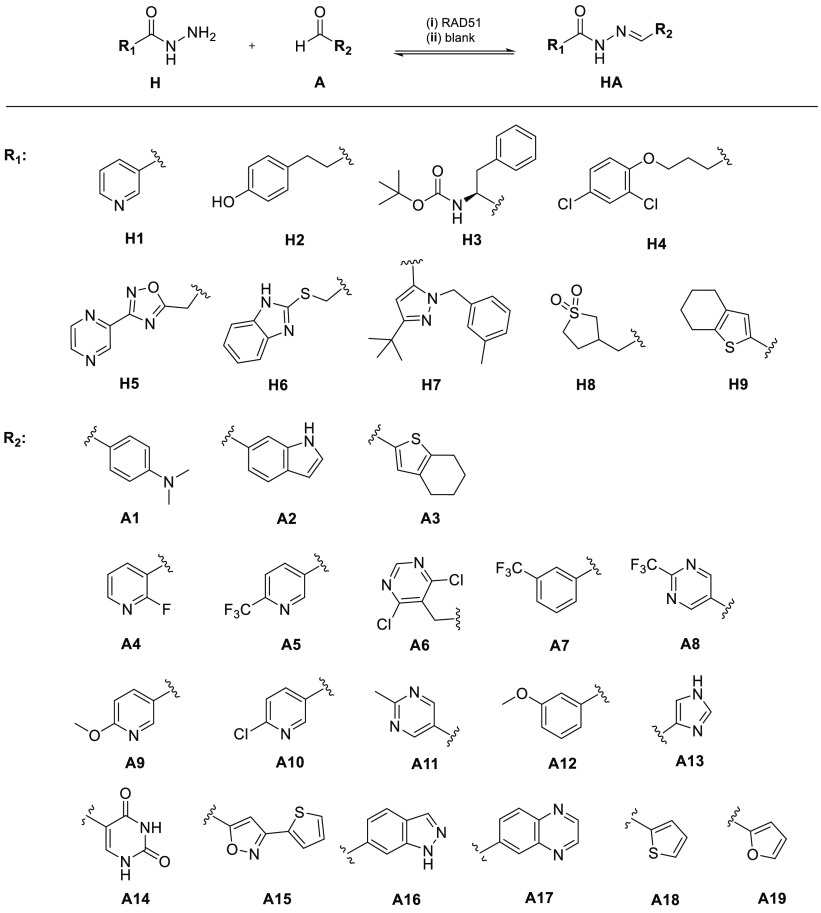
Dynamic Combinatorial Libraries (DCLs) in RAD51-Templated (i) and Blank Reactions (ii) Reagents and conditions: DCL1: (i) and (ii) *N*-acylhydrazides **H1**–**8**, aldehyde **A1**–**3**; (i) RAD51 (17.2 μM); DCL2: (i) and (ii) *N*-acylhydrazides **H3**–**4**, aldehyde **A4**–**13**; (i) RAD51 (17.2 μM); DCL3: (i) and (ii) *N*-acylhydrazides **H3**–**4**, **9**, aldehyde **A14**–**19**; (i) RAD51 (17.2 μM).

As shown by LC-MS, DCL1 blank reaction reached the equilibrium after 24 h (SI, Figure S2, Table S2). At 48 h, the presence of RAD51 significantly increased the peak areas of six *N*-acylhydrazones (**1**–**6**, Figure 2; SI, Figure S3, Table S3). Amplification factors are reported in Table 1 (SI, Figures S4,S5, Tables S4,S5). RAD51 successfully induced the amplification of specific library components, rising as a suitable ptDCC template. Herein, unassigned peaks could be ascribed to unidentified side products or unreacted *N*-acylhydrazides (Figure 2). Despite the clear amplification effect, we could not help noticing abnormalities in the chromatogram baseline, probably caused by buffer components interference. This necessarily required a buffer optimization for further experiments.

**Figure 2:**
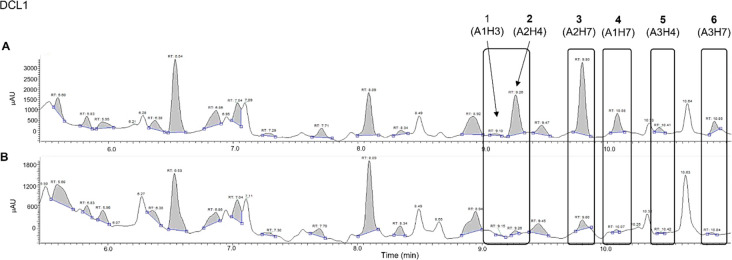
DCL1 UV chromatograms (290 nm) in protein-templated (A) and blank (B) reactions, 48 h.

**Table 1:**
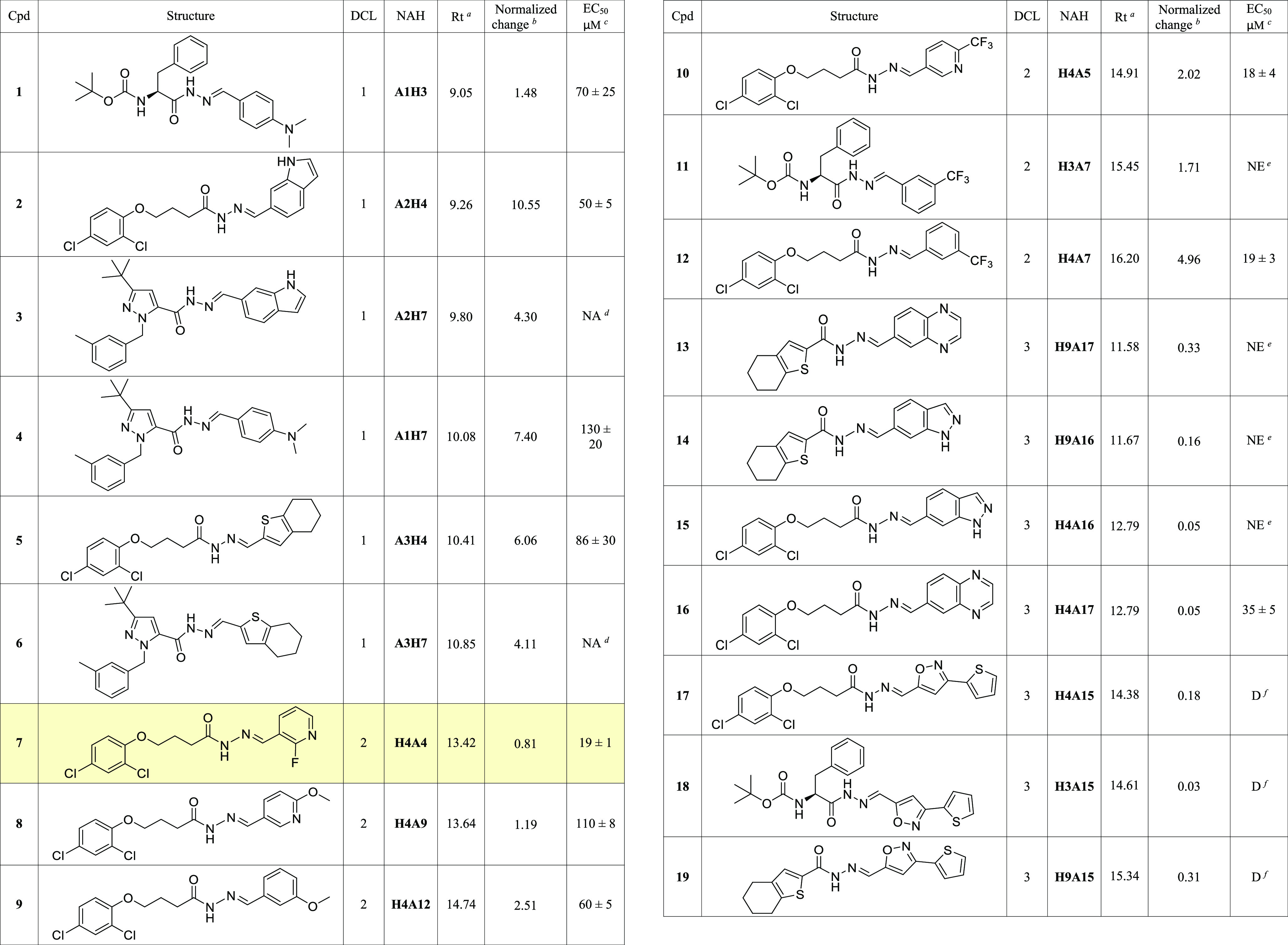
Structures, Rt, Amplification Scores Reported As Normalized Change and EC_50_ from ELISA Assay of NAHs **1**–**19**

a Retention time (min).

b Normalized change values (DCL1 48 h, DCL2 10 h, DCL3 8 h).

c Determined by biochemical ELISA assay.

d NA: not active.

e NE: not evaluable, due to poor solubility.

f D: discarded.

The reaction between the appropriate *N*-acylhydrazides and aldehydes in stoichiometric amount achieved the corresponding **1**–**6**, as mixture of *E*/*Z* amide rotamers of the *E* imine, in good yields (Scheme 2; SI, section 3).

**Scheme 2:**
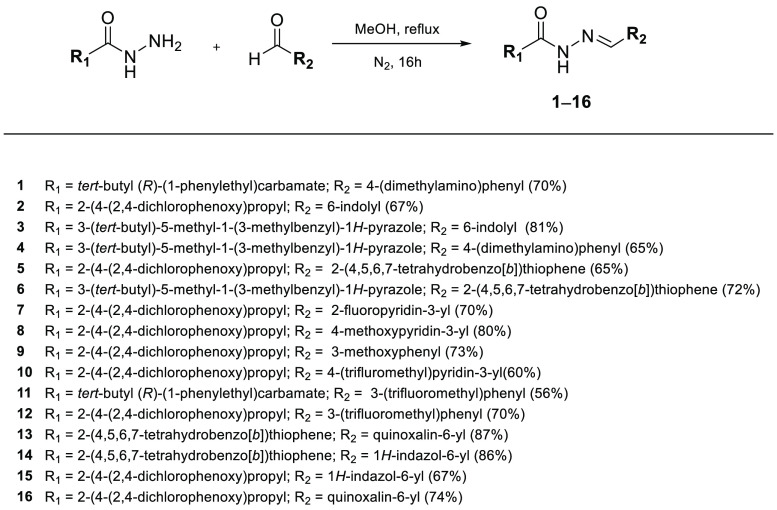
Synthesis of **1**–**16**

According to ptDCC experimental design, in principle, any available pocket or surface portion of RAD51 oligomer could act as a template. Consequently, as our interest regarded the identification of RAD51–BRCA2 PPI inhibitors, we screened **1**–**6** in a competitive biochemical ELISA assay, previously employed by our group.^11,12,14^ This assay allows the selection of compounds that inhibit the interaction between RAD51 and BRC4, the BRCA2 amino acid sequence displaying the highest affinity for RAD51.^23^ According to the time reported for the hydrolysis of NAH linker at physiological pH,^24^ NAHs were chemically stable to be tested in this assay. Among the tested NAHs, we obtained EC_50_ values in the double-digit micromolar range, with **1**, **2**, and **5** bearing flexible chains decorated with a single aromatic ring on the *N*-acylhydrazide counterpart (**H3**, **H4**) (Table 1).

These preliminary results showed that three NAHs could potentially act as PPI inhibitors, opening the way to ptDCC as potential tool toward RAD51–BRCA2 PPI inhibitors. Here, we argued that using structural motifs common to the active **1**, **2**, and **5** could further boost our chances of identifying more potent hits. Thus, we designed two tailored libraries, DCL2 (**H3**–**H4**, **A4**–**A13**) and DCL3 (**H3**–**H4**, **H9**, **A14**–**A19**) (Scheme 1), trying to tune the structure of emerged **1**, **2**, and **5**. Notably, for DCL2 we selected aromatic aldehydes with high structural similarities, including homo- and heterocyclic, 6- and 5-membered rings (Scheme 1). Whereas in DCL3 we combined more heterogeneous moieties such as rigid planar conjugated systems, biheterocyclic groups, and 5-membered heteroaromatic rings (Scheme 1). Importantly, aware of the interference issues seen in DCL1, here we used an optimized buffer composition (SI, section 2).

Following the standard protocol, we set up ptDCC experiments as two parallel reactions, (i) a protein-templated reaction, and (ii) a “blank” reaction (Scheme 1; SI, Table S6–S7). DCL2 and DCL3 blank reactions showed as reaching the equilibrium between 8–10 h and 6–8 h, respectively (SI, Figures S6–S7, Table S8–S9). In this time range, we observed RAD51-templating effect resulting in the amplification of **7**–**12** for DCL2 (Figure 3A,B, Table 1; SI, Figures S8–S10, Table S10–S12), and **13**–**19** for DCL3 (Figure 3C,D, Table 1; SI, Figures S11–S13, Table S13–S15). Notably, the most significant amplification scores were achieved with compounds bearing the 2,4-dichlorophenoxybutanoyl motif, which was confirmed as a potential privileged structure to engage interactions with RAD51.

**Figure 3:**
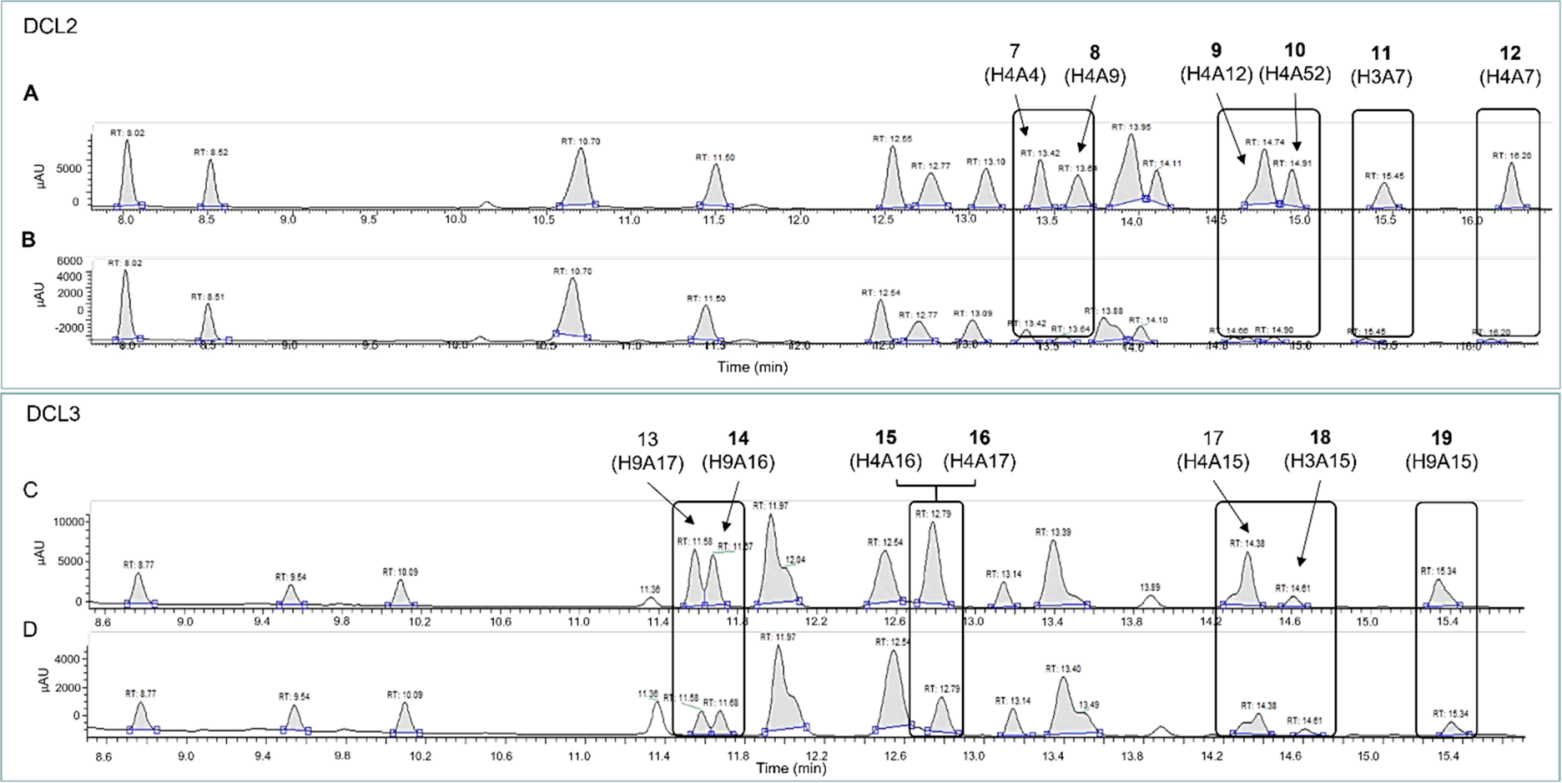
DCL2 UV chromatograms (290 nm) in protein-templated (A), and blank (B) reactions, 10 h; DCL3 UV chromatograms (290 nm), in protein-templated (C) and blank (D) reactions, 8 h.

**7**–**16** were resynthesized according to the general procedure (Scheme 2), while **17**–**19** were preliminarily discarded based on predicted physicochemical properties, which suggested remarkably poor water solubility (SI, Table S16). All synthesized NAHs were screened in the ELISA assay. Interestingly, **7**, **10**, **12**, and **16** showed improved inhibitory activities compared to DCL1 parent compounds **1**, **2**, and **5**, confirming the potential of ptDCC as optimization tool (Table 1).^25^

To further characterize the physical interaction of amplified NAHs with RAD51, we performed direct binding and displacement experiments through NMR spectroscopy in the absence and presence of recombinant RAD51 protein. The presence of a fluorine on active compounds **7**, **10**, and **12** led us to exploit ^19^F NMR spectroscopy, more sensitive to binding events than ^1^H NMR^26^ and less affected by buffer interference. Transverse relaxation experiments are based on change in transverse relaxation rate *R*_2_ of small molecules upon protein binding, resulting in line broadening of their NMR signals in the presence of the target protein. Among the available fluorinated compounds, **7** emerged as the best choice for its higher solubility in buffer. Transverse relaxation filter (^19^F T2-filter) NMR experiments have been performed on **7** in absence and in the presence of RAD51. Notably, **7** showed two ^19^F signals in solution, corresponding to interconverting *E*/*Z* rotamers (Figure 4, black), in agreement with its ^1^H and ^13^C NMR characterization (see SI). In ^19^F T2 filter experiments, both ^19^F signals showed a line broadening upon addition of RAD51, indicating a binding event (Figure 4, red). Interestingly, the two ^19^F NMR signals of **7** returned sharp to their original shape upon addition of BRC4 (Figure 4, blue), suggesting a competition between **7** and BRC4 for the same protein binding site. Given the higher affinity of BRC4 for RAD51 (nM range)^27^ compared to **7**, the compound was completely displaced by BRC4, thus no binding of **7** were visible in the presence of BRC4 (data not shown). These data suggested that **7** binds in the same pocket occupied by BRC4, already structurally characterized by Pellegrini et al.^22^

**Figure 4:**
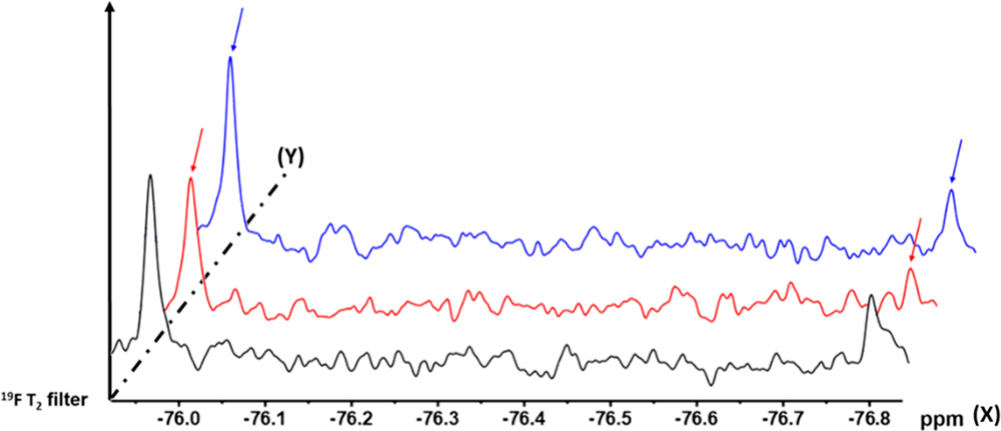
Superimposition of ^19^F T2-filter spectra of **7** (20 μM) (mixing time τ = 160 ms) in the absence (black) or in the presence of 1 μM RAD51 (red). The blue NMR spectrum was recorded after addition of 5 μM BRC4 to RAD51 (1 μM)–**7** (20 μM) mixture. Three ^19^F spectra are shifted on both *X* and *Y* axes for visualization and comparison. Chemical shifts in ppm are of **7** alone (black) spectrum and referred to CFCl_3_ signal in water.

To predict the binding mode of **7** to RAD51, we performed docking simulations in the area where BRC4 binds RAD51 (named site I and site II). Results suggested that both *E* and *Z* rotamers could bind to site I at the N-terminus domain (Figure 5), mimicking BRC4 binding mode (see SI, section 6, Figure S97), in agreement with NMR data. In particular, **7** showed to interact with RAD51 mainly by hydrophobic interactions, filling with one of the two aromatic rings of the pocket known to bind BRC4 Phe1524. In addition, depending on the considered rotamer, we could observe an H-bond with Tyr191 backbone (Figure 5B), in the place of BRC4 His1525, or with His199 and Gln206 (Figure 5D), similarly to BRC4 Leu1522 (see SI, section 6, Figure S97).

**Figure 5:**
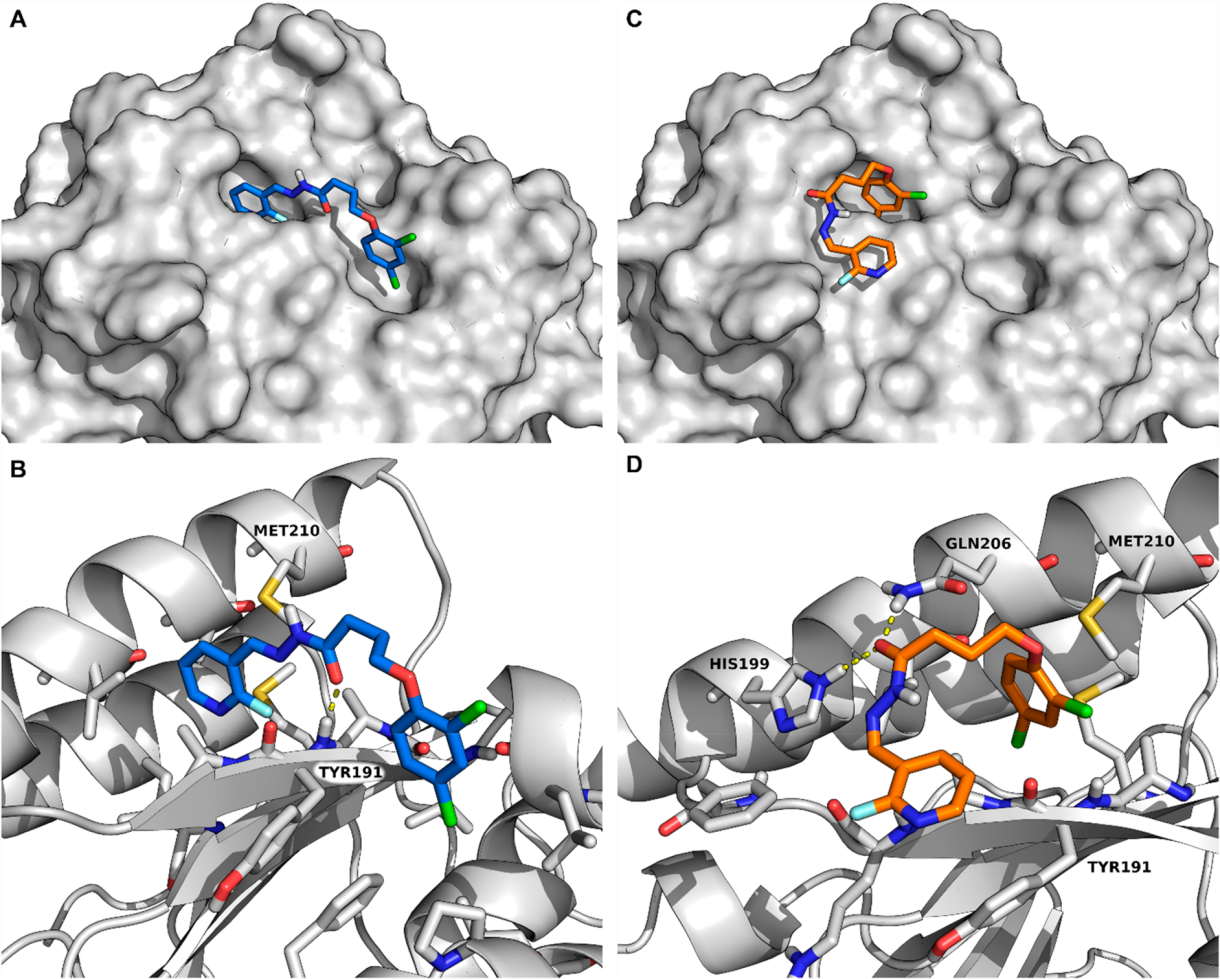
Binding mode of **7** in site I; RAD51 depicted as gray cartoon and surface, and rotamer *E* (A,B) as blue and *Z* (C,D) as orange sticks.

The self-oligomerized state of RAD51, used in DCC and ^19^F NMR experiments, is supposed to have the majority of FxxA binding pockets blocked due to their involvement in oligomer folding. Nevertheless, we do consider that the remaining RAD51 terminals can be responsible for the templating effect in ptDCC. This consideration is supported by our recent investigations,^27^ reporting that the disassembly of RAD51 fibrils is mediated by BRC4 as a gradual, monomer-by-monomer depolymerization process, starting from the available terminus of the fibril. This model gives space for the interaction of small molecules with the available terminals of RAD51 fibrils, where they can eventually lock the fibril and prevent BRC4 interaction, thoroughly inhibiting fibrils disassembly and, consequently, RAD51 activity.

RAD51–BRCA2 PPI, as described by Pellegrini and co-workers,^22^ is driven by many hydrophobic interactions. Thus, small molecules targeting the PPI interface can be tendentially hydrophobic and suffer of unfavorable physical–chemical properties. ptDCC, as an aqueous media-based technique, can potentially guide the identification of compounds with increased water solubility. Indeed, we designed DCLs including polar BBs to favor the selection of compounds containing BBs hydrophilic and identify RAD51 ligands with improved water solubility. However, ptDCC experiments revealed a remarkable trend of RAD51 in amplifying NAHs bearing lipophilic BBs, with particular preference for the 2,4-dichloro-phenoxy moiety, present also in **7**. This feature inevitably affected water solubility, limiting NAHs in further testing. Future efforts will cover the need of improved physicochemical profile of selected NAHs.

In conclusion, we reported the first application of ptDCC as hit-identification and -optimization strategy on oligomeric RAD51. Upon design of *N*-acylhydrazone-based DCLs, RAD51 acted as template protein, selecting its own ligands with high amplification factors. Remarkably, increased system complexity linked to the use of full-length wild-type protein did not prevent RAD51 applicability in ptDCC. This study opens the way to the exploitation of RAD51 as template-protein in its oligomeric full-length form, which better resembles its physiological state. Interestingly, 10 NAHs inhibited RAD51–BRC4 interaction at the biochemical level, suggesting an inhibitory activity against RAD51–BRCA2 PPI. Further biophysical and computational analysis elucidated the physical interaction of **7** with RAD51, effectively proposing RAD51–BRCA2 interface as binding site. Together these achievements supported the idea that ptDCC could guide the identification of potential RAD51–BRCA2 PPI inhibitors.

Furthering the potential of NAH-based DCC, the replacement of NAH linker with bioisosteres can be the next strategy to improve compound drug-likeness, increasing chemical stability, and reducing eventual cytotoxicity. Indeed, as hit-to-lead optimization, successful NAH replacement with amide has been reported to improve chemical stability while retaining the activity.^25,28^ Moreover, capitalizing on this study, ptDCC can be further exploited using different DCC reversible linkers and/or coupling DCL design to fragment-based screening, ultimately opening to unexplored chemical space targeting the oligomeric RAD51.

## Supporting Information Available

Stability Analysis via TSA/DSF; ptDCC: general procedure; ptDCC experiments; chemistry: general procedure for the synthesis of *N*-acylhydrazone (**1**–**16**); NMR spectra and UPLC-MS chromatograms; characterization of *E*/*Z* isomers of *N*-acylhydrazone **7** by 2D-NOESY experiment; biochemical ELISA assay: dose–response curves of compounds **1**–**16**; ^19^F-NMR binding assay protocol; computational methods: predicted physicochemical properties for **17**–**19**; docking studies: compound **7** and BRC4 overlay (PDF)

## Abbreviations

BBs: building blocks
CDAs: cytidine deaminases
DCC: dynamic combinatorial chemistry
DCL: dynamic combinatorial library
DMSO: dimethyl sulfoxide
DSB: double strand break
HR: homologous recombination
NAH: *N*-acylhydrazone
PARP: poly(ADP-ribose) polymerase
PARPi: poly(ADP-ribose) polymerase inhibitor
PPI: protein–protein interaction
ptDCC: protein-templated dynamic combinatorial chemistry
RPA: relative peak area
Rt: retention time
SBDD: structure-based drug discovery
SL: synthetic lethality
TSA/DSF: thermal-shift assay/differential scanning fluorimetry

## References

[ref1] Hine C. M.; Seluanov A.; Gorbunova V. Use of the Rad51 promoter for targeted anti-cancer therapy. Proc. Natl. Acad. Sci. U. S. A. 2008, 105, 20810–20815. 10.1073/pnas.0807990106. 19106292 PMC2634908

[ref2] Klein H. L. The consequences of Rad51 overexpression for normal and tumor cells. DNA Repair (Amst) 2008, 7, 686–693. 10.1016/j.dnarep.2007.12.008. 18243065 PMC2430071

[ref3] Sullivan M. R.; Bernstein K. A. RAD-ical New Insights into RAD51 Regulation. Genes (Basel) 2018, 9, 629 10.3390/genes9120629. PMC6316741 30551670

[ref4] Demeyer A.; Benhelli-Mokrani H.; Chenais B.; Weigel P.; Fleury F. Inhibiting homologous recombination by targeting RAD51 protein. Biochim Biophys Acta Rev. Cancer 2021, 1876, 188597 10.1016/j.bbcan.2021.188597. 34332021

[ref5] Budke B.; Lv W.; Kozikowski A. P.; Connell P. P. Recent Developments Using Small Molecules to Target RAD51: How to Best Modulate RAD51 for Anticancer Therapy?. ChemMedChem. 2016, 11, 2468–2473. 10.1002/cmdc.201600426. 27781374 PMC5472043

[ref6] Ward A.; Khanna K. K.; Wiegmans A. P. Targeting homologous recombination, new pre-clinical and clinical therapeutic combinations inhibiting RAD51. Cancer Treat Rev. 2015, 41, 35–45. 10.1016/j.ctrv.2014.10.006. 25467108

[ref7] Castro A. C.; Day M.; Maclay T.; McComas C. C.; Mills K.; Vacca J. Methods of using RAD51 inhibitors for treatment of pancreatic cancer. PCT Int. Appl. WO2020/257752, 2020.

[ref8] Deeks E. D. Olaparib: first global approval. Drugs 2015, 75, 231–240. 10.1007/s40265-015-0345-6. 25616434

[ref9] Arora S.; Balasubramaniam S.; Zhang H.; Berman T.; Narayan P.; Suzman D.; Bloomquist E.; Tang S.; Gong Y.; Sridhara R.; Turcu F. R.; Chatterjee D.; Saritas-Yildirim B.; Ghosh S.; Philip R.; Pathak A.; Gao J. J.; Amiri-Kordestani L.; Pazdur R.; Beaver J. A. FDA Approval Summary: Olaparib Monotherapy or in Combination with Bevacizumab for the Maintenance Treatment of Patients with Advanced Ovarian Cancer. Oncologist 2021, 26, e164–e172. 10.1002/onco.13551. 33017510 PMC7794199

[ref10] Golan T.; Hammel P.; Reni M.; Van Cutsem E.; Macarulla T.; Hall M. J.; Park J. O.; Hochhauser D.; Arnold D.; Oh D. Y.; Reinacher-Schick A.; Tortora G.; Algül H.; O’Reilly E. M.; McGuinness D.; Cui K. Y.; Schlienger K.; Locker G. Y.; Kindler H. L. Maintenance Olaparib for Germline BRCA-Mutated Metastatic Pancreatic Cancer. N Engl J. Med. 2019, 381, 317–327. 10.1056/NEJMoa1903387. 31157963 PMC6810605

[ref11] Falchi F.; Giacomini E.; Masini T.; Boutard N.; Di Ianni L.; Manerba M.; Farabegoli F.; Rossini L.; Robertson J.; Minucci S.; Pallavicini I.; Di Stefano G.; Roberti M.; Pellicciari R.; Cavalli A. Synthetic Lethality Triggered by Combining Olaparib with BRCA2-Rad51 Disruptors. ACS Chem. Biol. 2017, 12, 2491–2497. 10.1021/acschembio.7b00707. 28841282

[ref12] Roberti M.; Schipani F.; Bagnolini G.; Milano D.; Giacomini E.; Falchi F.; Balboni A.; Manerba M.; Farabegoli F.; De Franco F.; Robertson J.; Minucci S.; Pallavicini I.; Di Stefano G.; Girotto S.; Pellicciari R.; Cavalli A. Rad51/BRCA2 disruptors inhibit homologous recombination and synergize with olaparib in pancreatic cancer cells. Eur. J. Med. Chem. 2019, 165, 80–92. 10.1016/j.ejmech.2019.01.008. 30660828

[ref13] Scott D. E.; Francis-Newton N. J.; Marsh M. E.; Coyne A. G.; Fischer G.; Moschetti T.; Bayly A. R.; Sharpe T. D.; Haas K. T.; Barber L.; Valenzano C. R.; Srinivasan R.; Huggins D. J.; Lee M.; Emery A.; Hardwick B.; Ehebauer M.; Dagostin C.; Esposito A.; Pellegrini L.; Perrior T.; McKenzie G.; Blundell T. L.; Hyvönen M.; Skidmore J.; Venkitaraman A. R.; Abell C. A small-molecule inhibitor of the BRCA2-RAD51 interaction modulates RAD51 assembly and potentiates DNA damage-induced cell death. Cell Chem. Biol. 2021, 28, 835 10.1016/j.chembiol.2021.02.006. 33662256 PMC8219027

[ref14] Bagnolini G.; Milano D.; Manerba M.; Schipani F.; Ortega J. A.; Gioia D.; Falchi F.; Balboni A.; Farabegoli F.; De Franco F.; Robertson J.; Pellicciari R.; Pallavicini I.; Peri S.; Minucci S.; Girotto S.; Di Stefano G.; Roberti M.; Cavalli A. Synthetic Lethality in Pancreatic Cancer: Discovery of a New RAD51-BRCA2 Small Molecule Disruptor That Inhibits Homologous Recombination and Synergizes with Olaparib. J. Med. Chem. 2020, 63, 2588–2619. 10.1021/acs.jmedchem.9b01526. 32037829 PMC7997579

[ref15] Mondal M.; Hirsch A. K. Dynamic combinatorial chemistry: a tool to facilitate the identification of inhibitors for protein targets. Chem. Soc. Rev. 2015, 44, 2455–2488. 10.1039/C4CS00493K. 25706945

[ref16] Frei P.; Hevey R.; Ernst B. Dynamic Combinatorial Chemistry: A New Methodology Comes of Age. Chemistry 2019, 25, 60–73. 10.1002/chem.201803365. 30204930

[ref17] Canal-Martin A.; Perez-Fernandez R. Protein-Directed Dynamic Combinatorial Chemistry: An Efficient Strategy in Drug Design. ACS Omega 2020, 5, 26307–26315. 10.1021/acsomega.0c03800. 33110958 PMC7581073

[ref18] Hartman A. M.; Gierse R. M.; Hirsch A. K. H. Protein-Templated Dynamic Combinatorial Chemistry: Brief Overview and Experimental Protocol. Eur. J. Org. Chem. 2019, 2019, 3581–3590. 10.1002/ejoc.201900327. PMC6813629 31680778

[ref19] Ramstrom O.; Lehn J. M. Drug discovery by dynamic combinatorial libraries. Nat. Rev. Drug Discov 2002, 1, 26–36. 10.1038/nrd704. 12119606

[ref20] Karan I. I.; Miller B. L. Dynamic diversity in drug discovery: Putting small-molecule evolution to work. Drug Discov Today 2000, 5, 67–75. 10.1016/S1359-6446(99)01431-2. 10652457

[ref21] Hartman A. M.; Elgaher W. A. M.; Hertrich N.; Andrei S. A.; Ottmann C.; Hirsch A. K. H. Discovery of Small-Molecule Stabilizers of 14–3-3 Protein-Protein Interactions via Dynamic Combinatorial Chemistry. ACS Med. Chem. Lett. 2020, 11, 1041–1046. 10.1021/acsmedchemlett.9b00541. 32435423 PMC7236542

[ref22] Pellegrini L.; Yu D. S.; Lo T.; Anand S.; Lee M.; Blundell T. L.; Venkitaraman A. R. Insights into DNA recombination from the structure of a RAD51-BRCA2 complex. Nature 2002, 420, 287–293. 10.1038/nature01230. 12442171

[ref23] Rajendra E.; Venkitaraman A. R. Two modules in the BRC repeats of BRCA2 mediate structural and functional interactions with the RAD51 recombinase. Nucleic Acids Res. 2010, 38, 82–96. 10.1093/nar/gkp873. 19875419 PMC2800230

[ref24] Bhat V. T.; Caniard A. M.; Luksch T.; Brenk R.; Campopiano D. J.; Greaney M. F. Nucleophilic catalysis of acylhydrazone equilibration for protein-directed dynamic covalent chemistry. Nat. Chem. 2010, 2, 490–497. 10.1038/nchem.658. 20489719 PMC2913121

[ref25] Jumde R. P.; Guardigni M.; Gierse R. M.; Alhayek A.; Zhu D.; Hamid Z.; Johannsen S.; Elgaher A. M.W.; Neusens P. J.; Nehls C.; Haupenthal J.; Reiling N.; Hirsch A. K. H. Hit-optimization using target-directed dynamic combinatorial chemistry: development of inhibitors of the anti-infective target 1-deoxy-d-xylulose-5-phosphate synthase. Chem. Sci. 2021, 12, 7775–7785. 10.1039/D1SC00330E. 34168831 PMC8188608

[ref26] Dalvit C. NMR methods in fragment screening: theory and a comparison with other biophysical techniques. Drug Discov Today 2009, 14, 1051–1057. 10.1016/j.drudis.2009.07.013. 19716431

[ref27] Schipani F. M. M.; Marotta R.; Gennari A.; Rinaldi F.; Armirotti A.; Roberti R.; Di Stefano G.; Rocchia W.; Tirelli N.; Girotto S.; Cavalli A. RAD51 nuclear recruitment and inhibition towards innovative strategies against pancreatic cancer. bioRxiv 2021, 429564, DOI: 10.1101/2021.02.03.429564.

[ref28] Jumde V. R.; Mondal M.; Gierse R. M.; Unver M. Y.; Magari F.; van Lier R. C. W.; Heine A.; Klebe G.; Hirsch A. K. H. Design and Synthesis of Bioisosteres of Acylhydrazones as Stable Inhibitors of the Aspartic Protease Endothiapepsin. ChemMedChem. 2018, 13, 2266–2270. 10.1002/cmdc.201800446. 30178575 PMC6282583

