# Supplementary material for: Identification of RAD51–BRCA2 Inhibitors Using N-Acylhydrazone-Based Dynamic Combinatorial Chemistry
Source: ACS Med Chem Lett. 2022 Jul 28;13(8):1262–9. doi: 10.1021/acsmedchemlett.2c00063 (PMC9377020; doi:10.1021/acsmedchemlett.2c00063)
Supplement: Supplementary file 1 — ml2c00063_si_001.pdf [file ml2c00063_si_001.pdf]

SUPPORTING INFORMATION

Identification of RAD51-BRCA2 inhibitors using *N*-acylhydrazone-based dynamic combinatorial chemistry

Greta Bagnolini[1,2,3], Beatrice Balboni[1,2], Fabrizio Schipani[1], Dario Gioia,[1] Marina Veronesi[4,5], Francesca De Franco[6], Cansu Kaya[3,7], Ravindra P. Jumde[3], Jose Antonio Ortega[1], Stefania Girotto[1], Anna K. H. Hirsch[3,7*], Marinella Roberti[2*], and Andrea Cavalli[1,2*]

[1]Computational & Chemical Biology (CCB), Istituto Italiano di Tecnologia (IIT), 16163 Genova, Italy;
[2]Department of Pharmacy and Biotechnology (FaBiT), University of Bologna, 40126 Bologna, Italy;
[3]Helmholtz Institute for Pharmaceutical Research Saarland (HIPS) − Helmholtz Centre for Infection Research (HZI), 66123 Saarbrücken, Germany;
[4]Structural Biophysics and Translational Pharmacology, Istituto Italiano di Tecnologia (IIT), 16163 Genova, Italy;
[5]D3-PharmaChemistry, Istituto Italiano di Tecnologia (IIT), 16163 Genova, Italy
[6]TES Pharma S.r.l., I-06073 Corciano, Perugia, Italy;
[7]Department of Pharmacy, Saarland University, 66123 Saarbrücken, Germany.

Corresponding author
*Anna K. H. Hirsch - E-mail: anna.hirsch@helmholtz-hips.de
*Marinella Roberti - E-mail: marinella.roberti@unibo.it
*Andrea Cavalli - E-mail: andrea.cavalli@iit.it

Contents

Authors contributions 3

1. Stability Analysis *via* TSA/DSF 4

2. ptDCC 6

General Procedures (GPs) 6

ptDCC Experiments 8

3. Chemistry 23

Material and Methods. 23

General Procedure for the Synthesis of *N*-acylhydrazone (1−16) 24

NMR spectra and UPLC-MS chromatograms 33

Characterization of *E*/*Z* isomers of *N*-acylhydrazone 7 *via* 2D-NOESY experiment 82

4. Biochemical ELISA assay: dose-response curves of compounds 1─16 83

5. ^{19}F-NMR binding assay protocol 84

6. Computational methods 85

Predicted physicochemical properties for 17–19 85

Docking studies: compound 7 and BRC4 overlay 87

Supplementary References 88

Abbreviations 89

Authors contributions

G. Bagnolini was involved in the design of the project, performing the ptDCC experiments, synthesis of compounds and writing of the manuscript. B. Balboni were involved in the binding study *via* ^{19}F-NMR assay. F. Schipani was involved in the expression and purification of RAD51 protein. D. Gioia was involved in molecular docking studies of compounds. M. Veronesi were involved in the binding study *via* ^{19}F-NMR assay. F. De Franco was involved in the evaluation of PPI inhibition *via* biochemical ELISA assay. C. Kaya was involved in the assessment of RAD51 stability *via* TSA/DSF assay. R. P. Jumde was involved in the supervision of the project. J.A. Ortega was involved in the characterization and QC of compounds. S. Girotto was involved in the expression and purification of RAD51 protein and in the binding study *via* ^{19}F-NMR. M. Roberti, A. K. H. Hirsch and A. Cavalli were involved in the design and supervision of the project and editing the manuscript.

1. Stability Analysis *via* TSA/DSF

Protein expression and purification

Recombinant human RAD51 was expressed and purified as previously described.[1]

Determination of RAD51 Stability in different buffer systems. Prior to DCC experiments, the stability of RAD51 in different buffer conditions were monitored over the course of 2 days by measuring its melting temperature (T_m) using thermal shift assay (TSA). First, the final concentration of the RAD51 and SYPRO orange 5000x dye (Sigma-Aldrich) were optimized by screening different concentrations of RAD51 (2.5, 5, 10.7 mg/mL) at different dye concentrations (10X, 5X, 2.5X). The experiments were performed in a 96-well PCR plate (Thermoscientific). The final volume per well was 25 µL, consisting of 18.75 µL of protein buffer 20 mM HEPES, pH: 7.5, 300 mM KCl, 0.1 mM EDTA, % 10 Glycerol, 2mM DTT, 2.5 µL of SYPRO orange dye, 2.5 µL of RAD51 enzyme and 5 % DMSO (1.25µL). The plate was centrifuged for 2 minutes at room temperature and the melting temperature of the protein was measured using a Real-time PCR machine (Step one plus, Applied Biosystem). The conditions of the experiment were adjusted using Step One 2.3 software. The starting temperature, the ending temperature and the heating rate were set as 21 °C, 95 °C and 0.5 °C / min, respectively. The melting curves were analysed using Protein Thermal Shift 1.3 software. Most suitable protein and dye concentrations were determined as 5 mg/mL and 10X, respectively. T_m of the RAD51 under these conditions was found 44,5 °C.

These concentrations were used to monitor the stability of RAD51 in four different buffers (Acetate, MES, BIS-Tris, Phosphate) with differing pH ranges, together with water and RAD51 buffer. Three plates for days 0, 1 and 2 were prepared, measured and the melting curves were analysed as described above (25 µL total volume, 5% final DMSO). The plates were incubated at room temperature and the change in the T_m was measured for each plate after every 24h over 2 days (Figure S1). The protein remained stable under the conditions Bis-Tris pH: 6, 6.5, MES pH: 6, 6.5, Acetate pH: 5.5, 7, 7.5 and in original protein buffer (20 mM HEPES, pH: 7.5, 300 mM KCl, 0.1 mM EDTA, % 10 Glycerol, 2mM DTT).

Figure S1. Melting temperature of RAD51 in different buffer conditions at room temperature over 3 days.

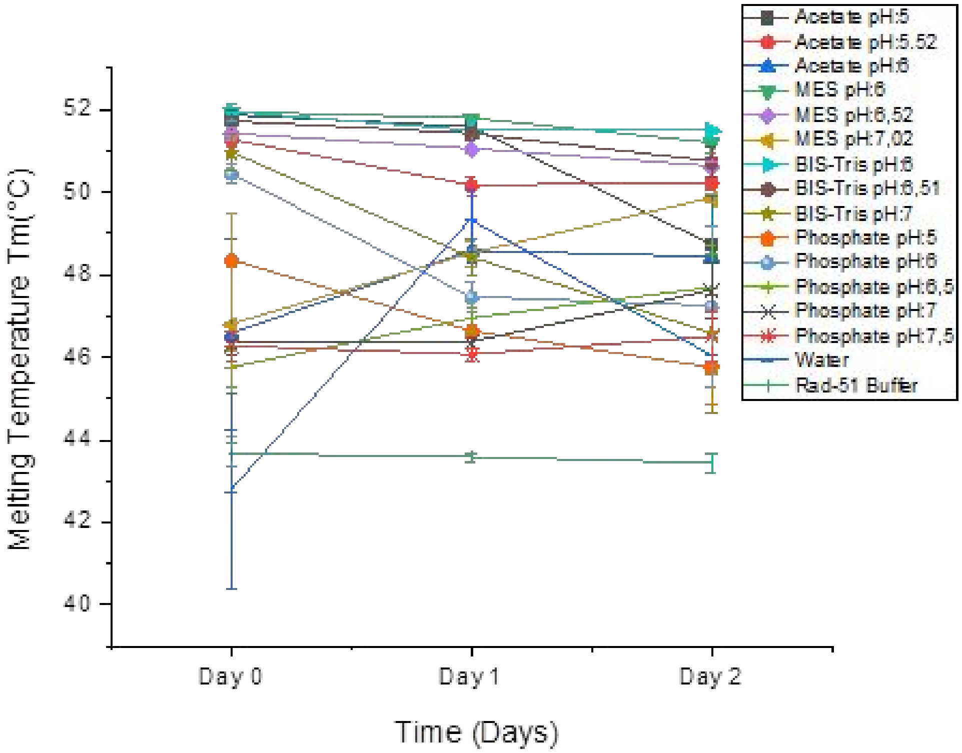

2. ptDCC

Protein expression and purification

Recombinant human RAD51 was expressed and purified as previously described.[1]

UPLC-MS analysis in tdDCC. The periodic progress and analysis of DCC were monitored on UPLC-MS (ThermoScientific Dionex Ultimate 3000 UHPLC System coupled to a ThermoScientific Q Exactive Focus with an electrospray ion source). An Acquity Waters Column (BEH C8, 1.7 µm, 2.1 x 150 mm, Waters, Germany) equipped with a VanGuard Pre-Column (BEH C8, 1.7 µm, 5 x 2.1 mm, Waters, Germany) was used for the separation. At a flow rate of 0.25 mL/min, the gradient of H2O (0.1% formic acid) and acetonitrile (0.1% formic acid) was held at 5% acetonitrile for 1 min and then increased to 95% over 16 min. It was held there for 1.5 min before the gradient was decreased to 5% over 0.1 min where it was held for 1.9 min. Detection was set at 290 nm and the mass spectrum was measured in a positive mode in the range of 100-700 m/z.

General Procedures (GPs)

GP1 - DCL preparation

To a 1.5 mL Eppendorf Tube® containing HEPES buffer (pH 7.02, KCl 300 mM, glycerol 5%) was added hydrazides (300−1000 µM each, in DMSO), aldehydes (100 µM each, in DMSO), and aniline (10 mM, in DMSO) with 250 µL of end-volume. The DCL was allowed to mix gently on a rotating wheel (7 rpm) at room temperature and was frequently monitored via UPLC-MS. For analysis, 10 µL of the corresponding library was mixed with 90µL methanol and 2µL of NaOH (2 M), the mixture was centrifuged at 14.000 rpm for 8 min and the supernatant was used for the analysis.

GP2 - Protein-templated DCL preparation

To a 1.5 mL Eppendorf Tube® containing HEPES buffer (pH 7.02, KCl 300 mM, glycerol 5%) was added hydrazides (300−1000 µM each, in DMSO), aldehydes (100 µM each, in DMSO), aniline (10 mM, in DMSO), and the protein RAD51 (17.2 µM in buffer HEPES 20 mM, pH 7.54, KCl 300 mM, glycerol 10%, EDTA 0.1 mM, DTT 2.0 mM) with 250 µL of end-volume. The DCL with the protein was allowed to mix gently on a rotating wheel (7 rpm) at room temperature and was

frequently monitored via UPLC-MS and the traces were compared with the blank composition. For analysis, 10 µL of the corresponding library was mixed with 90µL methanol and 2µL of NaOH (2 M), the mixture was centrifuged at 14.000 rpm for 8 min and the supernatant was used for the analysis. The protein-templated DCL were run as duplicates.

Assessment of DCL composition

The library composition was assessed after the equilibrium of acylhydrazone formation was reached as reported in the literature.[2,3] The "amplification factor" determined with the relative peak area (RPA), which is the percent of each peak when the sum of all peak areas was set to 100%. The "normalized RPA" was used for the final assessment of amplification of the acylhydrazone products in the DCL.

$$amplification\ factor\% = RPA\ (templated)/RPA\ (blank)*100$$

$$normalized\ change\ of\ RPA = (RPA(templated) - RPA(blank))/RPA(blank)$$

ptDCC Experiments

DCL1

This experiment library DCL1 consists of three aldehydes (**A1−A3**) and eight hydrazides (**H1−H8**). The DCC-experiment was carried out according to the general procedure-1 (blank) and general procedure-2 (protein-templated) in HEPES 20 mM (pH 7.54, KCl 300 mM, glycerol 10%, EDTA 0.1 mM, DTT 2 mM) (Table S1).

Table S1. DCL1 composition and final concentrations.

Entry	Blank		Protein-templated (I)		Protein-templated (II)	
	Amount	Final concentration	Amount	Final concentration	Amount	Final concentration
Buffer	240.75 µL	-	140.75 µL	-	140.75 µL	-
Hydrazide (100 mM)	8 x 0.75 µL	0.3 mM	8 x 0.75 µL	0.3 mM	8 x 0.75 µL	0.3 mM
Aldehyde (100 mM)	3 x 0.25 µL	0.1 mM	3 x 0.25 µL	0.1 mM	3 x 0.25 µL	0.1 mM
Aniline (1M)	2.5 µL	10 mM	2.5 µL	10 mM	2.5 µL	10 mM
DMSO	9.25 µL	3.7%	9.25 µL	3.7%	9.25 µL	3.7%
RAD51 (43 µM)	0	-	100 µL	17.2 µM	100 µL	17.2 µM
Tot volume	250 µL	-	250 µL	-	250 µL	-

The DCL1 was left shaking at room temperature and was monitored at 8 h, 24 h, 48 h, and 72 h *via* UPLC-MS.

Figure S2. Evaluation of the equilibrium state of DCL1 blank reaction.

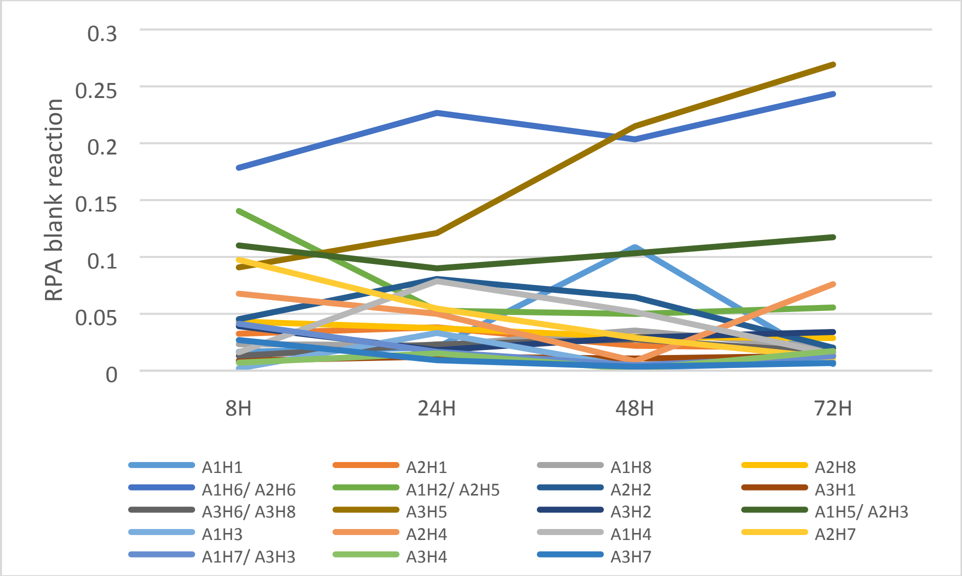

Table S2. RPAs values in blank reaction DCL1.

Apex RT	Cpd	8H	24H	48H	72H
5.59	**A1H1**	0.016612	0.020339	0.1088	0.005814
5.84	**A2H1**	0.032488	0.038084	0.021878	0.020202
5.99	**A1H8**	0.023296	0.022322	0.035266	0.019763
6.37	**A2H8**	0.043209	0.03734	0.029626	0.028679
6.55	**A1H6/ A2H6**	0.178385	0.226683	0.203397	0.243293
6.88	**A1H2/ A2H5**	0.140402	0.052765	0.049886	0.055537
7.04	**A2H2**	0.045261	0.080592	0.064565	0.020147
7.31	**A3H1**	0.008807	0.012415	0.010663	0.013129
7.71	**A3H6/ A3H8**	0.013064	0.023048	0.027474	0.016148
8.09	**A3H5**	0.090835	0.121033	0.214991	0.269199
8.33	**A3H2**	0.039097	0.018064	0.029111	0.033988
8.89	**A1H5/ A2H3**	0.110098	0.089969	0.103252	0.117358
9.05	**A1H3**	0.002004	0.033143	0.002556	0.007496
9.26	**A2H4**	0.067667	0.049967	0.00843	0.076065
9.47	**A1H4**	0.016129	0.078704	0.051641	0.014418
9.79	**A2H7**	0.097458	0.054591	0.028903	0.011223
10.07	**A1H7/ A3H3**	0.041245	0.016541	0.004767	0.012706
10.4	**A3H4**	0.007037	0.015193	0.001331	0.017209
10.85	**A3H7**	0.026906	0.009208	0.003462	0.006723

Figure S3. Evaluation of the composition of protein-templated reaction DCL1 (exp in duplicate).

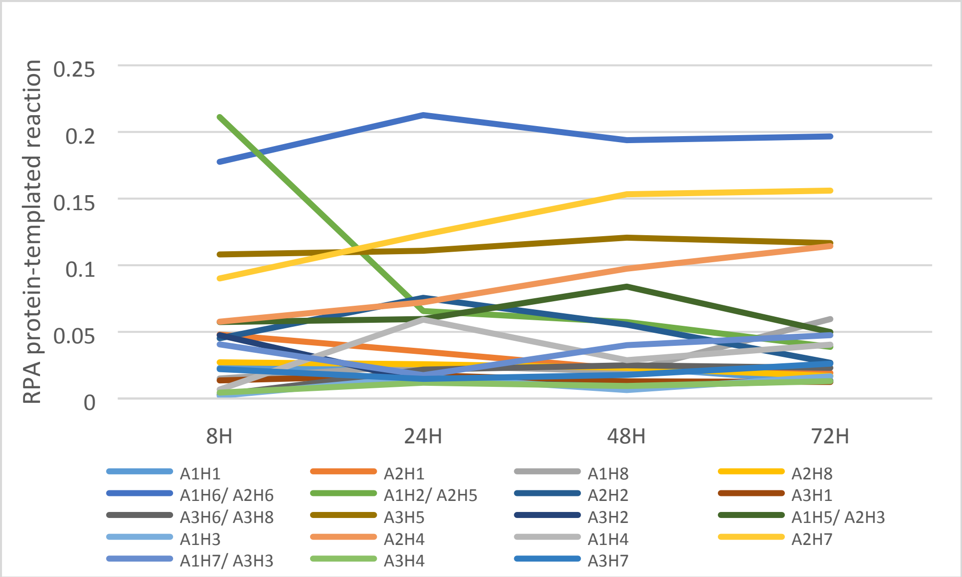

Table S3. RPAs values in protein-templated reaction DCL1 (exp in duplicate).

Apex RT	Cpd	8H	24H	48H	72H
5.59	**A1H1**	0.022874	0.023435	0.023631	0.012517
5.84	**A2H1**	0.047973	0.03517	0.021889	0.019224
5.99	**A1H8**	0.01504	0.024764	0.020338	0.059608
6.37	**A2H8**	0.027153	0.025726	0.023045	0.016968
6.55	**A1H6/ A2H6**	0.177581	0.212647	0.19384	0.196654
6.88	**A1H2/ A2H5**	0.211193	0.065606	0.05736	0.038736
7.04	**A2H2**	0.045218	0.075536	0.055414	0.026801
7.31	**A3H1**	0.013578	0.017553	0.012886	0.012408
7.71	**A3H6/ A3H8**	0.00281	0.021681	0.024905	0.023019
8.09	**A3H5**	0.108089	0.110845	0.120694	0.116632
8.33	**A3H2**	0.047525	0.012804	0.009142	0.013461
8.89	**A1H5/ A2H3**	0.057377	0.05963	0.083957	0.049861
9.05	**A1H3**	0.002021	0.016283	0.006345	0.016669
9.26	**A2H4**	0.057648	0.072189	0.097399	0.114361
9.47	**A1H4**	0.006743	0.059183	0.028763	0.040404
9.79	**A2H7**	0.090077	0.122844	0.153267	0.155989
10.07	**A1H7/ A3H3**	0.040614	0.017524	0.04003	0.04752
10.4	**A3H4**	0.004432	0.011845	0.009396	0.013028
10.85	**A3H7**	0.022053	0.014734	0.017698	0.026139

Figure S4. Amplification factor % for DCL1.

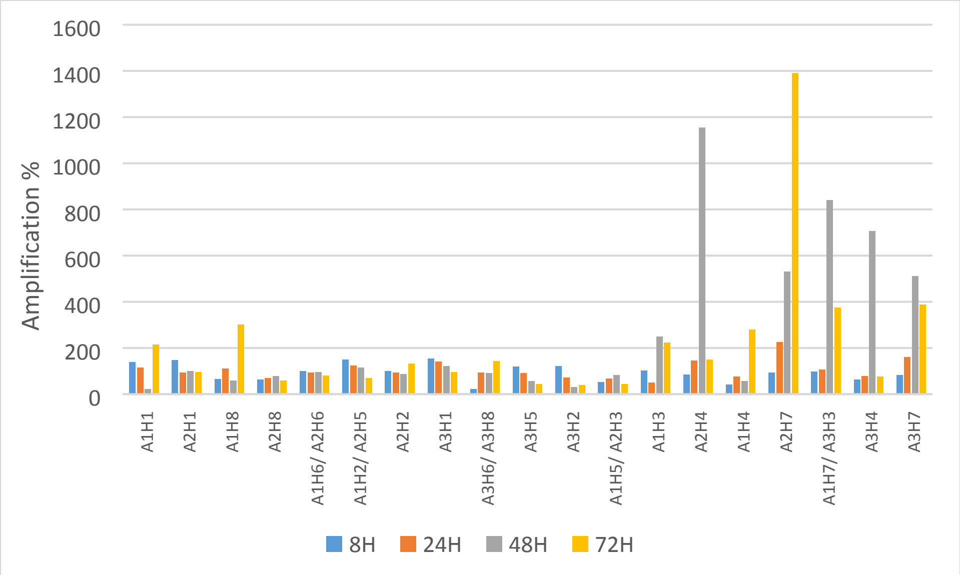

Table S4. Amplification factor % values for DCL1.

Apex RT	Cpd	8H	24H	48H	72H
5.59	**A1H1**	137.6972	115.2247	21.71995	215.3017
5.84	**A2H1**	147.6629	92.34798	100.0514	95.15978
5.99	**A1H8**	64.55988	110.9377	57.67022	301.6063
6.37	**A2H8**	62.84076	68.89527	77.78438	59.16628
6.55	**A1H6/ A2H6**	99.5495	93.80789	95.30142	80.82999
6.88	**A1H2/ A2H5**	150.4206	124.3379	114.9809	69.74821
7.04	**A2H2**	99.90653	93.72708	85.82596	133.0322
7.31	**A3H1**	154.1737	141.3915	120.8498	94.51108
7.71	**A3H6/ A3H8**	21.51129	94.06994	90.64883	142.5495
8.09	**A3H5**	118.9955	91.58275	56.1391	43.32568
8.33	**A3H2**	121.5561	70.87841	31.40322	39.60567
8.89	**A1H5/ A2H3**	52.11413	66.27827	81.31267	42.48647
9.05	**A1H3**	100.8309	49.1301	248.2516	222.3732
9.26	**A2H4**	85.19432	144.4726	1155.353	150.3463
9.47	**A1H4**	41.80322	75.19753	55.69828	280.2374
9.79	**A2H7**	92.42653	225.0259	530.2877	1389.939
10.07	**A1H7/ A3H3**	98.47054	105.9466	839.7844	374.0002
10.4	**A3H4**	62.98086	77.96477	705.8046	75.70586
10.85	**A3H7**	81.96345	160.0156	511.2559	388.8087

Figure S5. Normalized change of RPA for DCL1.

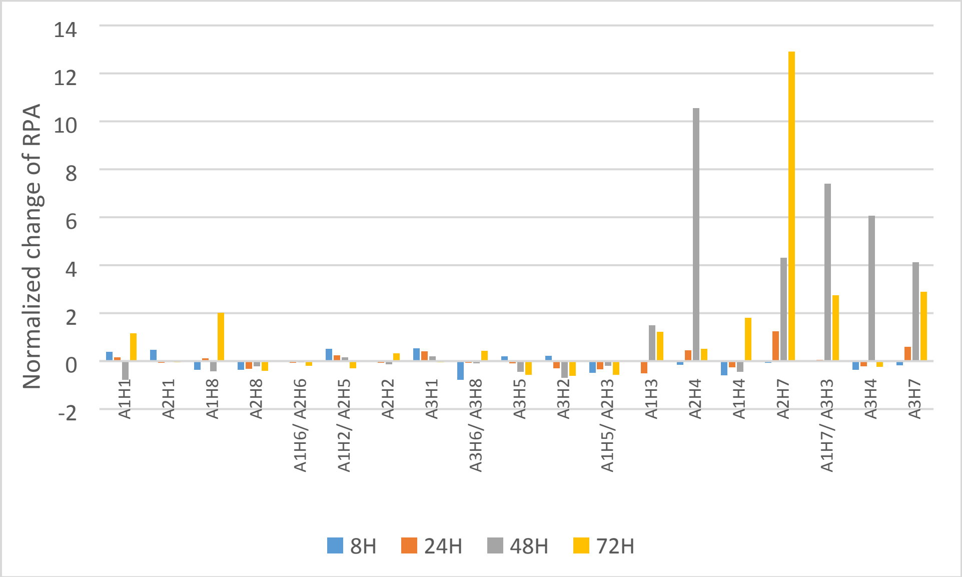

Table S5. Normalized change values of RPA for DCL1.

Apex RT	Cpd	8H	24H	48H	72H
5.59	**A1H1**	0.376972	0.152247	-0.7828	1.153017
5.84	**A2H1**	0.476629	-0.07652	0.000514	-0.0484
5.99	**A1H8**	-0.3544	0.109377	-0.4233	2.016063
6.37	**A2H8**	-0.37159	-0.31105	-0.22216	-0.40834
6.55	**A1H6/ A2H6**	-0.00451	-0.06192	-0.04699	-0.1917
6.88	**A1H2/ A2H5**	0.504206	0.243379	0.149809	-0.30252
7.04	**A2H2**	-0.00093	-0.06273	-0.14174	0.330322
7.31	**A3H1**	0.541737	0.413915	0.208498	-0.05489
7.71	**A3H6/ A3H8**	-0.78489	-0.0593	-0.09351	0.425495
8.09	**A3H5**	0.189955	-0.08417	-0.43861	-0.56674
8.33	**A3H2**	0.215561	-0.29122	-0.68597	-0.60394
8.89	**A1H5/ A2H3**	-0.47886	-0.33722	-0.18687	-0.57514
9.05	**A1H3**	0.008309	-0.5087	1.482516	1.223732
9.26	**A2H4**	-0.14806	0.444726	10.55353	0.503463
9.47	**A1H4**	-0.58197	-0.24802	-0.44302	1.802374
9.79	**A2H7**	-0.07573	1.250259	4.302877	12.89939
10.07	**A1H7/ A3H3**	-0.01529	0.059466	7.397844	2.740002
10.4	**A3H4**	-0.37019	-0.22035	6.058046	-0.24294
10.85	**A3H7**	-0.18037	0.600156	4.112559	2.888087

DCL2

This experiment library DCL2 consists of ten aldehydes (**A4**−**A13**) and two *N*-acylhydrazides (**H3**−**H4**). The DCC-experiment was carried out according to the general procedure-1 (blank) and general procedure-2 (protein-templated) in HEPES 20 mM (pH 7.02, KCl 300 mM, glycerol 5%) (*Table S6*).

Table S6. DCL2 composition and final concentrations.

Entry	Blank		Protein-templated (I)		Protein-templated (II)	
	Amount	Final concentration	Amount	Final concentration	Amount	Final concentration
Buffer	242.50 µL	-	142.50 µL	-	142.50 µL	-
Hydrazide (100 mM)	2 x 2.50 µL	1.0 mM	2 x 2.50 µL	1.0 mM	2 x 2.50 µL	1.0 mM
Aldehyde (100 mM)	10 x 0.25 µL	0.1 mM	10 x 0.25 µL	0.1 mM	10 x 0.25 µL	0.1 mM
Aniline (1M)	2.5 µL	10 mM	2.5 µL	10 mM	2.5 µL	10 mM
DMSO	10.0 µL	4.0%	10.0 µL	4.0%	10.0 µL	4.0%
RAD51 (43 µM)	0	-	100 µL	17.2 µM	100 µL	17.2 µM
Tot volume	250 µL	-	250 µL	-	250 µL	-

The DCL2 was left shaking at room temperature and was monitored at 0 h, 2 h, 4 h, 6 h, 8 h, and 10 h *via* UPLC-MS.

DCL3

This experiment library DCL3 consists of six aldehydes (**A14**−**A19**) and three *N*-acylhydrazides (**H3**−**H4**, **H9**). The DCC-experiment was carried out according to the general procedure-1 (blank) and general procedure-2 (protein-templated) in HEPES 20 mM (pH 7.02, KCl 300 mM, glycerol 5%) (Table S7).

Table S7. DCL3 composition and final concentrations.

Entry	Blank		Protein-templated (I)		Protein-templated (II)	
	Amount	Final concentration	Amount	Final concentration	Amount	Final concentration
Buffer	238.25 µL	-	138.25 µL	-	138.25 µL	-
Hydrazide (100 mM)	3 x 1.75 µL	0.7 mM	3 x 1.75 µL	0.7 mM	3 x 1.75 µL	0.7 mM
Aldehyde (100 mM)	6 x 0.25 µL	0.1 mM	6 x 0.25 µL	0.1 mM	6 x 0.25 µL	0.1 mM
Aniline (1M)	2.5 µL	10 mM	2.5 µL	10 mM	2.5 µL	10 mM
DMSO	9.25 µL	3.7%	9.25 µL	3.7%	9.25 µL	3.7%
RAD51 (43 µM)	0	-	100 µL	17.2 µM	100 µL	17.2 µM
Tot volume	250 µL	-	250 µL	-	250 µL	-

The DCL3 was left shaking at room temperature and was monitored at 0h, 2h, 4h, 6h, and 8h *via* UPLC-MS.

Figure S6. Evaluation of the equilibrium state of DCL2 blank reaction.

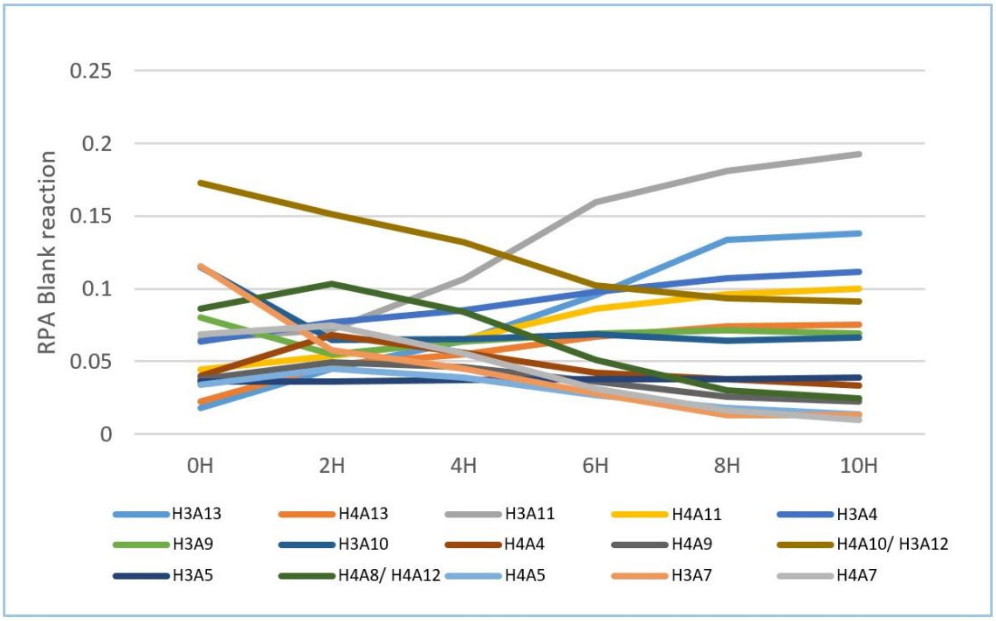

Table S8. RPAs values in blank reaction DCL2.

Apex RT (min)	Cpd	RPA 0H	RPA 2H	RPA 4H	RPA 6H	RPA 8H	RPA 10H
8.02	**H3A13**	0.01779	0.0449	0.06417	0.09587	0.13378	0.13829
8.51	**H4A13**	0.0222	0.04751	0.05487	0.06684	0.07429	0.07504
10.7	**H3A11**	0.06545	0.07216	0.10644	0.1596	0.18094	0.19252
11.5	**H4A11**	0.04418	0.05351	0.06556	0.08637	0.09633	0.10006
12.55	**H3A4**	0.06391	0.07679	0.08497	0.09777	0.1071	0.1117
12.77	**H3A9**	0.08036	0.05516	0.06360	0.069	0.07138	0.069
13.1	**H3A10**	0.11491	0.06482	0.06548	0.06843	0.06409	0.06657
13.41	**H4A4**	0.03982	0.06818	0.05605	0.04247	0.03776	0.03332
13.64	**H4A9**	0.03791	0.04912	0.04617	0.03638	0.0255	0.02232
13.95	**H4A10/ H3A12**	0.17279	0.15124	0.13231	0.10217	0.09333	0.09137
14.1	**H3A5**	0.03595	0.03596	0.03705	0.03777	0.03775	0.03907
14.74	**H4A8/ H4A12**	0.08635	0.10335	0.08389	0.05126	0.03005	0.02453
14.9	**H4A5**	0.03391	0.04485	0.03915	0.0266	0.01804	0.01355
15.45	**H3A7**	0.11577	0.05763	0.04477	0.02812	0.01319	0.01312
16.2	**H4A7**	0.06869	0.07484	0.05545	0.03135	0.01647	0.00953

Figure S7. Evaluation of the equilibrium state of DCL3 blank reaction.

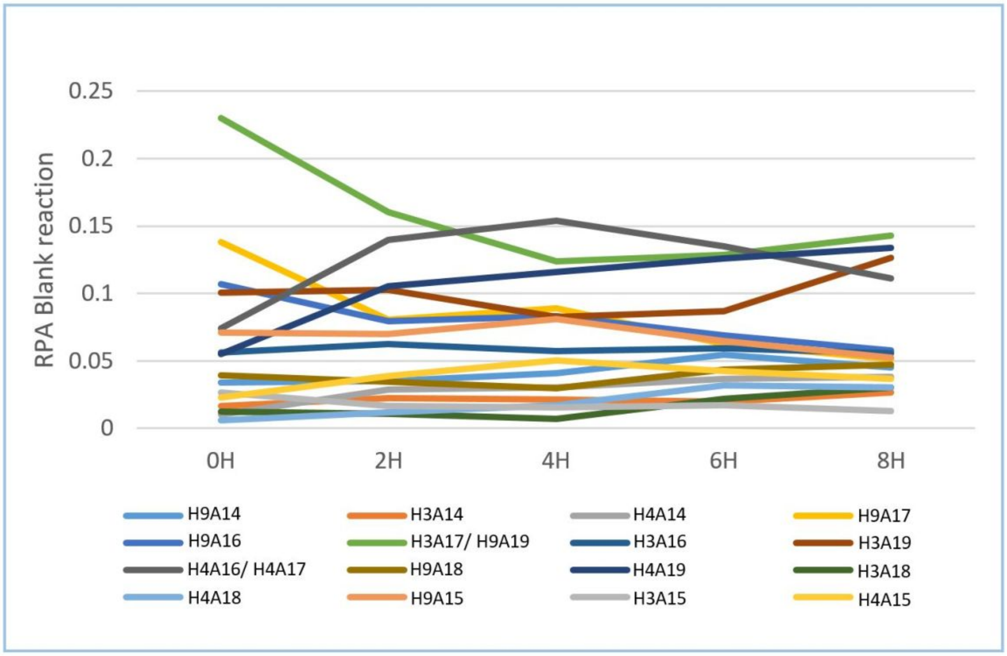

Table S9. RPAs values in blank reaction DCL2.

Apex RT (min)	Cpd	0H	2H	4H	6H	8H
9,04	**H9A14**	0.034153	0.035344	0.040752	0.054451	0.044923
9,86	**H3A14**	0.016396	0.022215	0.021603	0.019813	0.026782
10,44	**H4A14**	0.0101	0.028549	0.030511	0.036646	0.03798
12,02	**H9A17**	0.138254	0.080455	0.089135	0.062384	0.051656
12,12	**H9A16**	0.106737	0.079541	0.08334	0.068729	0.057642
12,42	**H3A17/ H9A19**	0.230365	0.160563	0.124022	0.128861	0.143183
12,52	**H3A16**	0.056447	0.062314	0.0571	0.059601	0.055841
13,03	**H3A19**	0.10053	0.102916	0.082608	0.086995	0.126495
13,30	**H4A16/ H4A17**	0.074282	0.139804	0.154041	0.134776	0.111395
13,67	**H9A18**	0.039265	0.034398	0.029545	0.043711	0.047048
13,95	**H4A19**	0.054885	0.105664	0.116226	0.126097	0.134088
14,04	**H3A18**	0.012631	0.010641	0.007193	0.021651	0.030077
15,14	**H4A18**	0.005757	0.011944	0.017099	0.032108	0.030611
15,24	**H9A15**	0.071125	0.069797	0.081014	0.064972	0.052496
15,57	**H3A15**	0.026414	0.016788	0.015651	0.016918	0.01305
16,44	**H4A15**	0.02266	0.039067	0.050161	0.042288	0.036733

Figure S8. Evaluation of the composition of protein-templated reaction DCL2 (exp in duplicate).

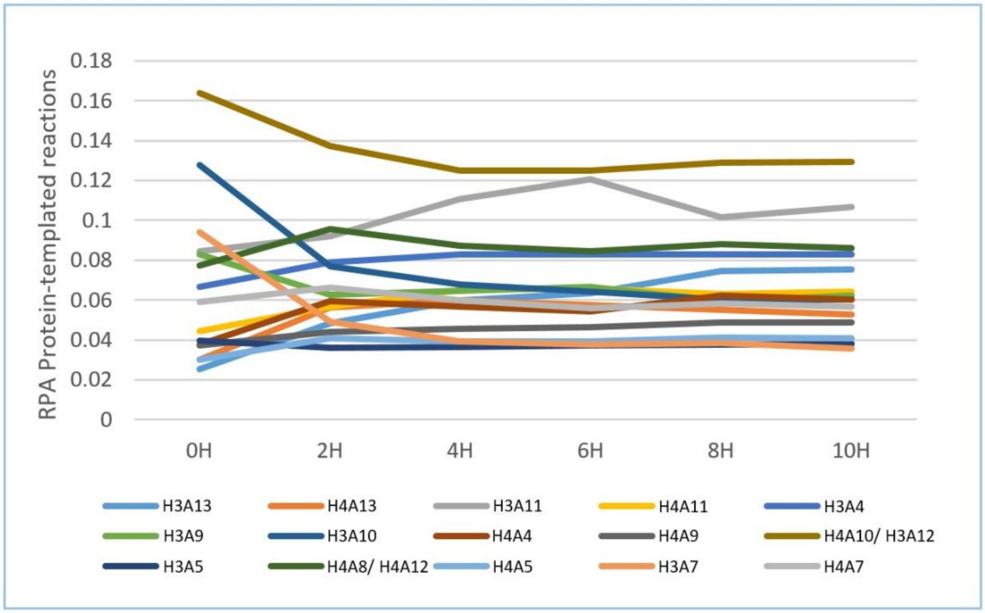

Table S10. RPAs values in protein-templated reaction DCL2 (exp in duplicate).

Apex RT (min)	Cpd	RPA 0H	RPA 2H	RPA 4H	RPA 6H	RPA 8H	RPA 10H
8.02	**H3A13**	0.0255	0.04852	0.059948	0.06333	0.07457	0.07552
8.51	**H4A13**	0.03013	0.0561	0.059994	0.0575	0.05505	0.05266
10.7	**H3A11**	0.08455	0.09211	0.110751	0.12052	0.10146	0.10674
11.5	**H4A11**	0.0442	0.05677	0.064864	0.06569	0.0631	0.06421
12.55	**H3A4**	0.06654	0.07889	0.082788	0.0827	0.08301	0.08278
12.77	**H3A9**	0.08288	0.06275	0.064791	0.06678	0.05926	0.06214
13.1	**H3A10**	0.12788	0.07681	0.067753	0.06405	0.06016	0.06029
13.41	**H4A4**	0.03777	0.05949	0.056665	0.05421	0.06208	0.0602
13.64	**H4A9**	0.03712	0.04381	0.045476	0.04637	0.04882	0.04884
13.95	**H4A10/ H3A12**	0.16382	0.13738	0.125045	0.12499	0.12887	0.12934
14.1	**H3A5**	0.03943	0.03589	0.036556	0.03712	0.03776	0.03797
14.74	**H4A8/ H4A12**	0.07712	0.09567	0.087121	0.08428	0.08819	0.08604
14.9	**H4A5**	0.0302	0.04062	0.03923	0.03912	0.04136	0.04093
15.45	**H3A7**	0.09383	0.04908	0.039311	0.03759	0.03824	0.03559
16.2	**H4A7**	0.05904	0.0661	0.059708	0.05576	0.05809	0.05675

Figure S9. Amplification factor % for DCL2.

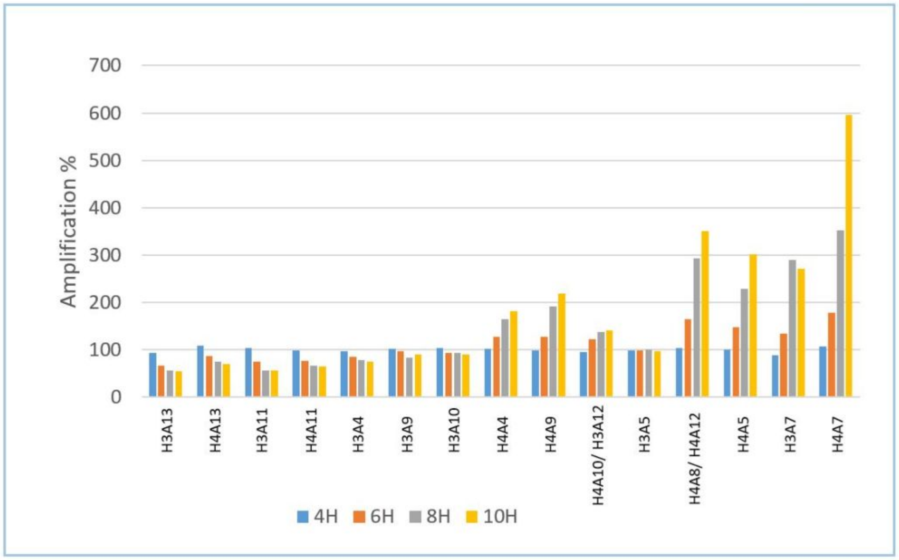

Table S11. Amplification factor % values for DCL2.

Apex RT (min)	Cpd	4H	6H	8H	10H
8.02	**H3A13**	93.40828	66.05468	55.74022	54.6077
8.51	**H4A13**	109.3241	86.02207	74.09403	70.17041
10.7	**H3A11**	104.0437	75.51552	56.07381	55.44626
11.5	**H4A11**	98.93228	76.06033	65.50303	64.17269
12.55	**H3A4**	97.43234	84.58051	77.50323	74.10961
12.77	**H3A9**	101.8668	96.78329	83.01393	90.05443
13.1	**H3A10**	103.4577	93.59827	93.86812	90.57175
13.41	**H4A4**	101.0936	127.6277	**164.4027**	**180.6518**
13.64	**H4A9**	98.49505	127.4541	**191.4205**	**218.8101**
13.95	**H4A10/ H3A12**	94.50636	122.3348	138.0821	141.5594
14.1	**H3A5**	98.65988	98.28589	100.0331	97.18371
14.74	**H4A8/ H4A12**	103.8414	164.4174	**293.468**	**350.7219**
14.9	**H4A5**	100.1887	147.0668	**229.2592**	**301.9638**
15.45	**H3A7**	87.80094	133.6795	**289.9078**	**271.2191**
16.2	**H4A7**	107.6665	177.8674	**352.7202**	**595.7116**

Figure S10. Normalized change of RPA for DCL2.

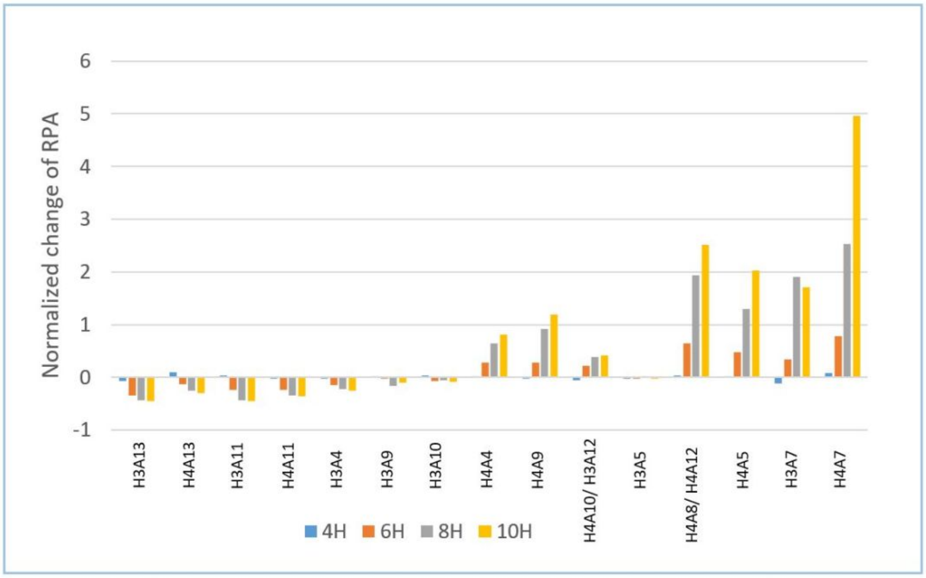

Table S12. Normalized change values of RPA for DCL2.

Apex RT (min)	Cpd	4H	6H	8H	10H
8.02	**H3A13**	-0.06592	-0.33945	-0.4426	-0.45392
8.51	**H4A13**	0.093241	-0.13978	-0.25906	-0.2983
10.7	**H3A11**	0.040437	-0.24484	-0.43926	-0.44554
11.5	**H4A11**	-0.01068	-0.2394	-0.34497	-0.35827
12.55	**H3A4**	-0.02568	-0.15419	-0.22497	-0.2589
12.77	**H3A9**	0.018668	-0.03217	-0.16986	-0.09946
13.1	**H3A10**	0.034577	-0.06402	-0.06132	-0.09428
13.41	**H4A4**	0.010936	0.276277	0.644027	0.806518
13.64	**H4A9**	-0.01505	0.274541	0.914205	1.188101
13.95	**H4A10/ H3A12**	-0.05494	0.223348	0.380821	0.415594
14.1	**H3A5**	-0.0134	-0.01714	0.000331	-0.02816
14.74	**H4A8/ H4A12**	0.038414	0.644174	1.93468	2.507219
14.9	**H4A5**	0.001887	0.470668	1.292592	2.019638
15.45	**H3A7**	-0.12199	0.336795	1.899078	1.712191
16.2	**H4A7**	0.076665	0.778674	2.527202	4.957116

Figure S11.Evaluation of the composition of protein-templated reaction DCL3(exp in duplicate).

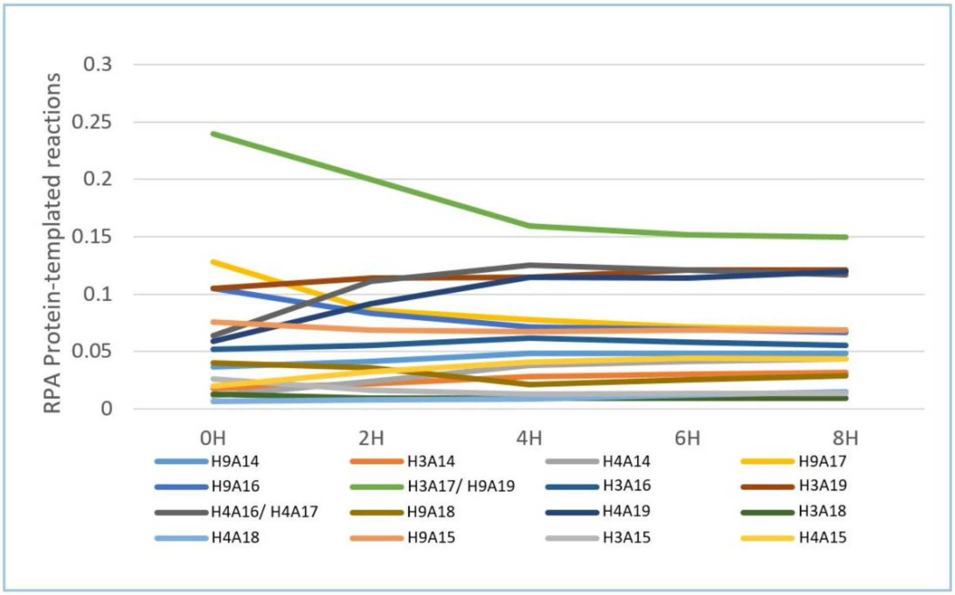

Table S13. RPAs values in protein-templated reaction DCL3 (exp in duplicate).

Apex RT (min)	Cpd	0H	2H	4H	6H	8H
9,04	**H9A14**	0.036332	0.041469	0.048482	0.048295	0.048636
9,86	**H3A14**	0.017669	0.022001	0.028221	0.030537	0.031538
10,44	**H4A14**	0.011414	0.023997	0.038172	0.041397	0.043285
12,02	**H9A17**	0.128163	0.086196	0.077762	0.071516	0.068467
12,12	**H9A16**	0.105229	0.083073	0.071091	0.069265	0.066775
12,42	**H3A17/ H9A19**	0.239819	0.199836	0.159259	0.15205	0.149385
12,52	**H3A16**	0.051862	0.055523	0.06158	0.058079	0.055148
13,03	**H3A19**	0.105101	0.114249	0.114442	0.120681	0.121071
13,30	**H4A16/ H4A17**	0.064107	0.111329	0.125584	0.120678	0.116795
13,67	**H9A18**	0.040215	0.035923	0.020959	0.025411	0.029174
13,95	**H4A19**	0.05922	0.091912	0.114998	0.114065	0.119592
14,04	**H3A18**	0.012853	0.009171	0.009327	0.009432	0.009351
15,14	**H4A18**	0.006251	0.0077	0.008822	0.012349	0.015155
15,24	**H9A15**	0.075497	0.068926	0.06738	0.068924	0.068945
15,57	**H3A15**	0.026177	0.016377	0.013092	0.013402	0.013414
16,44	**H4A15**	0.020091	0.032317	0.040829	0.043921	0.043271

Figure S12. Amplification factor % for DCL3.

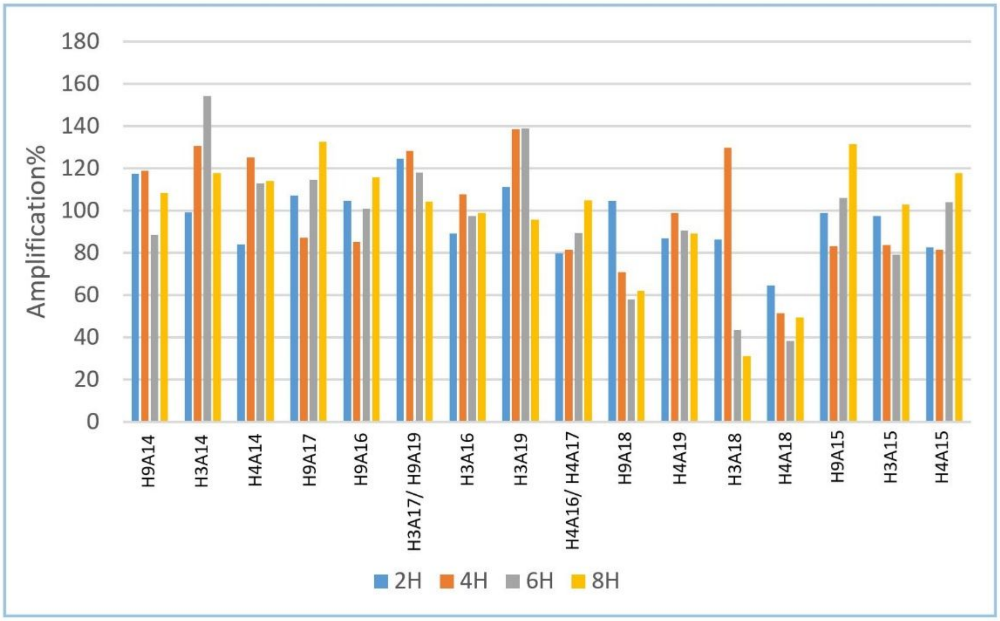

Table S14. Amplification factor % values for DCL3.

Apex RT (min)	Cpd	2H	4H	6H	8H
9,04	**H9A14**	117.3293	118.9679	88.69511	108.2655
9,86	**H3A14**	99.03982	130.6331	154.1247	117.7566
10,44	**H4A14**	84.05619	125.1063	112.9654	113.9663
12,02	**H9A17**	107.1359	87.24103	114.6378	132.5433
12,12	**H9A16**	104.4405	85.30242	100.7801	115.8442
12,42	**H3A17/ H9A19**	124.4595	128.4124	117.9951	104.3316
12,52	**H3A16**	89.1012	107.8445	97.44597	98.76008
13,03	**H3A19**	111.0115	138.5362	138.722	95.71182
13,30	**H4A16/ H4A17**	79.63219	81.52675	89.53987	104.8471
13,67	**H9A18**	104.4334	70.9393	58.1334	62.00902
13,95	**H4A19**	86.98518	98.94362	90.45785	89.1893
14,04	**H3A18**	86.18418	129.6659	43.56339	31.08845
15,14	**H4A18**	64.4697	51.59239	38.45896	49.50731
15,24	**H9A15**	98.7525	83.17129	106.0822	131.3335
15,57	**H3A15**	97.55039	83.65379	79.22244	102.7889
16,44	**H4A15**	82.72342	81.39614	103.8613	117.7993

Figure S13. Normalized change of RPA for DCL3.

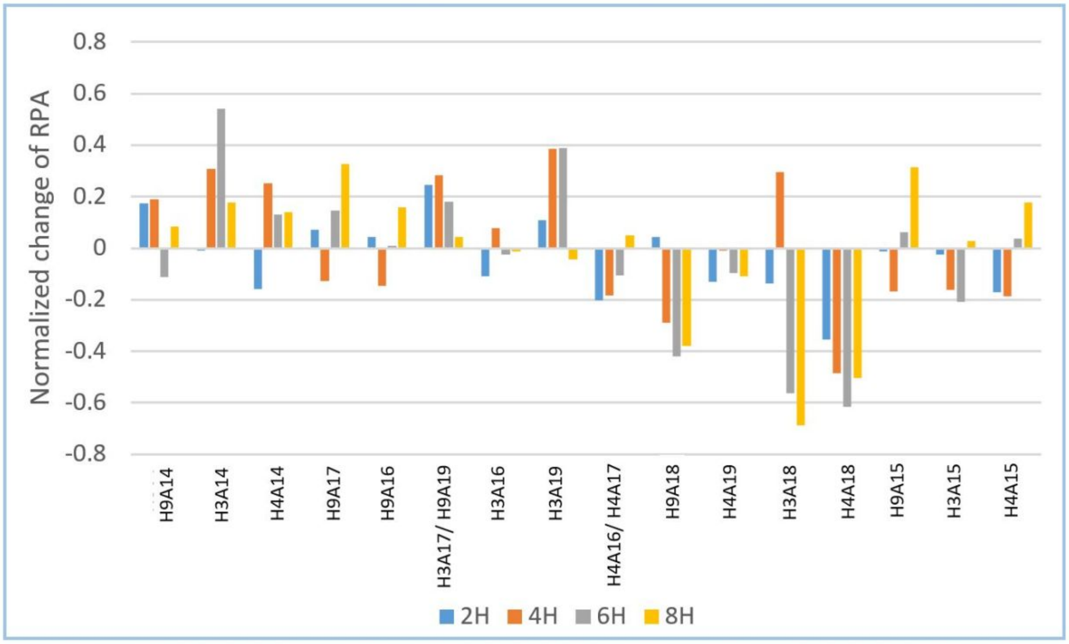

Table S15. Normalized change values of RPA for DCL3.

Apex RT (min)	Cpd	2H	4H	6H	8H
9,04	**H9A14**	0.173293	0.189679	-0.11305	0.082655
9,86	**H3A14**	-0.0096	0.306331	0.541247	0.177566
10,44	**H4A14**	-0.15944	0.251063	0.129654	0.139663
12,02	**H9A17**	0.071359	-0.12759	0.146378	0.325433
12,12	**H9A16**	0.044405	-0.14698	0.007801	0.158442
12,42	**H3A17/ H9A19**	0.244595	0.284124	0.179951	0.043316
12,52	**H3A16**	-0.10899	0.078445	-0.02554	-0.0124
13,03	**H3A19**	0.110115	0.385362	0.38722	-0.04288
13,30	**H4A16/ H4A17**	-0.20368	-0.18473	-0.1046	0.048471
13,67	**H9A18**	0.044334	-0.29061	-0.41867	-0.37991
13,95	**H4A19**	-0.13015	-0.01056	-0.09542	-0.10811
14,04	**H3A18**	-0.13816	0.296659	-0.56437	-0.68912
15,14	**H4A18**	-0.3553	-0.48408	-0.61541	-0.50493
15,24	**H9A15**	-0.01247	-0.16829	0.060822	0.313335
15,57	**H3A15**	-0.0245	-0.16346	-0.20778	0.027889
16,44	**H4A15**	-0.17277	-0.18604	0.038613	0.177993

3. Chemistry

Material and Methods.

Solvents and reagents were obtained from commercial suppliers and used without further purification. If required, solvents were distilled prior to use. NMR experiments were run on a Bruker Avance III 400 MHz spectrometer (400.13 MHz for ^{1}H and 100.62 MHz for ^{13}C), equipped with a BBI probe and Z-gradients, or on a Bruker FT NMR Avance III 600-MHz spectrometer (600.130 MHz for 1H and 150.903 MHz for 13C) equipped with a 5 mm CryoProbe QCI quadruple resonance, a shielded Z-gradient coil, and the automatic sample changer SampleJet NMR system. Spectra were acquired at 300 K, using deuterated dimethylsulfoxide (DMSO-d_6) as solvent. Chemical shifts for ^{1}H and ^{13}C spectra were recorded in parts per million using the residual non-deuterated solvent as the internal standard (for DMSO-d_6, ^{1}H 2.50 ppm, ^{13}C 39.52 ppm). The coupling constants of the splitting patterns were reported in Hz and were indicated as singlet (s), doublet (d), triplet (t), and multiplet (m). Due to the presence of isomers for acylhydrazones, some of the signals are doubled. UPLC−MS analyses were run on a Waters ACQUITY UPLC/MS system consisting of an SQD (single quadrupole detector) mass spectrometer equipped with an electrospray ionization interface and a photodiode array detector. The PDA range was 210−400 nm. The analyses were performed on either an ACQUITY UPLC HSS T3 C18 column (50 × 2.1 mm i.d., particle size 1.8 μm) with a VanGuard HSS T3 C18 precolumn (5 mm × 2.1 mm i.d., particle size 1.8 μm) (log D < 1) or an ACQUITY UPLC BEH C18 column (50 mm × 2.1 mm i.d., particle size 1.7 μm) with a VanGuard BEH C18 precolumn (5 mm × 2.1 mm i.d., particle size 1.7 μm) (log D > 1). The mobile phase was 10 mM NH_4OAc in H_2O at pH 5 adjusted with AcOH (A) and 10 mM NH_4OAc in MeCN−H_2O (95:5) at pH 5 (B). Electrospray ionization in positive and negative mode was applied in the mass scan range 100−500 Da. Methods and gradients used were the following: Generic method. Column: Waters ACQUITY UPLC BEH C18, 1.7 μm, 50 mm × 2.1 mm i.d. Linear gradient: 0−0.2 min, 5% B; 0.2−2.7 min, 5−95% B; 2.7−2.8 min, 95−100% B; 2.8−3.0 min, 100% B. Flow rate: 0.5 mL/min. Apolar method. Column: Waters ACQUITY UPLC BEH C18, 1.7 μm, 50 mm × 2.1 mm i.d. Precolumn: VanGuard BEH C18, 1.7 μm, 5 mm × 2.1 mm i.d. Gradient: 0−0.2 min, 50% B; 0.2− 2.7 min, 50−100% B; 2.7−3.0 min, 100% B. Flow rate: 0.5 mL/min. Compounds were named using the naming algorithm developed by CambridgeSoft Corporation and used in ChemBioDraw Ultra 16.0. All final compounds displayed ≥ 95% purity

as determined by UPLC/ MS analysis. No unexpected or unusually high safety hazards were encountered.

General Procedure for the Synthesis of *N*-acylhydrazone (1−16)

To a heat-dried Schlenk tube equipped with a magnetic stirring bar, the hydrazide (1.0 equiv.) and the corresponding aldehyde (1.0 equiv.) was dissolved/suspended in MeOH under nitrogen atmosphere. The reaction mixture was stirred at 65 °C until completion. The reaction was cooled to room temperature, and then the reaction mixture was precipitated by cooling at 0 °C in an ice bath. The precipitated reaction mixture was transferred to Eppendorf Tube® and centrifuged for 2 minutes, the supernatant liquid was removed and 1 mL ice-cold MeOH was added to the residue, which was re-suspended by vigorous agitation by a vortex mixer. The cold suspension was centrifuged again for 2 min, followed by removal of supernatant liquid. This process was repeated for at least three times or until the sufficiently pure product (residue) was not isolated, which was monitored on LC-MS. The solvents were removed under reduced pressure to obtained pure acylhydrazone product in 52–87 % yields.

***Tert*-butyl (*S*) - (1- (2- (4- (dimethylamino) benzylidene) hydrazineyl) - 1 - oxo -3-phenylpropan-2-yl) carbamate (1) (A1H3)**

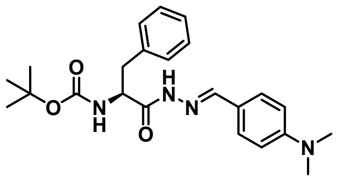

The acylhydrazone **1** (**A1H3)** was synthesized according to the general procedure, using N-(*tert*-butoxycarbonyl)-L-phenylalanine acid hydrazide **H3** (46 mg, 0.165 mmol) and 4-(dimethylamino) benzaldehyde **A1** (24.6 mg, 0.165 mmol) in MeOH (1.0 mL). After purification through cold MeOH washings, **1** (**A1H3)** was obtained as a mixture of *E* and *Z* isomers (*E*:*Z* = 47:53) as a white pure solid (46 mg, 70% yield). NMR spectra are reported as mixture of *E* and *Z* isomers. ^{1}H-NMR (500 MHz, DMSO-d_6) δ 11.22 (s, 1H, *E*), 11.07 (s, 1H, *Z*), 8.04 (s, 1H, *E*), 7.88 (s, 1H, *Z*), 7.51 (t, J = 8.5 Hz, 4H), 7.37 – 7.24 (m, 8H), 7.20 (m, 2H), 7.13 (d, J = 8.3 Hz, 1H), 6.99 (d, J = 8.9 Hz, 1H), 6.76 (dd, J = 12.9, 9.0 Hz, 4H), 5.03 (td, J = 10.2, 3.6 Hz, 1H), 4.19 (td, J = 9.5, 5.0 Hz, 1H), 2.98 (s, 6H), 2.97 (s, 6H), 2.94 (m, 2H), 2.79 (m, 2H), 1.32 (s, 9H), 1.31 (s, 9H). ^{13}C-NMR (126 MHz, DMSO-d_6) δ 173.1, 168.2, 155.9, 155.8, 151.9, 151.8, 148.0, 144.7, 139.0, 138.5, 129.7, 129.5, 128.8, 128.5, 128.5, 128.4, 126.7, 122.0, 121.9, 112.3, 112.2, 78.5, 78.3, 55.5, 53.4, 37.8, 36.8, 28.63; ESI-MS for $C_{23}H_{30}N_4O_3$: calcd 410.2, found *m/z* 411.2 $[M+H]^+$. UPLC-MS generic method: t_R = 6.08 min, @215nm 98.1%, @254nm 98.5% purity.

N'-((1*H*-indol-6-yl) methylene)-4-(2,4-dichlorophenoxy)butanehydrazide (2) (**A2H4**)

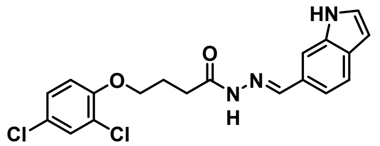

The acylhydrazone **2** (**A2H4**) was synthesized according to the general procedure, using 4-(2,4-dichlorophenoxy)butanehydrazide **H4** (51.6 mg, 0.196 mmol) and 1*H*-indole-6-carbaldehyde **A2** (28.5 mg, 0.196 mmol) in MeOH (1.0 mL). After purification through cold MeOH washings, **2 (A2H4)** was obtained as a mixture of *E* and *Z* isomers (*E*:*Z* = 40: 60) as a white-pink pure solid (51 mg, 67% yield). NMR spectra are reported as mixture of isomers, in agreement with R.P. Jumde *et al.*[4] ^{1}H-NMR (500 MHz, DMSO-d_6) δ 11.29 (br s, 1H, *E*), 11.27 (s, 1H, *Z*), 11.25 (br s, 1H, *E*), 11.15 (s, 1H, *Z*), 8.22 (s,1H), 8.06 (s, 1H), 7.69 (s, 1H), 7.61 (s, 1H), 7.59 – 7.51 (m, 4H), 7.46 (t, J = 2.8 Hz, 1H), 7.44 (t, J = 2.7 Hz, 1H), 7.41– 7.32 (m, 4H), 7.20 (dd, J = 9.0, 2.3 Hz, 2H), 6.46 (q, J =2.4 Hz, 2H), 4.16 (t, J = 6.4 Hz, 2H), 4.12 (t, J = 6.3 Hz, 2H), 2.85 (t, J = 7.4 Hz, 2H), 2.42 (t, J= 7.3 Hz, 2H), 2.14 – 2.00 (m, 4H). ^{13}C NMR (126 MHz, DMSO-d_6) δ 173.9, 168.1, 153.5, 148.0, 144.9, 136.3, 136.22, 129.7, 129.5, 128.6, 127.9, 127.8, 124.8, 124.8, 122.9, 120.8, 120.7, 118.4, 117.6, 115.5, 115.47, 111.49, 111.28, 102.0, 102.0, 68.8, 30.8, 28.8, 24.8, 24.1 ESI-MS for $C_{19}H_{17}Cl_2N_3O_2$: calcd 389.0, found *m/z* 390.0 $[M+H]^+$. UPLC-MS generic method: t_R = 4.97 min @215, 98.1%, @254nm 98 % purity.

N'-((1*H*-indol-6-yl)methylene)-3-(*tert*-butyl)-1-(3-methylbenzyl)-1*H*-pyrazole-5-carbohydrazide (3) (A2H7)

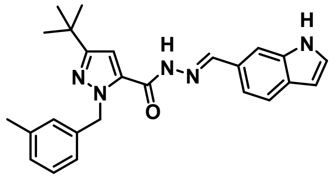

The acylhydrazone **3** (**A2H7**) was synthesized according to the general procedure, using 3-(*tert*-butyl)-1-(3-methylbenzyl)-1*H*-pyrazole-5-carbohydrazide **H7** (73.9 mg, 0.279 mmol) and 1*H*-indole-6-carbaldehyde **A2** (37.4 mg, 0.279 mmol) in MeOH (1.0 mL). After purification through cold MeOH washings, **3** (**A2H7**) was obtained as a yellow-orange pure solid (93 mg, 81% yield). ^{1}H-NMR (500 MHz, DMSO-d_6) δ 11.64 (s, 1H), 11.32 (s, 1H), 8.43 (s, 1H), 7.71 (s, 1H), 7.59 (d, J = 8.2 Hz, 1H), 7.47 (s, 1H), 7.39 (d, J = 8.3 Hz, 1H), 7.18 (t, J = 7.6 Hz, 1H), 7.05 (d, J = 7.5 Hz, 1H), 6.95 (d, J = 27.9 Hz, 2H), 6.90 (d, J = 7.6 Hz, 1H), 6.48 (d, J = 2.2 Hz, 1H), 5.68 (s, 2H), 2.25 (s, 3H), 1.30 (s, 9H); ^{13}C-NMR (126 MHz, DMSO-d_6) δ 193.2, 160.0, 156.1, 150.2, 138.6, 137.8, 136.2, 134.5, 129.9, 128.7, 128.3, 128.1, 128.1, 127.5, 124.5, 120.8, 118.5, 111.5, 104.7, 102.0, 53.9, 32.2, 30.8, 21.5; ESI-MS for $C_{25}H_{27}N_5O$: calcd 413.2, found *m/z* 414.2 $[M+H]^+$; 412.2 $[M-H]^-$. UPLC-MS generic method: t_R = 5.35 min, @215nm 97.9%, @254nm 96% purity.

3-(*tert*-butyl)-N'-(4-(dimethylamino) benzylidene)-1-(3-methylbenzyl)-1*H*-pyrazole-5-carbohydrazide (4) (**A1H7**)

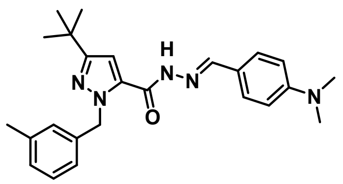

The acylhydrazone **4** (**A1H7**) was synthesized according to the general procedure, using 3-(*tert*-butyl)-1-(3-methylbenzyl)-1*H*-pyrazole-5-carbohydrazide **H7** (62.5 mg, 0.218 mmol) and 4-(dimethylamino) benzaldehyde **A1** (32.6 mg, 0.218 mmol) in MeOH (1.0 mL). After purification through cold MeOH washings, **4** (**A1H7**) was obtained as a white pure solid (59 mg, 65% yield). ^{1}H-NMR (500 MHz, DMSO-d_6) δ 11.49 (s, 1H), 8.23 (s, 1H), 7.51 (d, J = 8.9 Hz, 2H), 7.18 (t, J = 7.6 Hz, 1H), 7.05 (d, J = 7.5 Hz, 1H), 6.97 (s, 1H), 6.91 – 6.83 (m, 2H), 6.76 (d, J = 8.9 Hz, 2H), 5.66 (s, 2H), 2.98 (s, 6H), 2.25 (s, 3H), 1.29 (s, 9H); ^{13}C-NMR (126 MHz, DMSO-d_6) δ 160.0, 155.9, 152.0, 149.2, 138.6, 137.8, 134.6, 128.9, 128.7, 128.3, 128.0, 124.4, 121.7, 112.2, 104.5, 53.9, 32.2, 30.8, 21.5; ESI-MS for $C_{25}H_{31}N_5O$: calcd 417.2, found *m/z* 418.2 $[M+H]^+$. UPLC-MS generic method: t_R = 5.68 min, @215nm 99.9%, @254nm 99.5% purity.

4-(2,4-dichlorophenoxy)-N'-((4,5,6,7-tetrahydrobenzo[b]thiophen-2-yl) methylene) butanehydrazide (5) (A3H4)

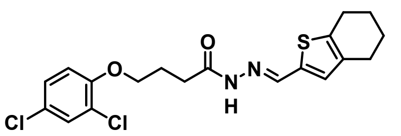

The acylhydrazone **5** (**A3H4)** was synthesized according to the general procedure, using 4-(2,4-dichlorophenoxy)butanehydrazide **H4** (91.8 mg, 0.349 mmol) and 4,5,6,7-tetrahydrobenzo[b]thiophene-2-carbaldehyde **A3** (58.0 mg, 0.349 mmol) in MeOH (1.0 mL). After purification through cold MeOH washings, **5** (**A3H4**) was obtained as a mixture of *E* and *Z* isomers (*E*:*Z* = 43:57) as a yellowish pure solid (92 mg, 65% yield). NMR spectra are reported as mixture of isomers. ^{1}H-NMR (500 MHz, DMSO-d_6) δ 11.25 (s, 1H), 11.15 (s, 1H), 8.26 (s, 1H), 8.03 (s, 1H), 7.56 (t, J = 2.2 Hz, 2H), 7.39 – 7.30 (m, 2H), 7.18 (d, J = 8.8 Hz, 2H), 7.07 (s, 1H), 7.01 (s, 1H), 4.10 (dt, J = 12.8, 6.2 Hz, 4H), 2.70 (dt, J = 12.6, 6.5 Hz, 8H), 2.36 (t, J = 7.2 Hz, 2H), 2.01 (dd, J = 12.4, 6.2 Hz, 2H), 1.85 – 1.64 (m, 8H). ^{13}C-NMR (126 MHz, DMSO-d_6) δ 173.7, 168.1, 153.4, 141.8, 138.7, 138.4, 138.1, 136.0, 135.6, 131.9, 131.1, 129.7, 128.6, 128.5, 124.8, 124.7, 122.9, 122.8, 115.5, 115.5, 68.8, 30.8, 28.6, 25.3, 25.2, 25.1, 24.7, 24.0, 23.3, 22.6; ESI-MS for $C_{19}H_{20}Cl_2N_2O_2S$: calcd 410.0, found *m/z* 411.0 $[M+H]^+$. UPLC-MS generic method: t_R =4.84 min, @215nm 99.3%, @254nm 99 % purity.

3-(*tert*-butyl)-1-(3-methylbenzyl)-N'-((4,5,6,7-tetrahydrobenzo[b]thiophen-2-yl)methylene)-1*H*-pyrazole-5-carbohydrazide (6) (A3H7)

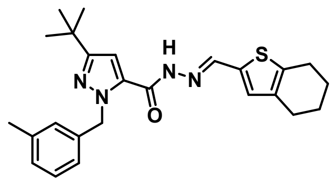

The acylhydrazone **6** (**A3H7**) was synthesized according to the general procedure, using 3-(*tert*-butyl)-1-(3-methylbenzyl)-1*H*-pyrazole-5-carbohydrazide **H7** (51.9 mg, 0.180 mmol) and 4,5,6,7-tetrahydrobenzo[b]thiophene-2-carbaldehyde **A3** (30.1 mg, 0.180 mmol) in MeOH (1.0 mL). After purification through cold MeOH washings, **6** (**A3H7**) was obtained as a yellow pure solid (56 mg, 72% yield). ^{1}H-NMR (500 MHz, DMSO-d_6) δ 11.64 (s, 1H), 8.46 (s, 1H), 7.17 (s, 1H), 7.13 (s, 1H), 7.05 (d, J = 7.5 Hz, 1H), 6.96 (s, 1H), 6.88 (s, 2H), 5.64 (s, 2H), 2.73 (t, J = 5.6 Hz, 2H), 2.55 (t, J = 5.8 Hz, 2H), 2.25 (s, 3H), 1.75 (ddd, J = 14.8, 9.0, 4.4 Hz, 4H), 1.28 (d, J = 7.3 Hz, 9H); ^{13}C-NMR (126 MHz, DMSO-d_6) δ 160.0, 155.9, 143.7, 139.4, 138.6, 137.8, 136.2, 135.3, 134.3, 132.6, 128.7, 128.3, 128.0, 124.4, 104.7, 53.9, 32.2, 30.8, 25.2, 23.3, 22.6, 21.5; ESI-MS for $C_{25}H_{30}N_4OS$: calcd 434.2, found *m/z* 435.2 $[M+H]^+$. UPLC-MS apolar method: t_R = 4.24 min, @215nm 98.8%, @254nm 98 % purity.

4-(2,4-dichlorophenoxy)-*N'*-((2-fluoropyridin-3-yl)methylene)butanehydrazide (7) (H1A1)

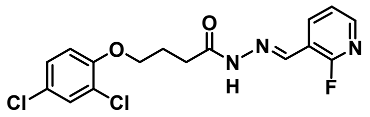

The acylhydrazone **7** (**H1A1**) was synthesized according to the general procedure, using 4-(2,4-dichlorophenoxy)butanehydrazide **H1** (435 mg, 1.65 mmol) and 2-fluoronicotinaldehyde **A1** (207 mg, 1.65 mmol) in MeOH (1.5 mL). After purification through cold MeOH washings, **7** (**H1A1**) was obtained as a mixture of *E* and *Z* isomers (*E*:*Z* = 30:70) as white-yellow pure solid (430 mg, 70% yield). ^{1}H-NMR (500 MHz, DMSO-d_6) δ 11.54 (s, 2H), 8.30 (dt, J = 17.4, 6.7 Hz, 3H), 8.08 (s, 1H), 7.53 (d, J = 4.2 Hz, 2H), 7.48 – 7.37 (m, 2H), 7.34 (dd, J = 13.8, 5.6 Hz, 2H), 7.17 (dd, J = 8.8, 4.8 Hz, 2H), 4.12 (dd, J = 10.9, 4.9 Hz, 4H), 2.83 (t, J = 7.3 Hz, 2H), 2.43 (t, J = 7.3 Hz, 2H), 2.12 – 1.97 (m, 4H). ^{13}C-NMR (126 MHz, DMSO-d_6) δ 174.0, 161.1, 152.9, 148.1, 137.2, 136.6, 134.1, 129.3, 128.2, 124.2, 122.2, 115.0, 68.3, 28.3, 23.7. ESI-MS for $C_{16}H_{14}Cl_2FN_3O_2$: calcd 369.0, found *m/z* 370.0/372.0/374.1 $[M+H]^+$, 368.1/370.1/372.1 $[M-H]^-$. UPLC-MS generic method: t_R = 4.66 min, @215nm 99.9%, @254nm 99.5% purity.

4-(2,4-dichlorophenoxy)-*N'*-((6-methoxypyridin-3-yl)methylene)butanehydrazide (8) (H1A6)

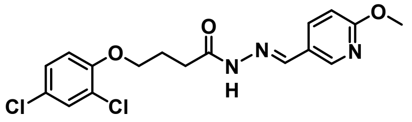

The acylhydrazone **8** (**H1A6**) was synthesized according to the general procedure, using 4-(2,4-dichlorophenoxy) butanehydrazide **H1** (126 mg, 0.48 mmol) and 6-methoxynicotinaldehyde **A6** (66 mg, 0.48 mmol) in MeOH (1.5 mL). After purification through cold MeOH washings, **8** (**H1A6**) was obtained as a mixture of *E* and *Z* isomers (*E*:*Z* = 38:62) as a white-yellow pure solid (109 mg, 60% yield). ^{1}H-NMR (500 MHz, DMSO-d_6) δ 11.40 (s, 1H), 11.29 (s, 1H), 8.37 (d, J = 2.0 Hz, 1H), 8.33 (d, J = 2.1 Hz, 1H), 8.16 (s, 1H), 8.04 (dd, J = 8.7, 2.2 Hz, 1H), 8.00 (dd, J = 8.7, 2.3 Hz, 1H), 7.95 (s, 1H), 7.56 (t, J = 2.8 Hz, 2H), 7.35 (td, J = 8.7, 2.6 Hz, 2H), 7.19 (dd, J = 9.0, 2.8 Hz, 2H), 6.90 (d, J = 8.7 Hz, 1H), 6.86 (d, J = 8.7 Hz, 1H), 4.12 (dt, J = 12.9, 6.4 Hz, 4H), 2.81 (t, J = 7.4 Hz), 2.41 (t, J = 7.3 Hz), 2.04 (p, J = 6.8 Hz, 4H). ^{13}C-NMR (126 MHz, DMSO-d_6) δ 173.5, 167.6, 164.0, 152.7, 146.6, 143.0, 139.6, 135.4, 129.0, 127.9, 124.1, 122.2, 114.8, 110.9, 68.1, 53.2, 29.9, 28.2, 23.5. ESI-MS for $C_{17}H_{17}Cl_2N_3O_3$: calcd 381.0, found *m/z* 382.0/384.0/386.0 $[M+H]^+$, 380.1/382.1/384.1 $[M-H]^-$. UPLC-MS generic method: t_R = 4.81 min, @215nm 95.4% purity.

4-(2,4-dichlorophenoxy)-*N'*-(3-methoxybenzylidene)butanehydrazide (**9**) (**H1A9**)

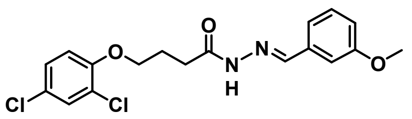

The acylhydrazone **9** (**H1A9**) was synthesized according to the general procedure, using 4-(2,4-dichlorophenoxy) butanehydrazide **H1** (99 mg, 0.376 mmol) and 3-methoxybenzaldehyde **A9** (45.8 µL, 0.376 mmol) in MeOH (0.76 mL). After purification through cold MeOH washings, **9** (**H1A9**) was obtained as a mixture of *E* and *Z* isomers (*E*:*Z* = 37:63) as a white-pink pure solid (104 mg, yield 73%). NMR spectra are reported as mixture of isomers. ^{1}H-NMR (400 MHz, DMSO-d_6) δ 11.43 (s, 1H), 11.31 (s, 1H), 8.13 (s, 1H), 7.94 (s, 1H), 7.55 (dd, J = 4.9, 2.5 Hz, 2H), 7.39 – 7.28 (m, 4H), 7.26 – 7.14 (m, 6H), 7.01 – 6.94 (m, 2H), 4.19 – 4.06 (m, 4H), 3.78 (d, J = 6.4 Hz, 6H), 2.83 (t, J = 7.3 Hz, 2H), 2.42 (t, J = 7.3 Hz, 2H), 2.12 – 1.99 (m, 4H). ^{13}C-NMR (101 MHz, DMSO-d_6) δ 174.2, 171.0, 168.4, 159.9, 153.4, 146.1, 142.8, 136.2, 136.1, 130.2, 129.6, 128.54, 128.51, 124.8, 124.7, 122.8, 120.2, 119.5, 116.4, 115.8, 115.5, 115.4, 111.9, 111.5, 68.8, 68.7, 55.58, 55.54, 30.7, 28.7, 24.7, 24.1; ESI-MS for $C_{18}H_{18}Cl_2N_2O_3$: calcd

380.0, found *m/z* 381.2, 383.2 [M+H]$^+$. UPLC-MS generic method: t_R = 5.21 min, @215nm 96.7% purity.

4-(2,4-dichlorophenoxy)-*N'*-((6-(trifluoromethyl)pyridin-3-yl) methylene) butanehydrazide (10) (H1A2)

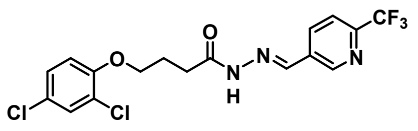

The acylhydrazone **10** (**H1A2**) was synthesized according to the general procedure, using 4-(2,4-dichlorophenoxy) butanehydrazide **H1** (100 mg, 0.38 mmol) and 6-(trifluoromethyl) nicotinaldehyde **A2** (66.5 mg, 0.38 mmol) in MeOH (0.76 mL). After purification through cold MeOH washings, **10** (**H1A2**) was obtained as a mixture of *E* and *Z* isomers (*E*:*Z* = 40:60) as a white-off pure solid (95 mg, yield 60%). NMR spectra are reported as mixture of isomers. ^{1}H-NMR (400 MHz, DMSO-d_6) δ 11.76 (s, 1H), 11.66 (s, 1H), 8.99 (s, 1H), 8.32 (dd, J = 23.2, 8.4 Hz, 3H), 8.08 (s, 1H), 7.96 (d, J = 8.2 Hz, 1H), 7.92 (d, J = 8.2 Hz, 1H), 7.59 – 7.51 (m, 2H), 7.35 (ddd, J = 11.4, 6.7, 2.6 Hz, 2H), 7.18 (dt, J = 8.8, 6.0 Hz, 2H), 4.12 (ddd, J = 18.4, 12.4, 6.3 Hz, 4H), 2.87 (t, J = 7.3 Hz, 2H), 2.46 (d, J = 7.3 Hz, 2H), 2.06 (t, J = 6.8 Hz, 4H). ^{13}C-NMR (101 MHz, DMSO-d_6) δ 174.6, 171.0, 168.9, 153.3, 149.1, 148.8, 146.8, 146.5, 141.9, 138.6, 135.8, 135.4, 134.1, 133.9, 129.6, 128.5, 128.4, 126.0, 124.8, 124.7, 123.3, 122.8, 121.3, 121.2, 120.6, 115.5, 115.46, 115.41, 68.7, 68.5, 30.8, 28.8, 24.5, 24.1; ESI-MS for $C_{17}H_{14}Cl_2F_3N_3O_2$: calcd 419.0, found *m/z* 420.2, 422.2 [M + H]$^+$. UPLC-MS generic method: t_R = 5.22 min, @215nm 96%, @254nm 99.5% purity.

***tert*-butyl (*S*)-(1-oxo-3-phenyl-1-(2-(3-(trifluoromethyl)benzylidene) hydrazineyl) propan-2-yl) carbamate (11) (H2A4)**

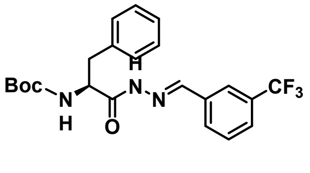

The acylhydrazone **11** (**H2A4**) was synthesized according to the general procedure, using *tert*-butyl (*S*)-(1-hydrazineyl-1-oxo-3-phenylpropan-2-yl) carbamate **H1** (99 mg, 0.354 mmol) and 3-trifluoromethylbenzaldehyde **A2** (47.0 µL, 0.354 mmol) in MeOH (0.75 mL). After purification through cold MeOH washings, **11** (**H2A4**) was obtained as a mixture of *E* and *Z* isomers (*E*:*Z* = 47:53) as a white pure solid (80 mg, yield 52%). NMR spectra are reported as mixture of isomers. ^{1}H-NMR (400 MHz, DMSO-d_6) δ 11.75 (s, 1H), 11.54 (s, 1H), 8.29 (s, 1H), 8.13 – 7.89 (m, 5H), 7.82 – 7.64 (m, 4H), 7.36 – 7.13 (m, 12H), 5.05 (s, 1H), 4.24 (s, 1H), 3.03 – 2.91 (m, 2H), 2.81 (dd, J = 26.2, 15.8 Hz, 2H), 1.31 (d, J = 3.3 Hz, 18H). ^{13}C-NMR (101 MHz,

DMSO-d_6) δ 173.8, 169.2, 155.9, 145.5, 142.0, 138.8, 138.2, 135.8, 135.7, 131.6, 131.4, 130.5, 130.4, 129.9, 129.6, 129.4, 128.5, 128.4, 126.7, 126.4, 125.8, 123.4, 122.6, 78.5, 78.4, 55.5, 53.5, 37.5, 36.8, 28.5, 20.8; ESI-MS for $C_{22}H_{24}F_3N_3O_3$: calcd 435.1, found *m/z* 436.1 $[M + H]^+$. UPLC-MS generic method: t_R = 5.26 min, @215nm 99.9%, @254nm 96% purity.

4-(2,4-dichlorophenoxy)-N'-(3-(trifluoromethyl)benzylidene)butanehydrazide (12) (**H1A4**)

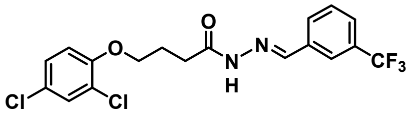

The acylhydrazone **12** (**H1A4**) was synthesized according to the general procedure, using 4-(2,4-dichlorophenoxy) butanehydrazide **H1** (99 mg, 0.376 mmol) and 3-trifluoromethylbenzaldehyde **A4** (50.3 µL, 0.376 mmol) in MeOH (0.76 mL). After purification through cold MeOH washings, **12** (**H1A4**) was obtained as a mixture of *E* and *Z* isomers (*E*:*Z* = 39:61) as a white pure solid (110 mg, yield 70%). NMR spectra are reported as mixture of isomers. ^{1}H-NMR (400 MHz, DMSO-d_6) δ 11.60 (s, 1H), 11.47 (s, 1H), 8.24 (s, 1H), 8.10 – 7.90 (m, 5H), 7.76 (t, J = 7.9 Hz, 2H), 7.66 (dt, J = 15.5, 7.8 Hz, 2H), 7.55 (dd, J = 9.2, 2.2 Hz, 2H), 7.34 (dd, J = 14.2, 5.5 Hz, 2H), 7.23 – 7.14 (m, 2H), 4.21 – 4.03 (m, 4H), 2.86 (t, J = 7.2 Hz, 2H), 2.44 (t, J = 7.2 Hz, 2H), 2.06 (dd, J = 13.4, 6.7 Hz, 4H). ^{13}C-NMR (101 MHz, DMSO-d_6) δ 174.4, 168.7, 153.3, 144.4, 141.4, 136.0, 135.8, 131.2, 130.8, 130.38, 130.33, 130.2, 129.8, 129.66, 129.63, 128.54, 128.50, 126.3, 124.8, 124.7, 123.2, 122.8, 115.5, 115.4, 68.7, 30.7, 28.6, 24.6, 24.0; ESI-MS for $C_{18}H_{15}Cl_2F_3N_2O_2$: calcd 418.0, found *m/z* 419.2, 421.2 $[M + H]^+$. UPLC-MS generic method: t_R = 5.76 min, @215nm 96%, @254nm 99.5% purity.

N'-(quinoxalin-6-ylmethylene)-4,5,6,7-tetrahydrobenzo[b]thiophene-2-carbohydrazide (**13**) (**H3A4**)

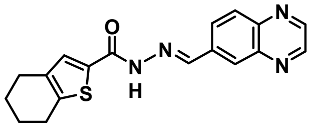

The acylhydrazone **13** (**H3A4**) was synthesized according to the general procedure, using 4,5,6,7-tetrahydrobenzo[b]thiophene-2-carbohydrazide **H3** (99 mg, 0.504 mmol) and quinoxaline-6-carbaldehyde **A4** (79.7 mg, 0.504 mmol) in MeOH (1.0 mL). After purification through cold MeOH washings, **13** (**H3A4**) was obtained as a mixture of *E* and *Z* isomers (*E*:*Z* = 50:50) as a white-yellowish pure solid (147 mg, yield 87%). NMR spectra are reported as mixture of isomers. ^{1}H-NMR (400 MHz, DMSO-d_6) δ 11.97 (s, 2H), 8.98 (dd, J = 12.2, 1.7 Hz, 4H), 8.64 (s, 1H), 8.31 (s, 5H), 8.18 (s, 2H), 7.71 (d, J = 29.6 Hz, 2H), 2.80 (s, 4H), 2.63 (t, J = 5.4 Hz, 4H), 1.87 – 1.68 (m, 8H). ^{13}C-NMR (101 MHz, DMSO-d_6) δ 146.8, 146.4, 143.4, 142.9, 136.4, 130.8, 130.3, 129.3,

127.4, 25.0, 23.2, 22.6; ESI-MS for $C_{18}H_{16}N_4OS$: calcd 336.1, found *m/z* 337.2 $[M + H]^+$. UPLC-MS generic method: t_R = 3.98 min, @215nm 99.9%, @254nm 99.5% purity.

N'-((1*H*-indazol-6-yl)methylene)-4,5,6,7-tetrahydrobenzo[b]thiophene-2-carbohydrazide (14) (H3A3)

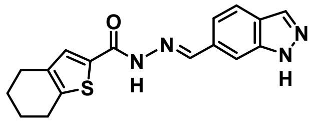

The acylhydrazone **14** (**H3A3)** was synthesized according to the general procedure, using 4,5,6,7-tetrahydrobenzo[b]thiophene-2-carbohydrazide **H3** (93 mg, 0.473 mmol) and 1*H*-indazole-6-carbaldehyde **A3** (69.3 mg, 0.473 mmol) in MeOH (0.95 mL). After purification through cold MeOH washings, **14** (**H3A3**) was obtained as a mixture of *E* and *Z* isomers (*E*:*Z* = 50:50) as a white-off pure solid (131 mg, yield 86%). NMR spectra are reported as mixture of isomers. ^{1}H-NMR (400 MHz, DMSO-d_6) δ 13.224 (s, 2H), 11.74 (s, 1H), 11.67 (s, 1H), 8.48 (s, 1H), 8.18 (s, 1H), 8.08 (s, 2H), 7.77 (d, J = 11.4 Hz, 4H), 7.61 (t, J = 31.9 Hz, 4H), 2.74 (s, 4H), 2.58 (s, 4H), 1.84 – 1.62 (m, 8H). ^{13}C-NMR (101 MHz, DMSO-d_6) δ 161.8, 158.2, 147.9, 144.8, 142.1, 140.3, 135.7, 134.1, 132.6, 129.9, 124.0, 121.4, 118.8, 110.2, 25.0, 23.2, 22.6; ESI-MS for $C_{17}H_{16}N_4OS$: calcd 324.1, found *m/z* 325.2 $[M + H]^+$. UPLC-MS generic method: t_R = 3.95 min, @215nm 99.9%, @254nm 99.5% purity.

N'-((1H-indazol-6-yl)methylene)-4-(2,4-dichlorophenoxy)butanehydrazide (15) (H1A3)

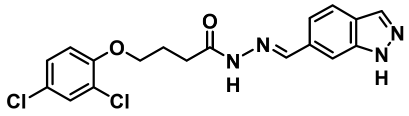

The acylhydrazone **15** (**H1A3**) was synthesized according to the general procedure, using 4-(2,4-dichlorophenoxy)butanehydrazide **H1** (99 mg, 0.376 mmol) and 1*H*-indazole-6-carbaldehyde **A3** (55.0 mg, 0.376 mmol) in MeOH (0.75 mL). After purification through cold MeOH washings, **15** (**H1A3**) was obtained as a mixture of *E* and *Z* isomers (*E*:*Z* = 36:64) as a white-off pure solid (98 mg, yield 67%). NMR spectra are reported as mixture of isomers. ^{1}H-NMR (400 MHz, DMSO-d_6) δ 13.21 (s, 1H), 13.18 (s, 1H), 11.44 (s, 1H), 11.32 (s, 1H), 8.27 (s, 1H), 8.09 (d, J = 4.0 Hz, 3H), 7.82 – 7.67 (m, 4H), 7.56 (d, J = 2.6 Hz, 2H), 7.51 (d, J = 8.5 Hz, 2H), 7.40 – 7.30 (m, 2H), 7.20 (d, J = 8.9 Hz, 2H), 4.13 (dt, J = 22.3, 6.4 Hz, 4H), 2.86 (t, J = 7.4 Hz, 2H), 2.43 (t, J = 7.3 Hz, 2H), 2.13 – 2.01 (m, 4H). ^{13}C-NMR (101 MHz, DMSO-d_6) δ 173.5, 167.7, 152.7, 146.1, 143.0, 139.6, 133.4, 132.1, 132.0, 129.0, 127.8, 124.1, 124.0, 123.2, 122.0, 120.6, 118.2, 117.6, 114.89, 114.83, 109.5, 68.1, 30.17, 28.1, 24.0, 23.4; ESI-MS for

$C_{18}H_{16}Cl_2N_4O_2$: calcd 390.1, found m/z 391.2, 393.3 $[M + H]^+$. UPLC-MS generic method: t_R = 4.39 min @215nm 97.3%, @254nm 99.5% purity.

4-(2,4-dichlorophenoxy)-*N'*-(quinoxalin-6-ylmethylene)butanehydrazide (16) (H1A4)

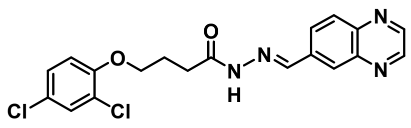

The acylhydrazone **16** (**H1A4**) was synthesized according to the general procedure, using 4-(2,4-dichlorophenoxy)butanehydrazide **H1** (108 mg, 0.41 mmol) and quinoxaline-6-carbaldehyde **A4** (64.9 mg, 0.41 mmol) in MeOH (0.82 mL). After purification through cold MeOH washings, **16** (**H1A4**) was obtained as a mixture of *E* and *Z* isomers (*E*:*Z* =35:65) as a white-pink pure solid (122 mg, yield 74%). NMR spectra are reported as mixture of isomers. ^{1}H-NMR (400 MHz, DMSO-d_6) δ 11.70 (s, 1H), 11.56 (s, 1H), 9.00 – 8.92 (m, 4H), 8.41 (s, 1H), 8.28 (s, 1H), 8.23 (dd, J = 9.6, 5.8 Hz, 4H), 8.13 (d, J = 8.7 Hz, 1H), 8.08 (d, J = 9.4 Hz, 1H), 7.56 (t, J = 3.1 Hz, 2H), 7.40 – 7.31 (m, 2H), 7.20 (d, J = 8.9 Hz, 2H), 4.15 (dt, J = 17.9, 6.2 Hz, 4H), 2.91 (t, J = 7.3 Hz, 2H), 2.46 (d, J = 7.4 Hz, 2H), 2.15 – 2.03 (m, 4H). ^{13}C-NMR (101 MHz, DMSO-d_6) δ 174.5, 153.4, 146.7, 146.3, 143.3, 142.9, 141.7, 136.5, 130.0, 129.6, 129.2, 128.8, 128.5, 127.0, 124.7, 122.8, 115.4, 68.7, 30.8, 28.8, 24.1; ESI-MS for $C_{19}H_{16}Cl_2N_4O_2$: calcd 402.1, found m/z 403.2, 405.2 $[M + H]^+$. UPLC-MS generic method: t_R = 4.47 min, @215nm 98%, @254nm 99.5% purity.

NMR spectra and UPLC-MS chromatograms

Figure S14. ^{1}H-NMR spectrum (400 MHz, DMSO-d_6) of **1**

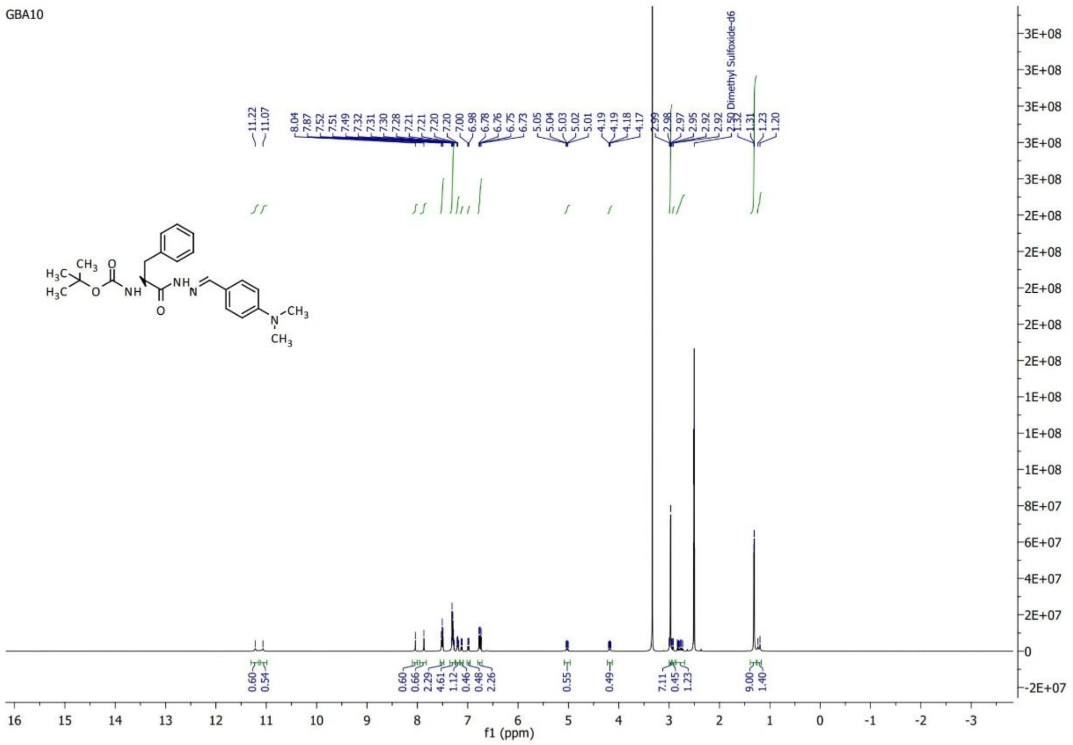

Figure S15. ^{13}C-NMR spectrum (101 MHz, DMSO-d_6) of **1**

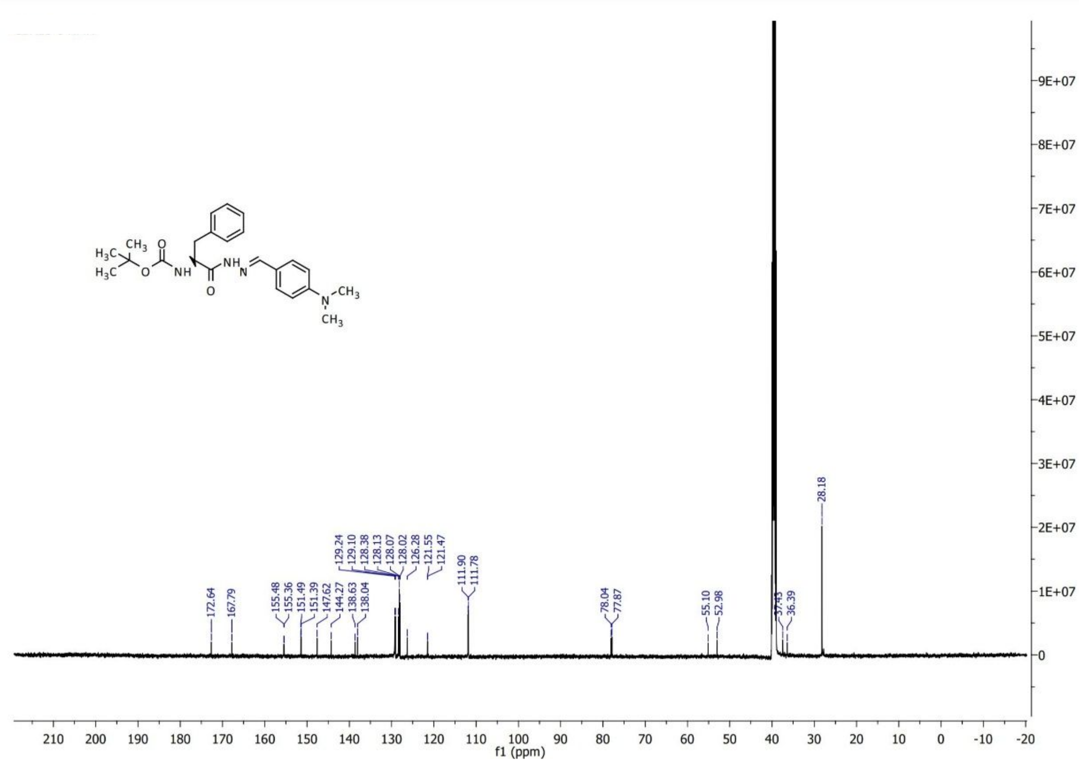

Figure S16. ^{1}H-^{1}H COSY-NMR spectrum (400 MHz, DMSO-d_6) of **1**

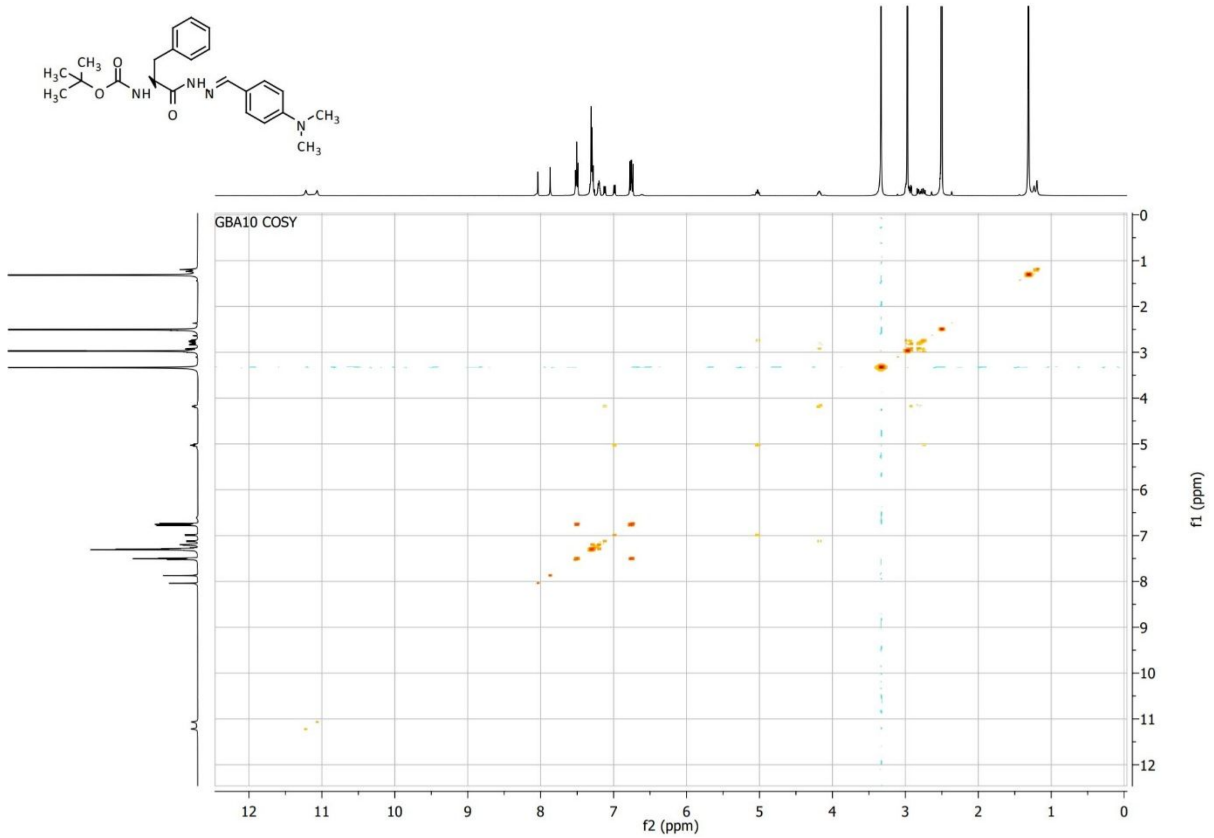

Figure S17. ^{1}H-^{13}C HSQC-NMR spectrum (400 MHz, DMSO-d_6) of **1**

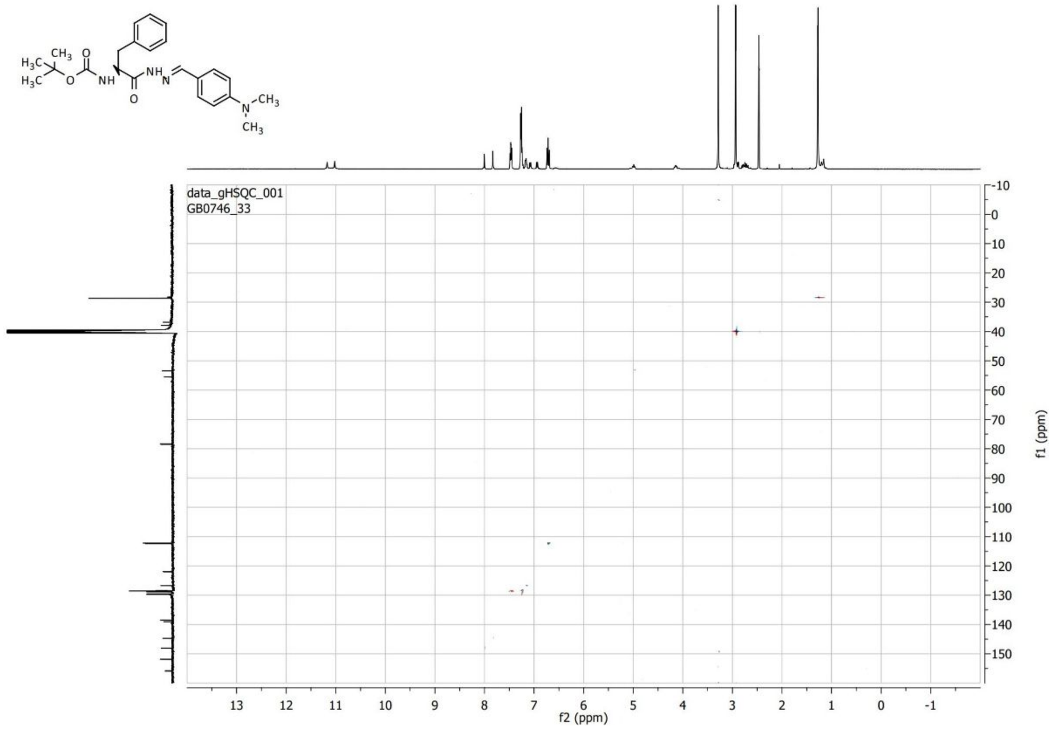

Figure S18. UPLC-MS analysis of **1**

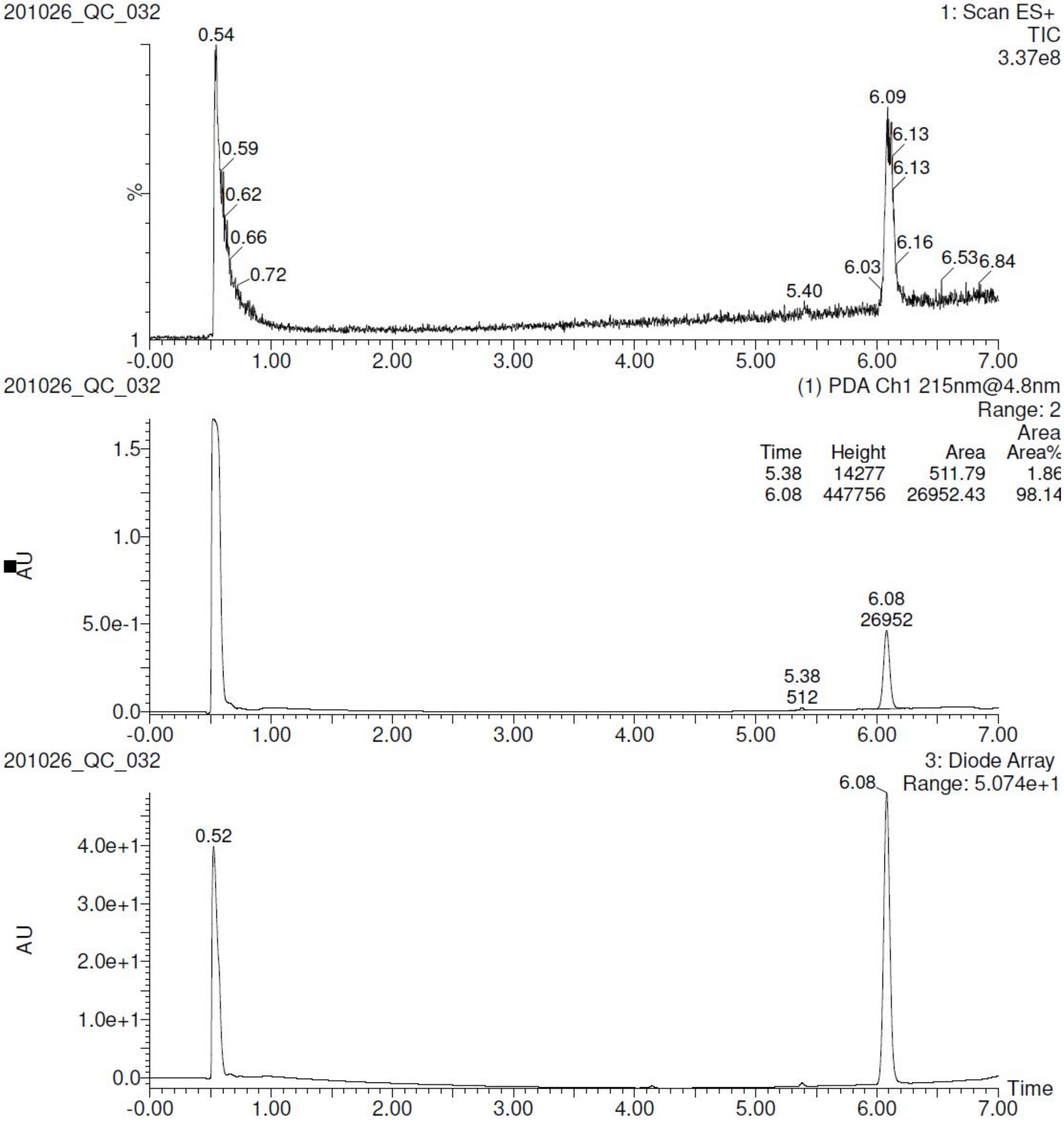

Figure S19. ^{1}H-NMR spectrum (400 MHz, DMSO-d_6) of **2**

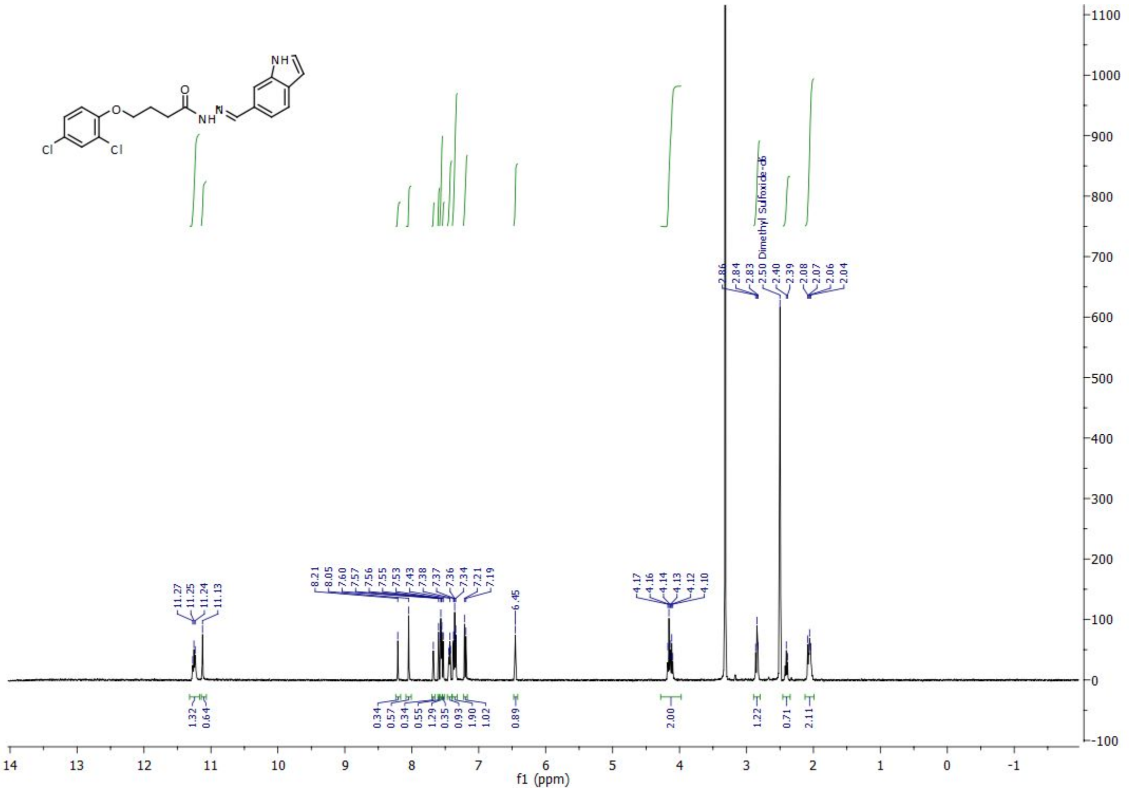

Figure S20. ^{13}C-NMR spectrum (101 MHz, DMSO-d_6) of **2**

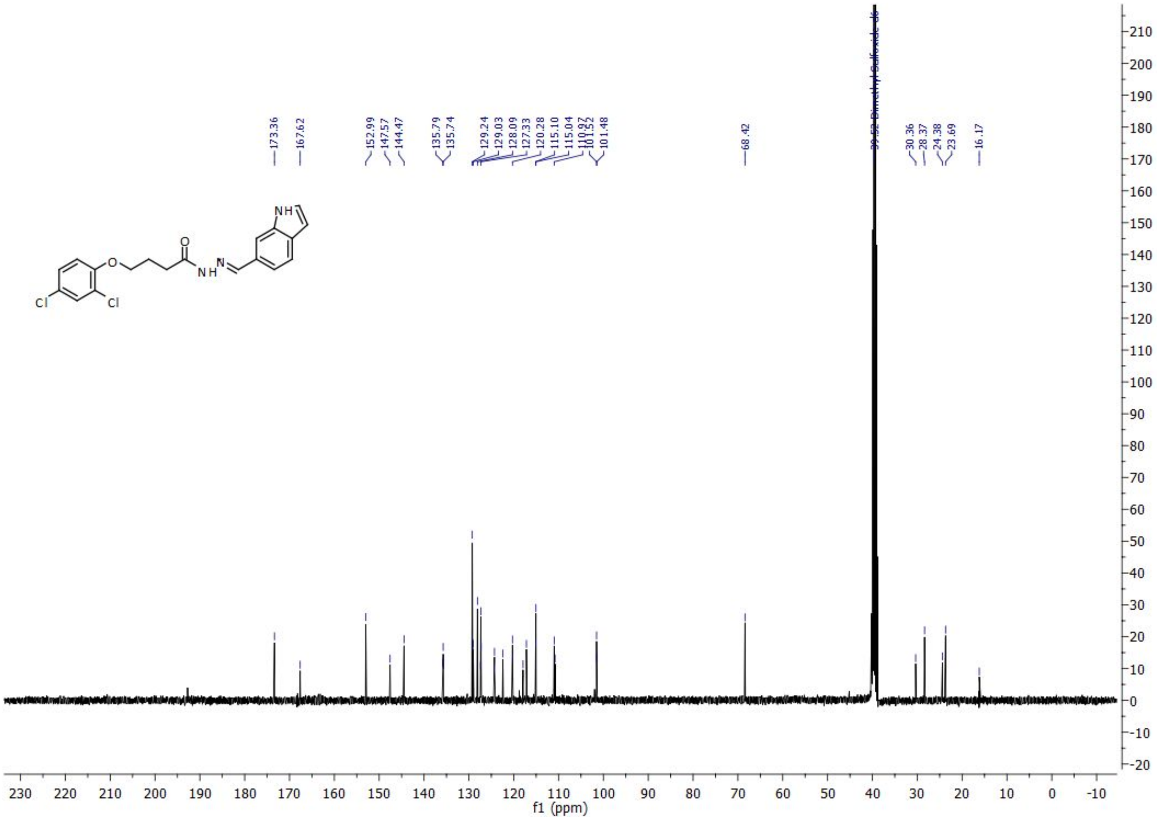

Figure S21. ^{1}H-^{1}H COSY-NMR spectrum (400 MHz, DMSO-d_6) of **2**

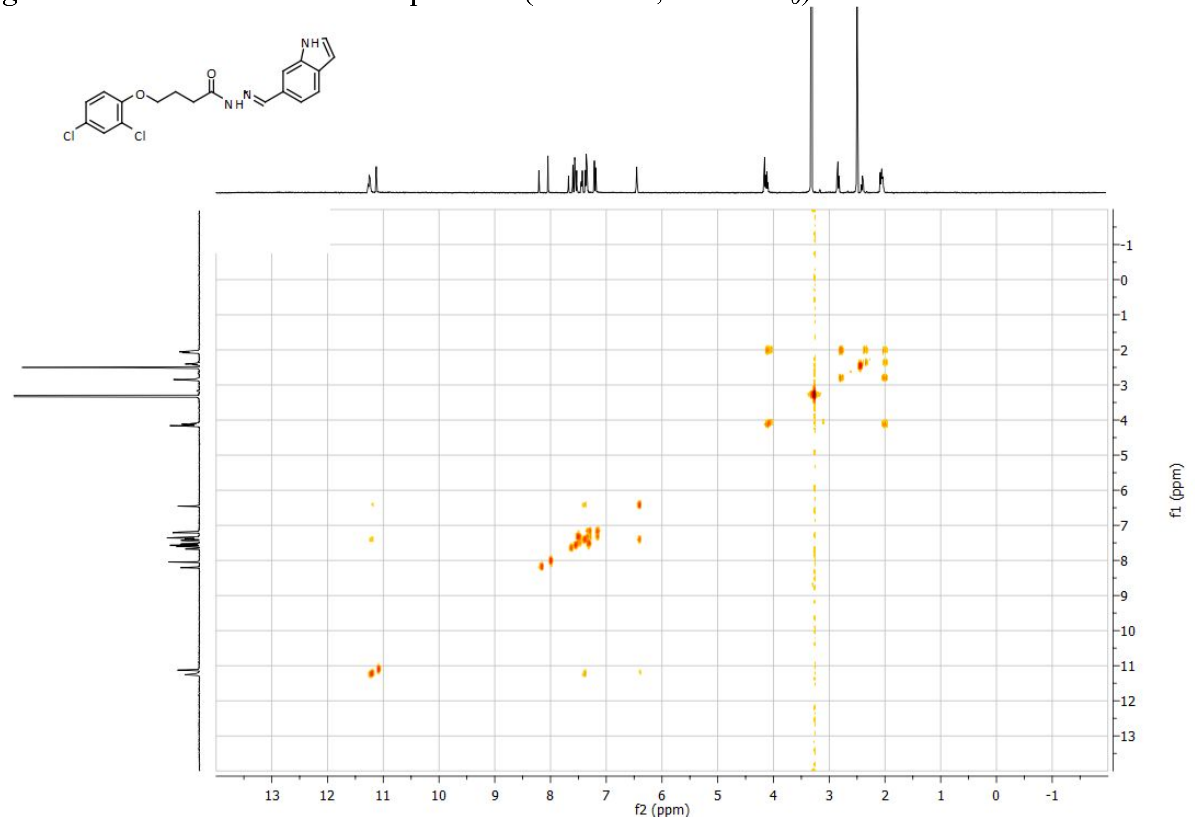

Figure S22. ^{1}H-^{13}C HSQC-NMR spectrum (400 MHz, DMSO-d_6) of **2**

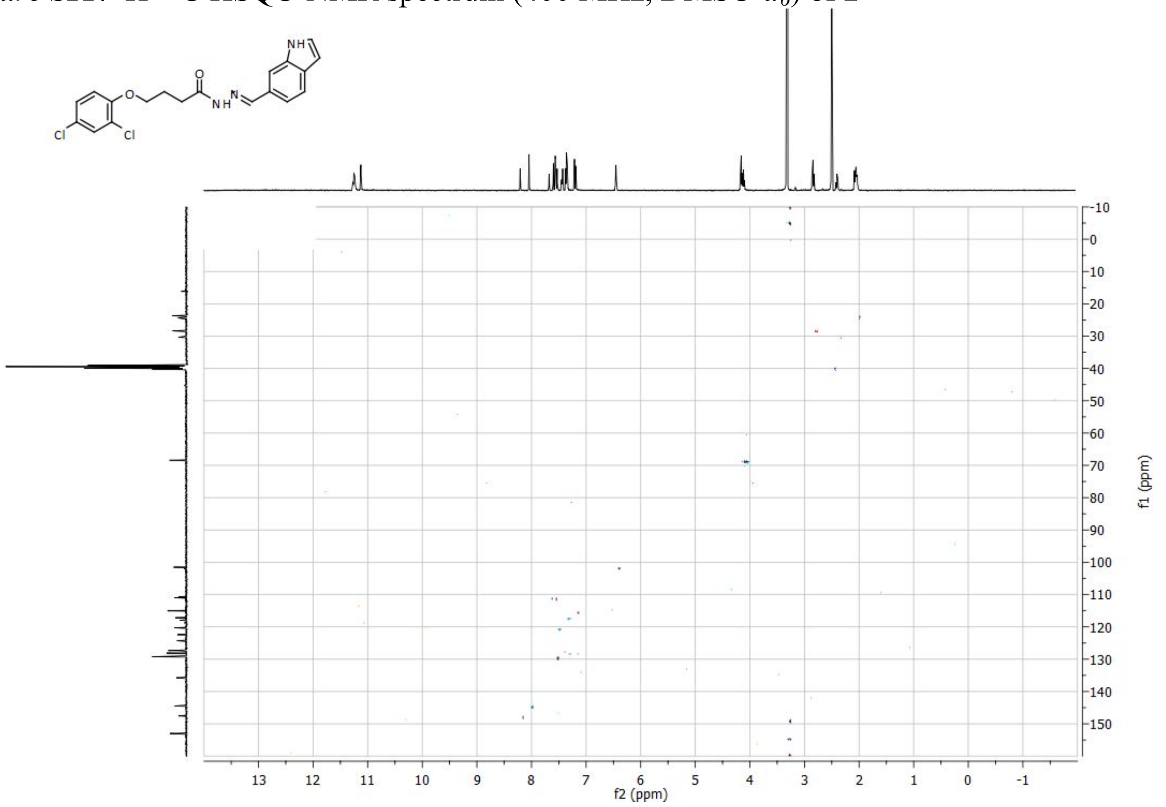

Figure S23. UPLC-MS analysis of **2**

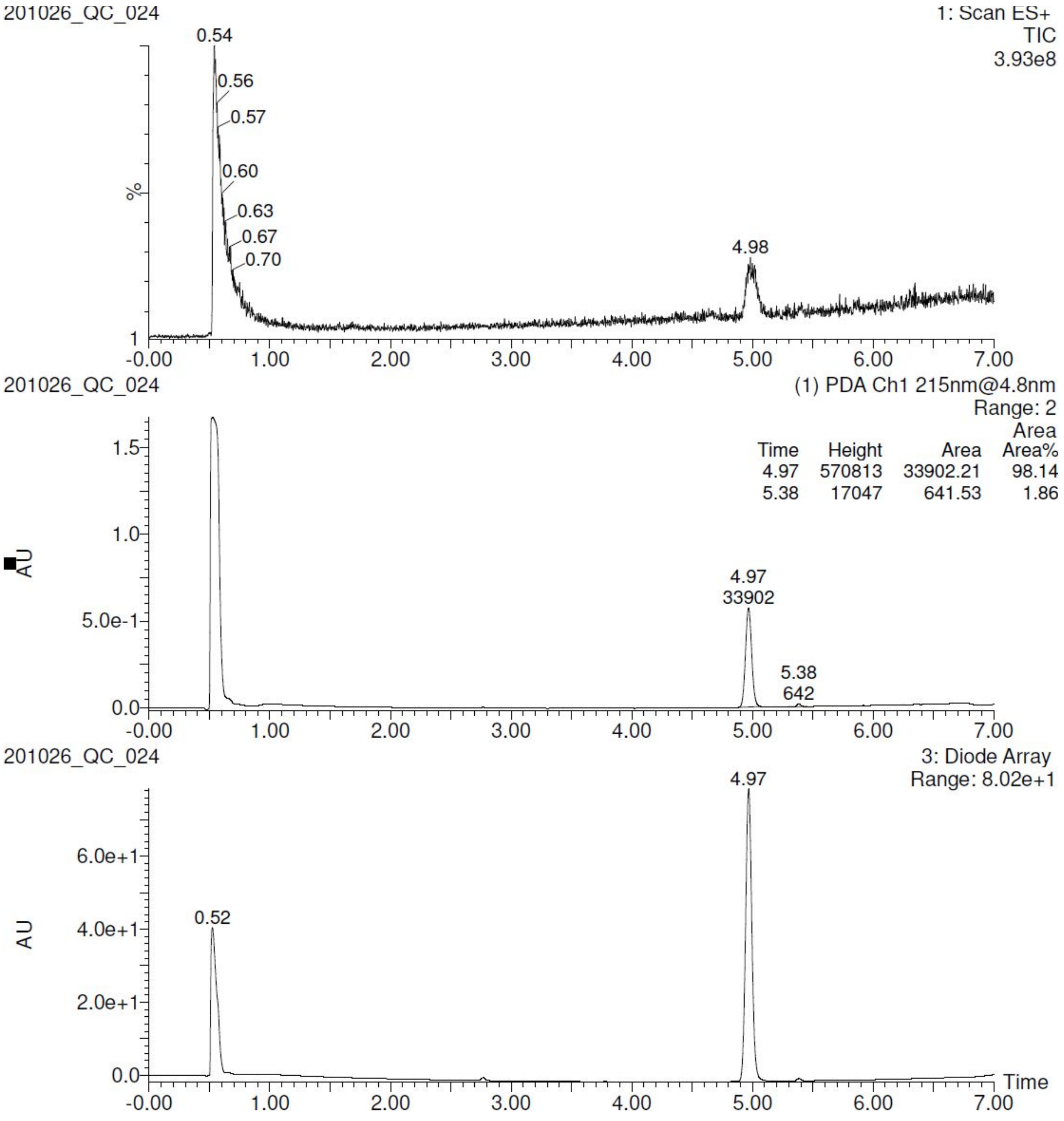

Figure S24. ^{1}H-NMR spectrum (400 MHz, DMSO-d_6) of **3**

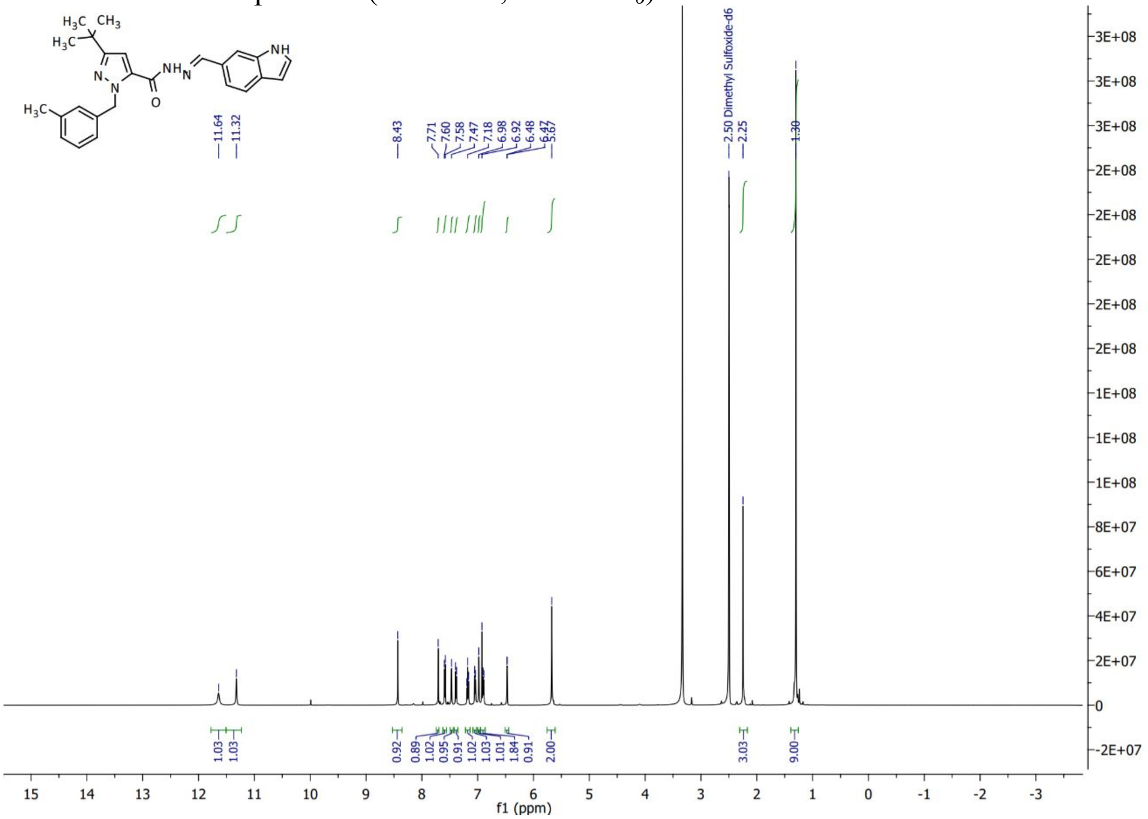

Figure S25. ^{13}C-NMR spectrum (101 MHz, DMSO-d_6) of **3**

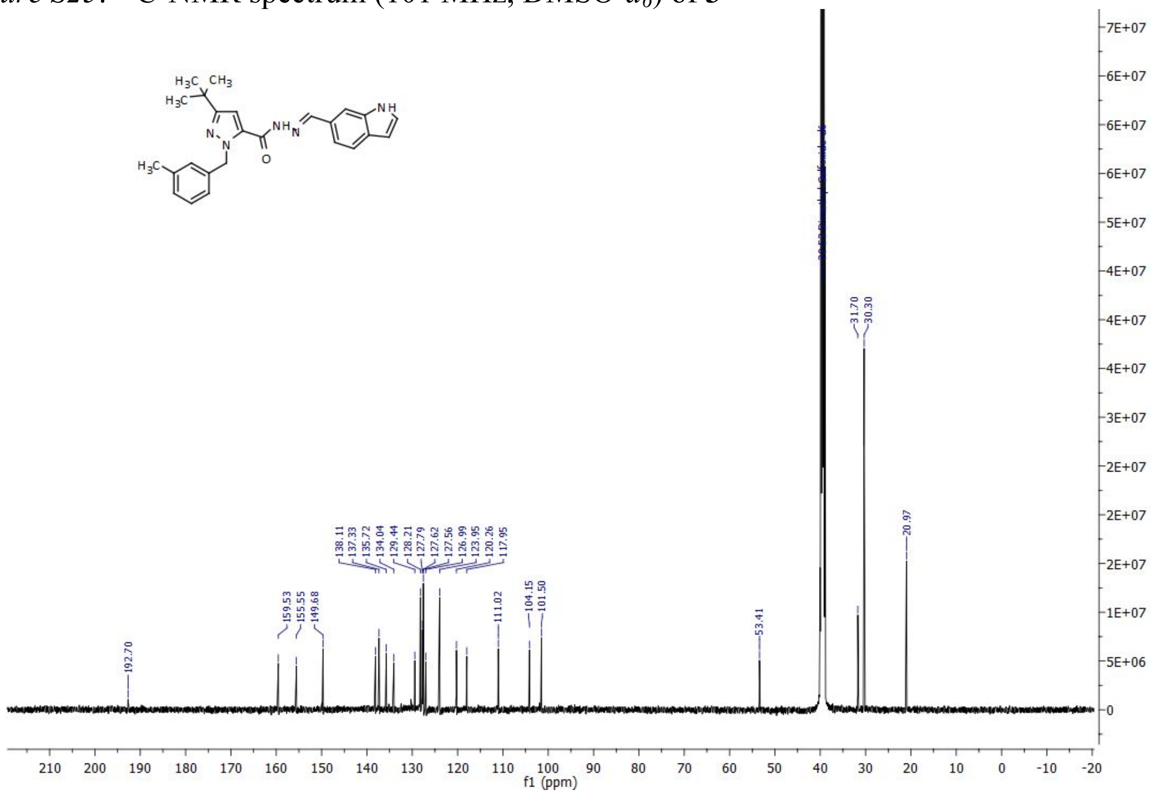

Figure S26. ^{1}H-^{1}H COSY-NMR spectrum (400 MHz, DMSO-d_6) of **3**

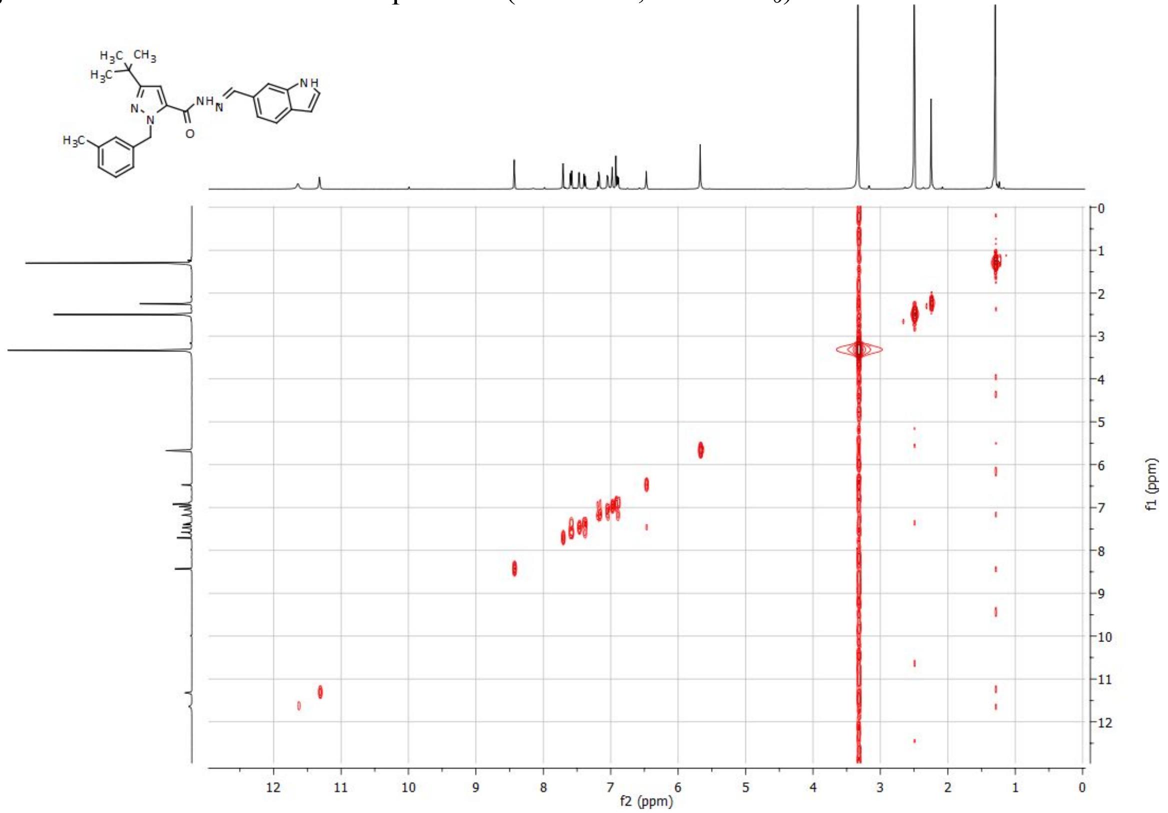

Figure S27. ^{1}H-^{13}C HSQC-NMR spectrum (400 MHz, DMSO-d_6) of **3**

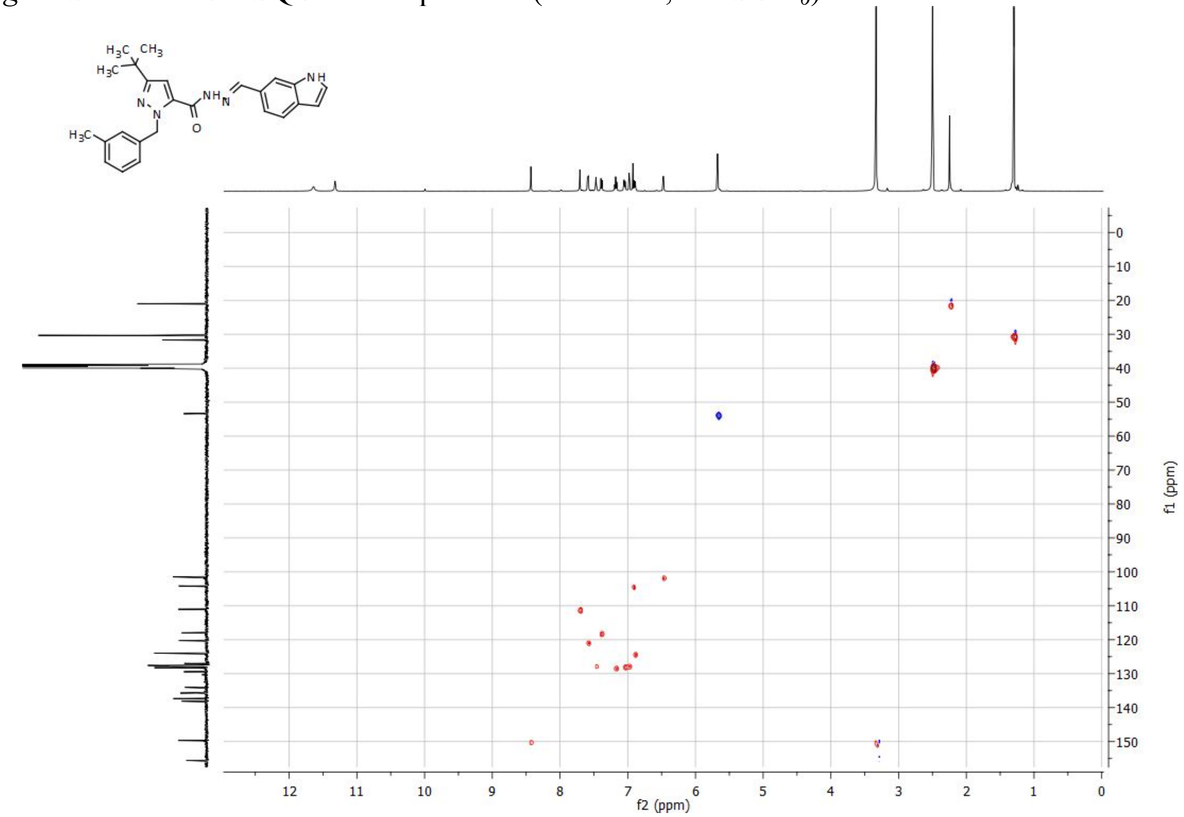

Figure S28. UPLC-MS analysis of **3**

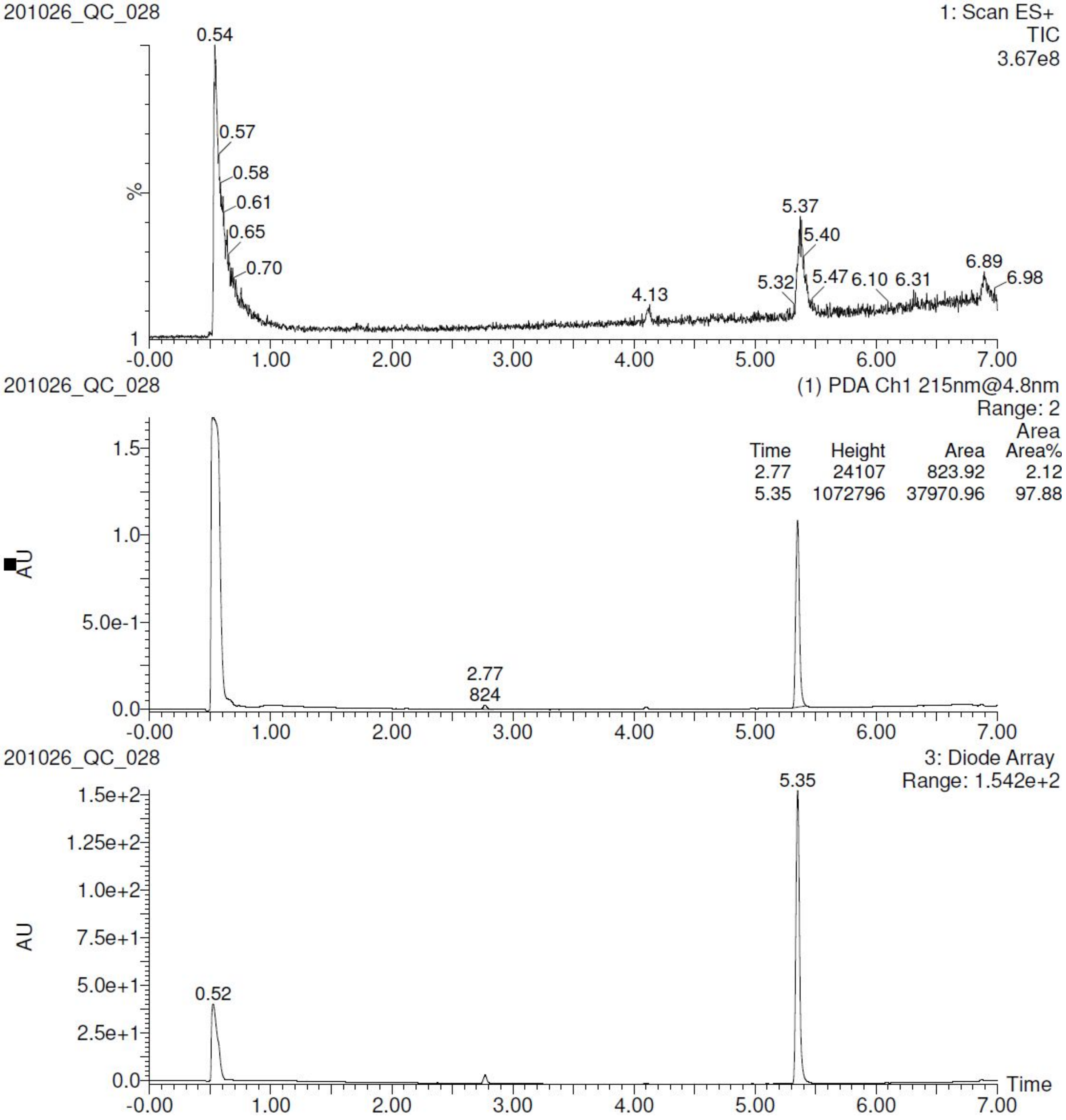

Figure S29. ^{1}H-NMR spectrum (400 MHz, DMSO-d_6) of **4**

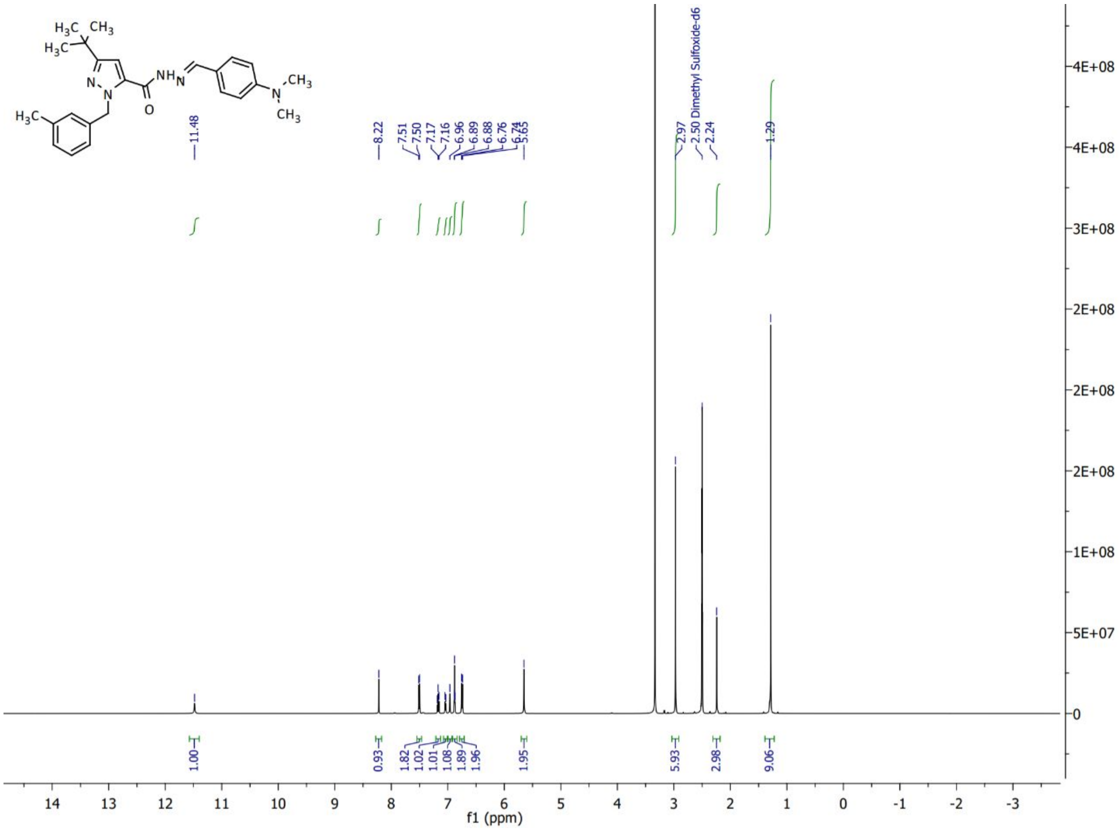

Figure S30. ^{13}C-NMR spectrum (101 MHz, DMSO-d_6) of **4**

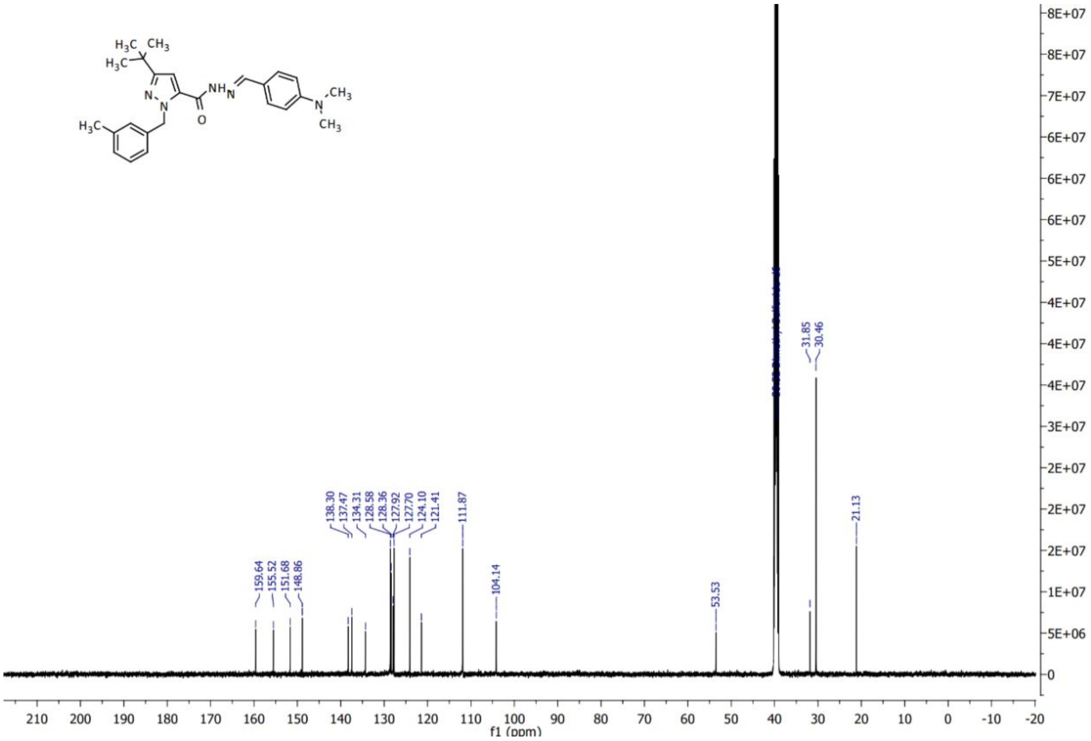

Figure S31. ^{1}H-^{1}H COSY-NMR spectrum (400 MHz, DMSO-d_6) of **4**

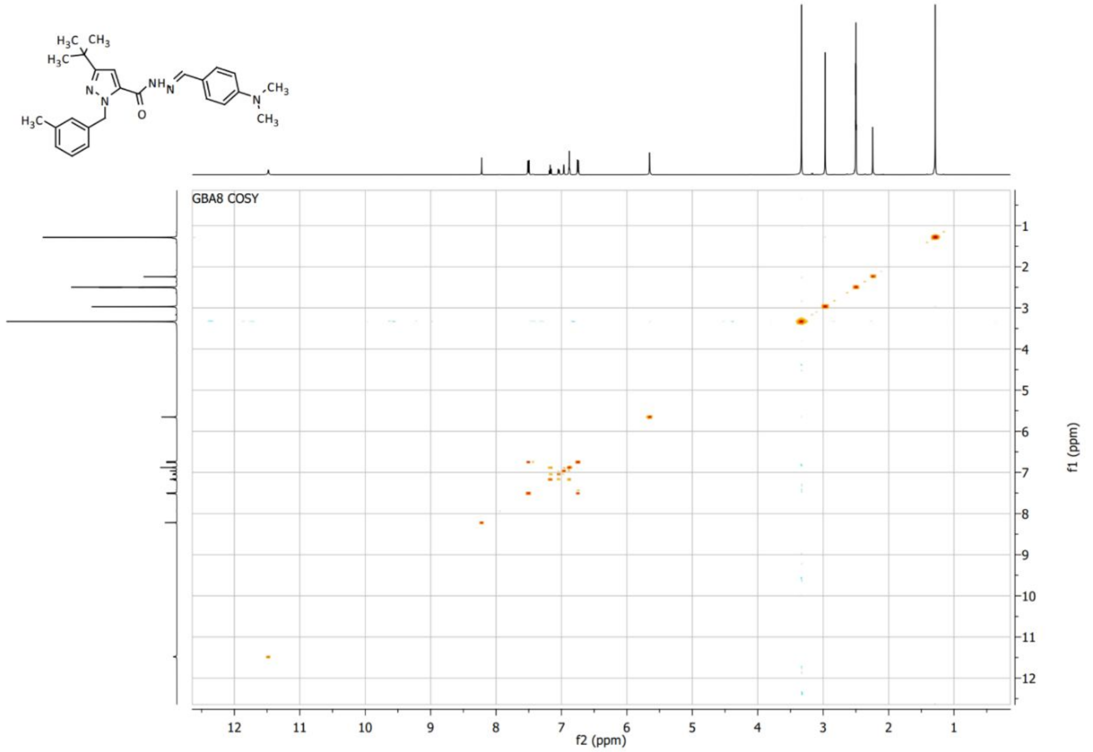

Figure S32. ^{1}H-^{13}C HSQC-NMR spectrum (400 MHz, DMSO-d_6) of **4**

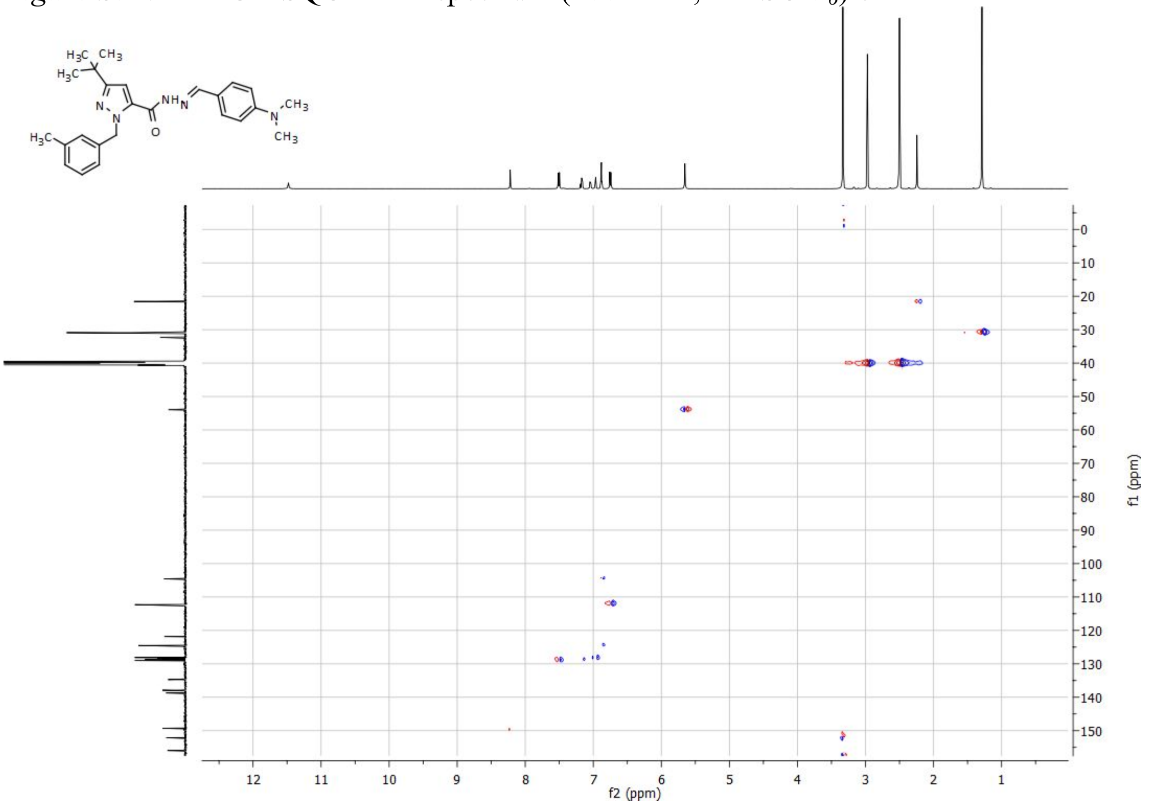

Figure S33. UPLC-MS analysis of **4**

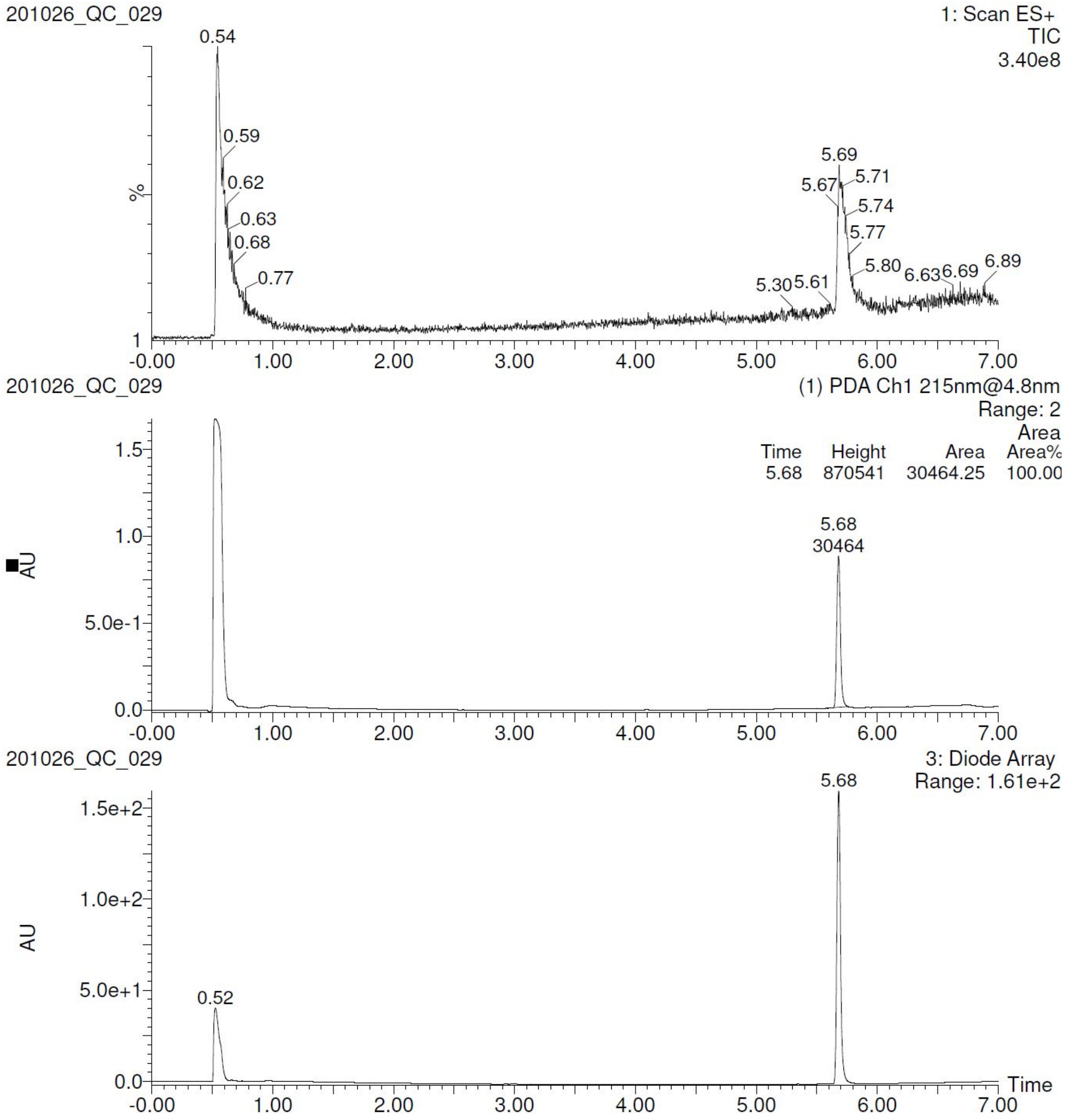

Figure S34. ^{1}H-NMR spectrum (400 MHz, DMSO-d_6) of **5**

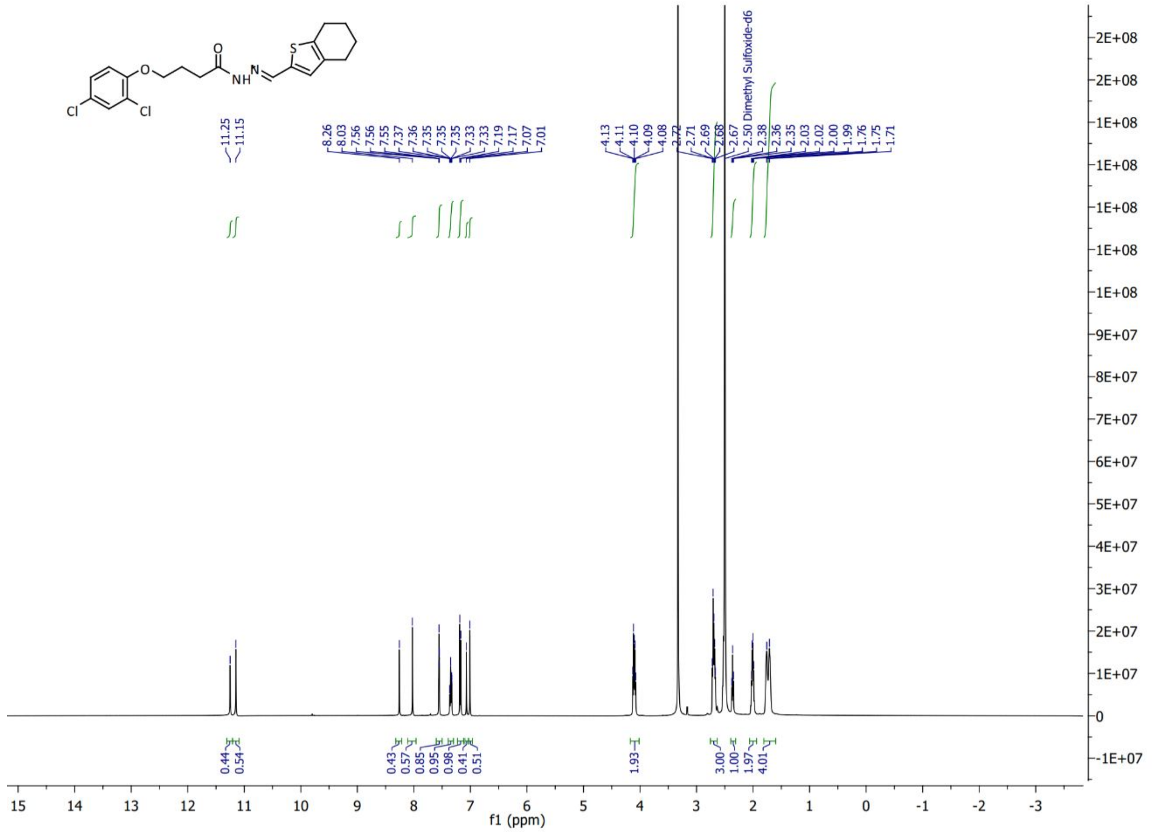

Figure S35. ^{13}C-NMR spectrum (101 MHz, DMSO-d_6) of **5**

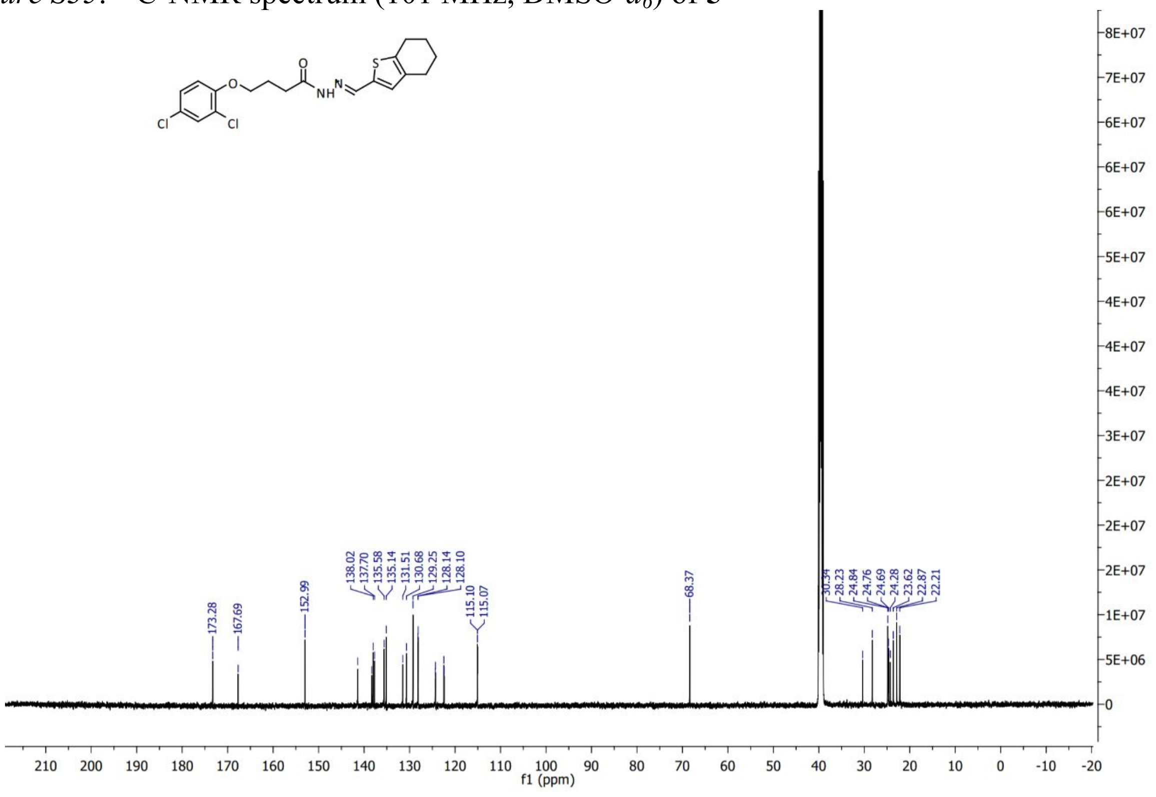

Figure S36. ^{1}H-^{1}H COSY-NMR spectrum (400 MHz, DMSO-d_6) of **5**

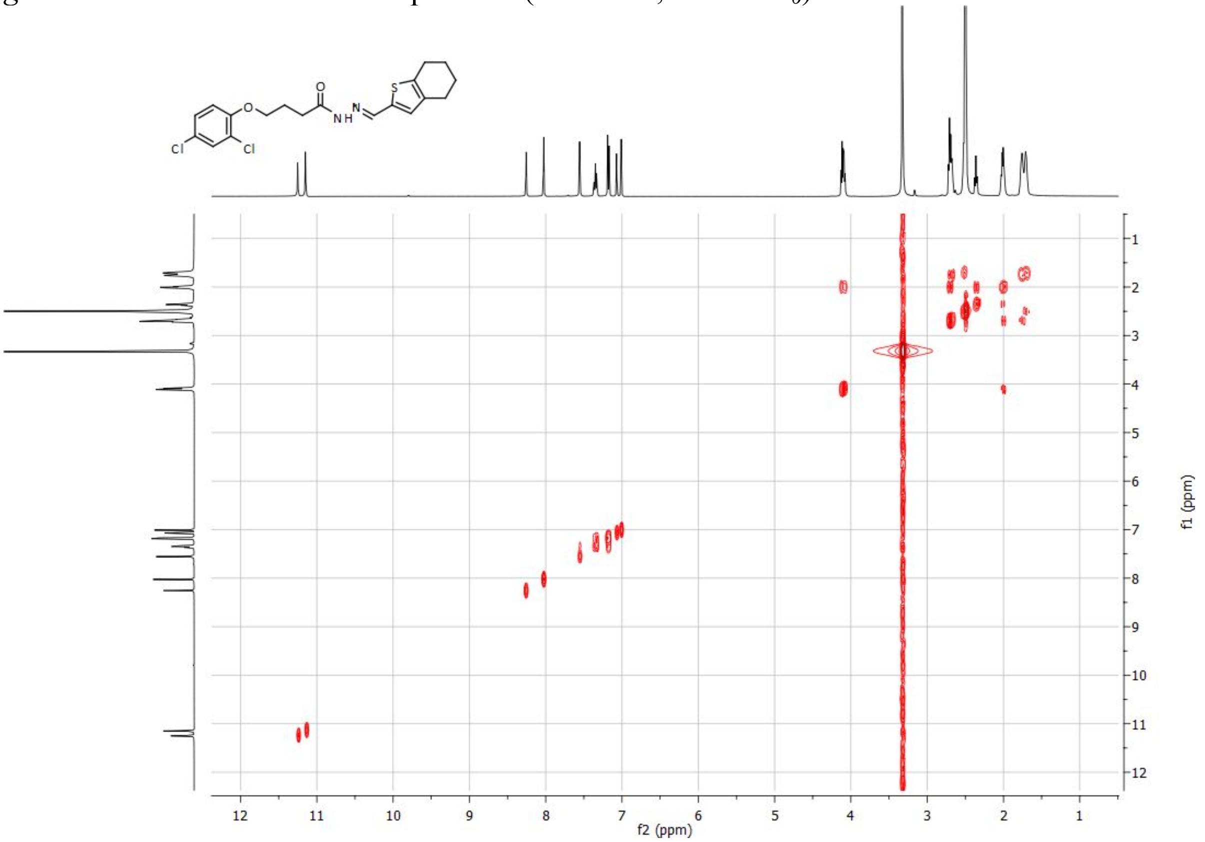

Figure S37. ^{1}H-^{13}C HSQC-NMR spectrum (400 MHz, DMSO-d_6) of **5**

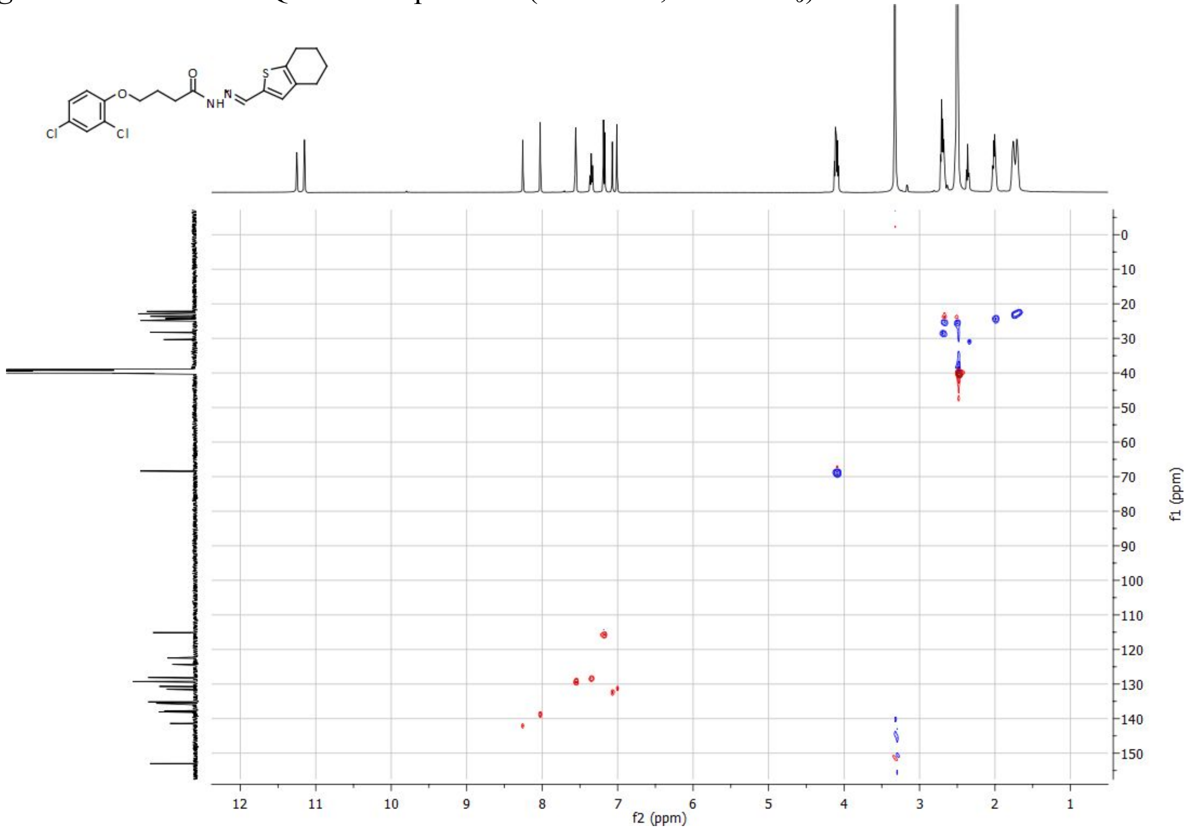

Figure S38. UPLC-MS analysis of **5**

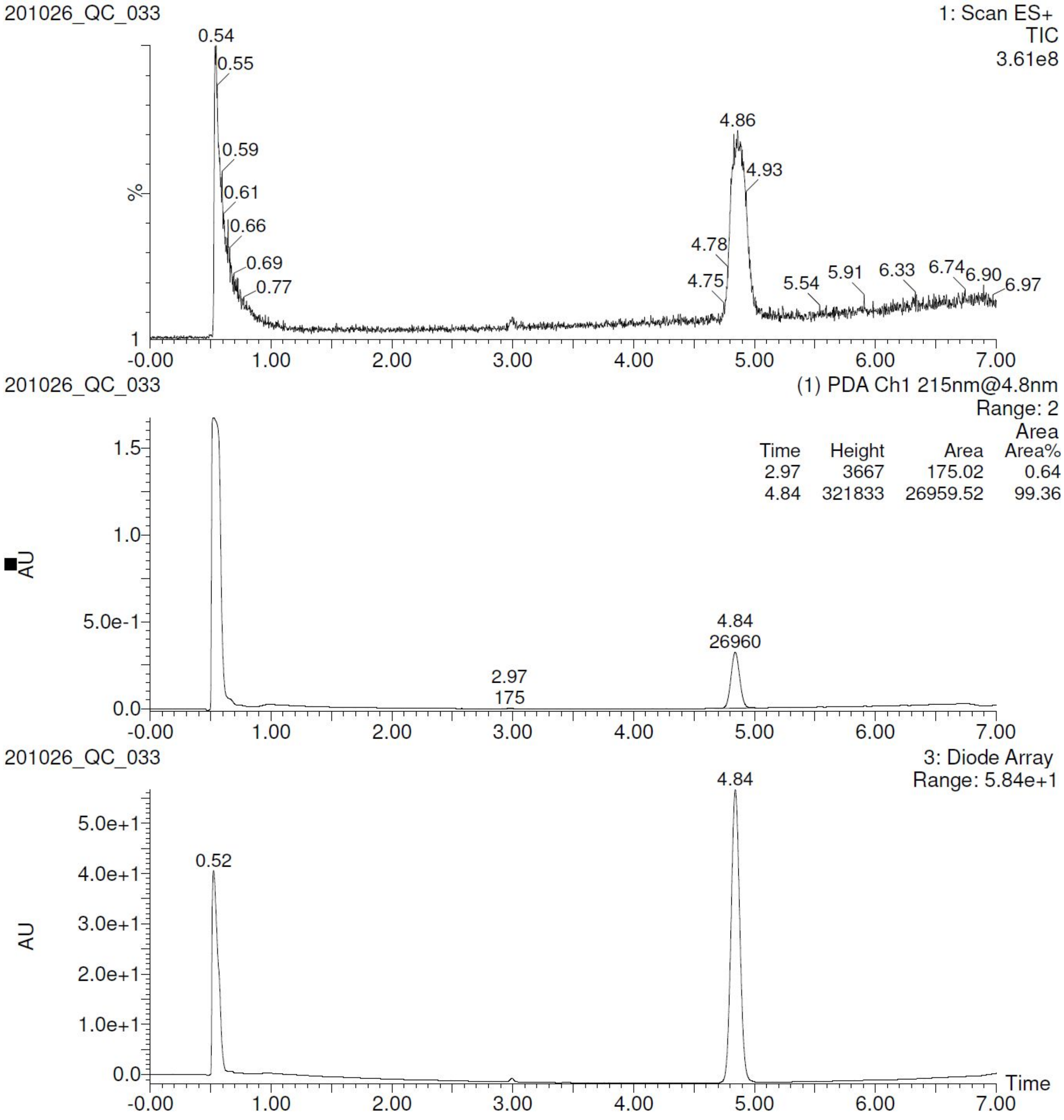

Figure S39. ^{1}H-NMR spectrum (400 MHz, DMSO-d_6) of **6**

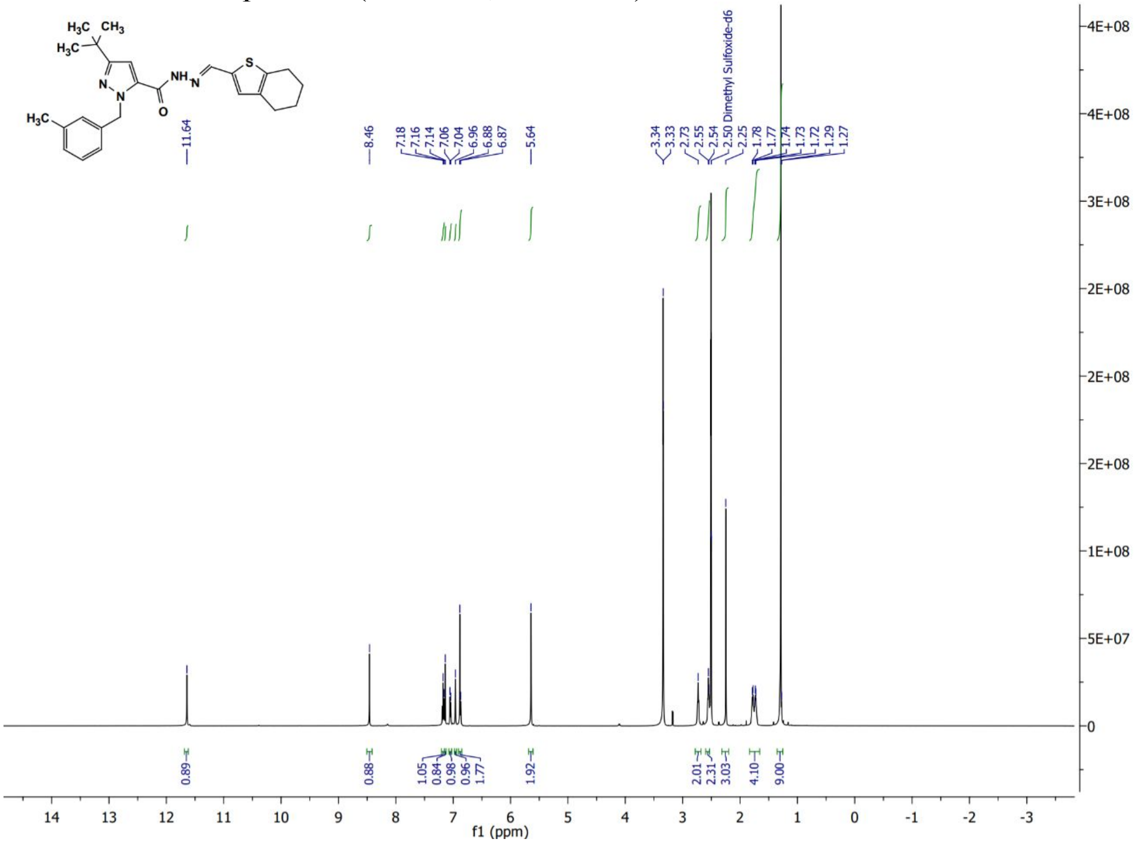

Figure S40. ^{13}C-NMR spectrum (101 MHz, DMSO-d_6) of **6**

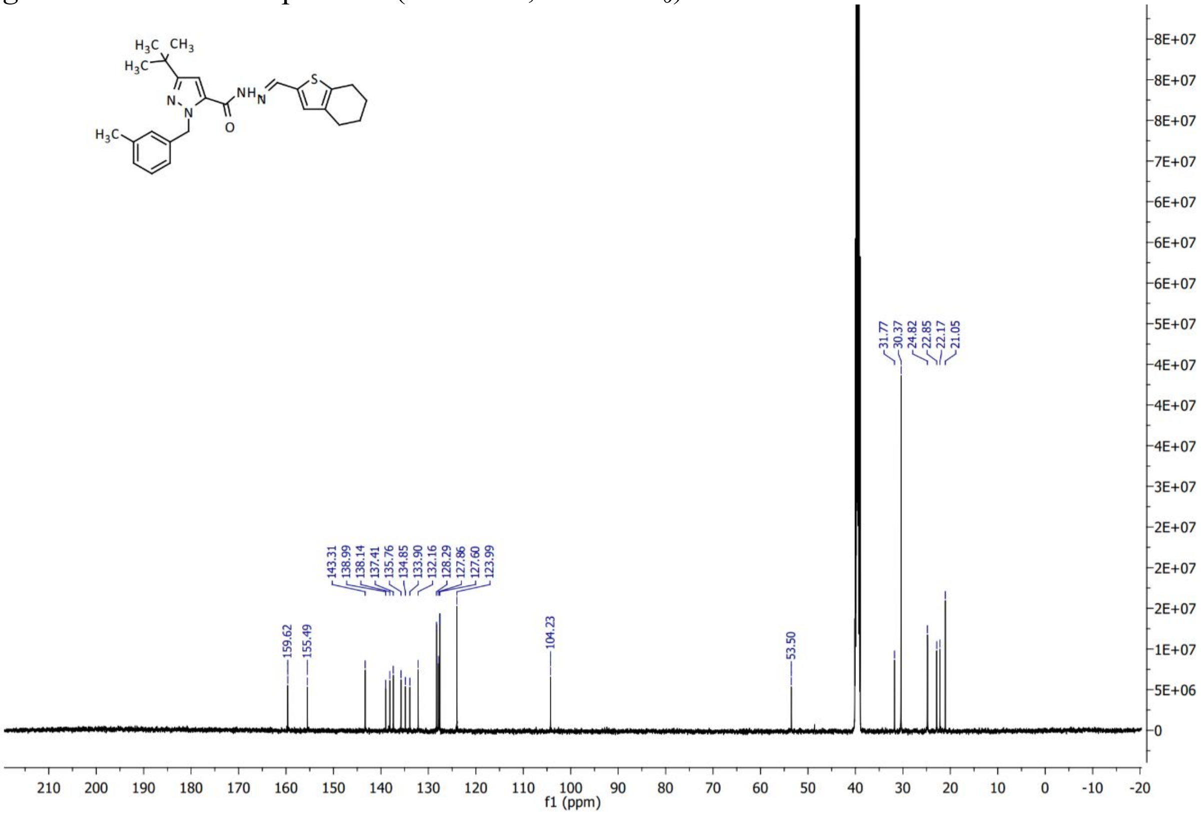

Figure S41. ^{1}H-^{1}H COSY-NMR spectrum (400 MHz, DMSO-d_6) of **6**

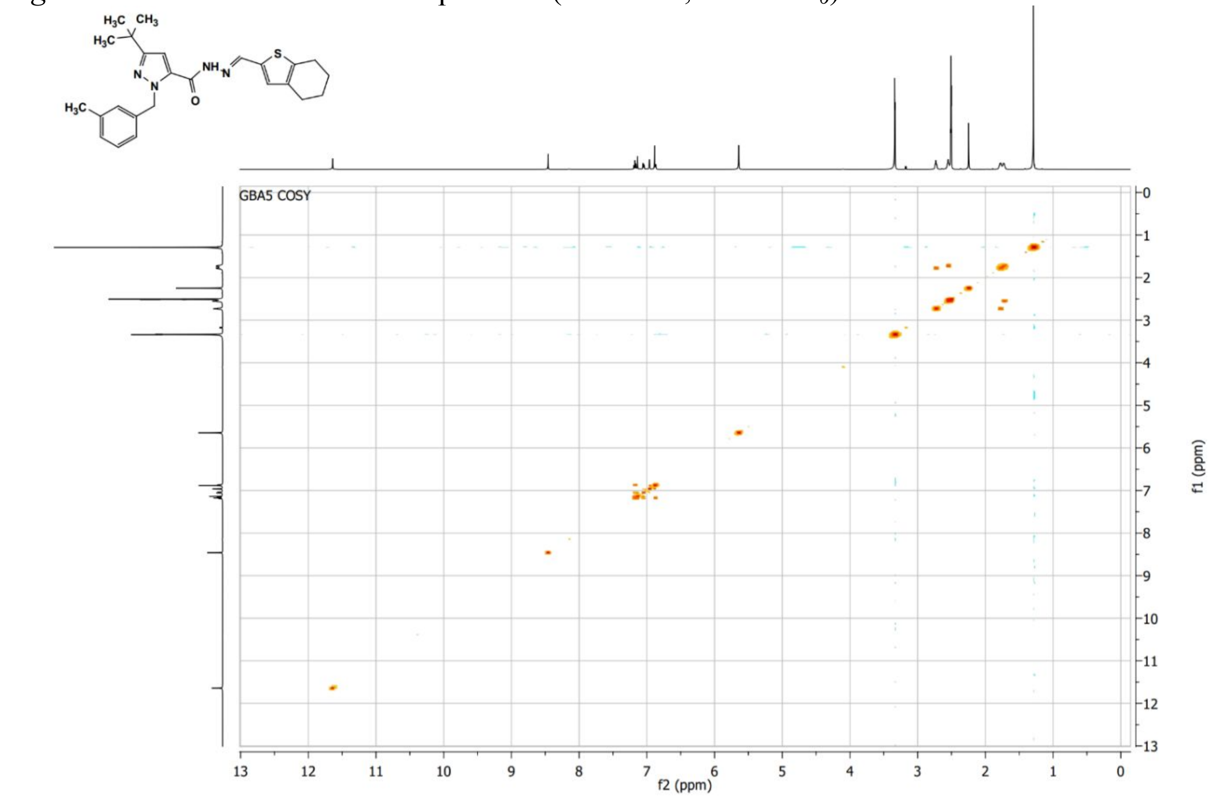

Figure S42. ^{1}H-^{13}C HSQC-NMR spectrum (400 MHz, DMSO-d_6) of **6**

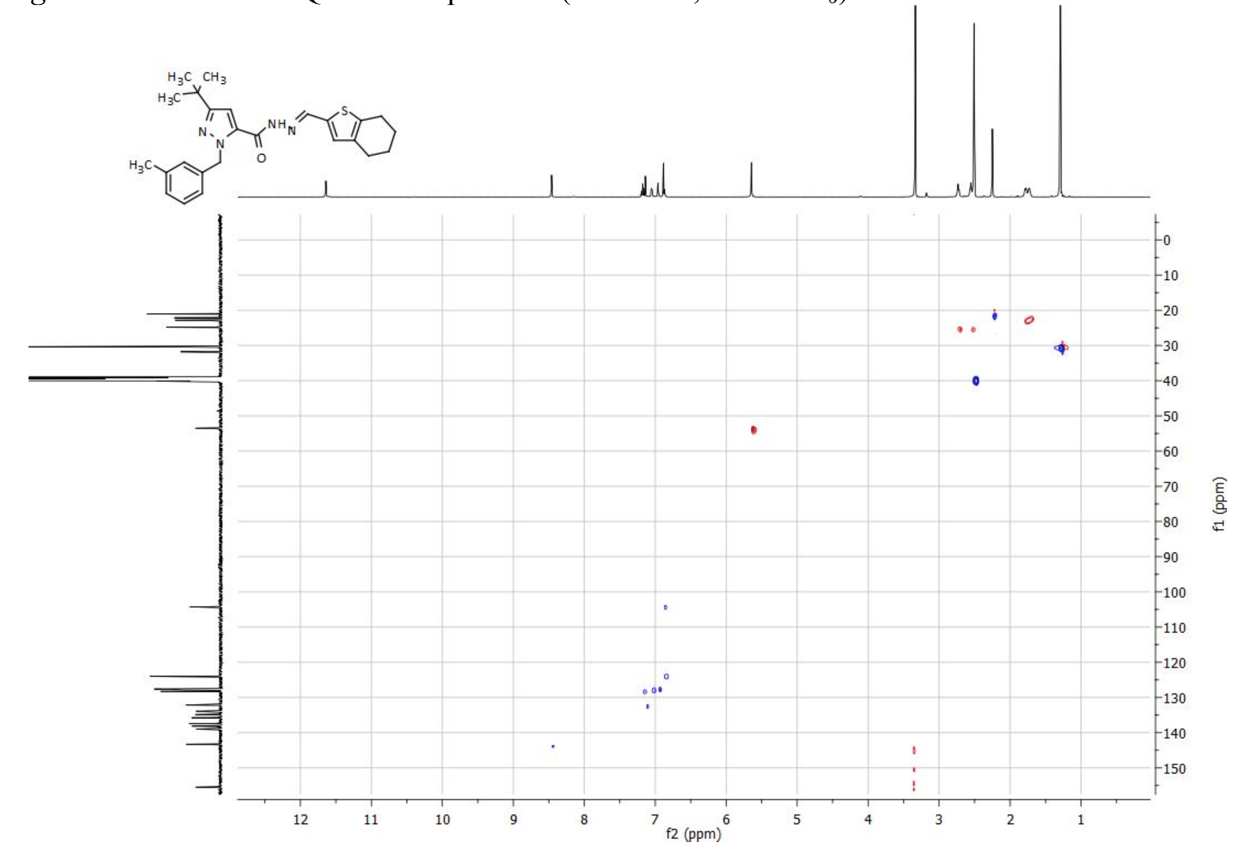

Figure S43. UPLC-MS analysis of **6**

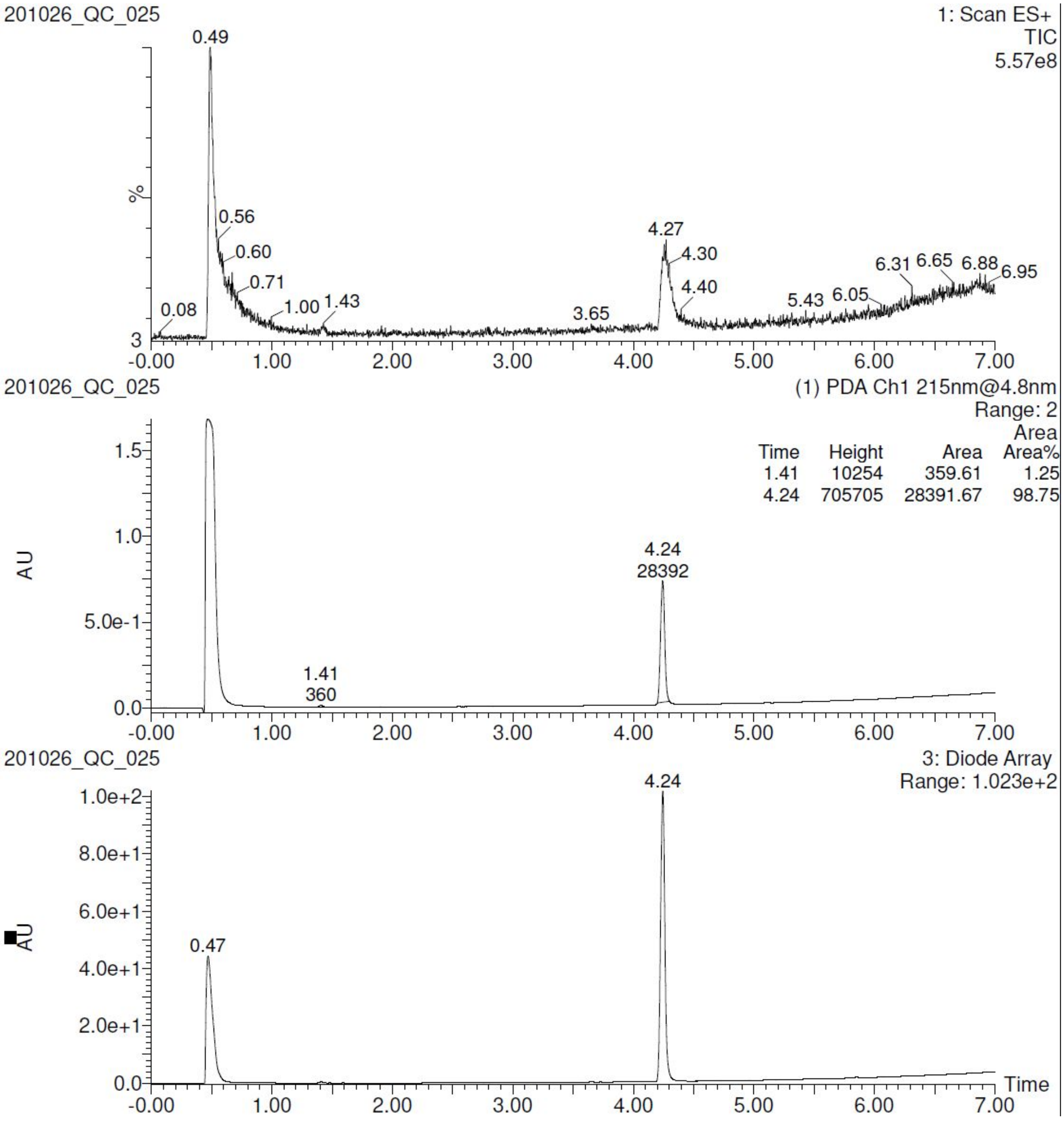

Figure S44. ^{1}H-NMR spectrum (400 MHz, DMSO-d_6) of **7**

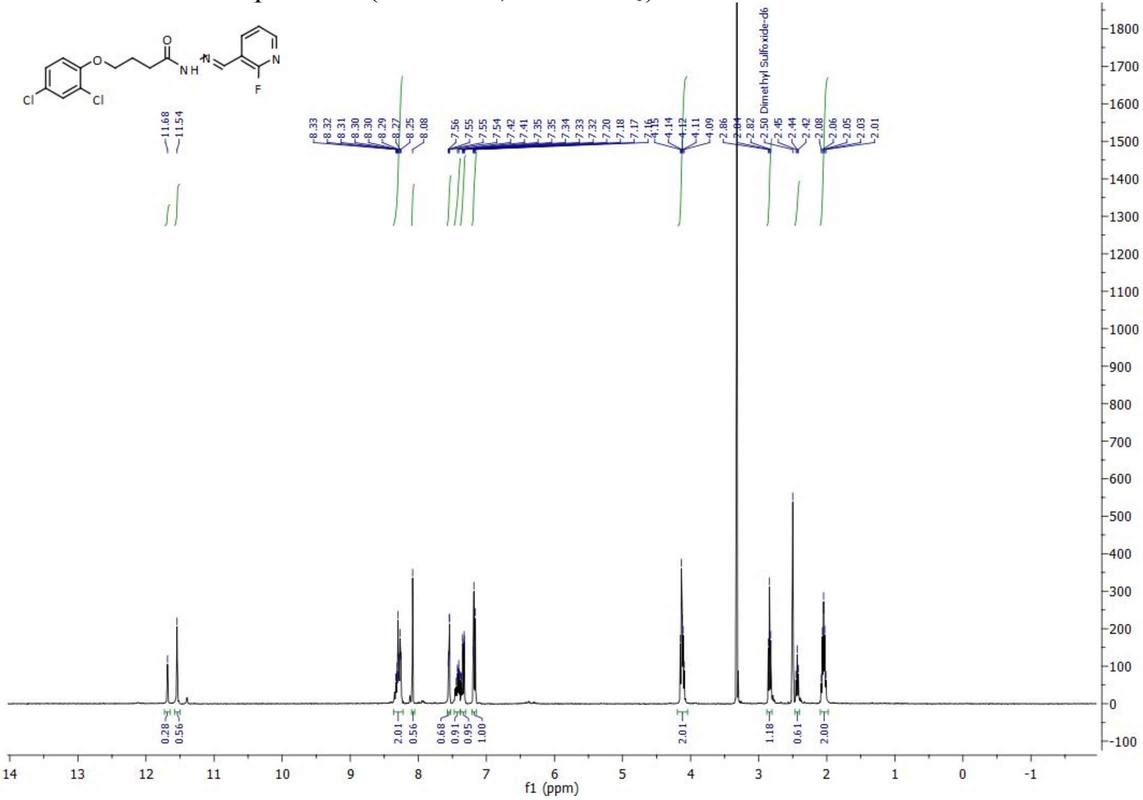

Figure S45. ^{13}C-NMR spectrum (101 MHz, DMSO-d_6) of **7**

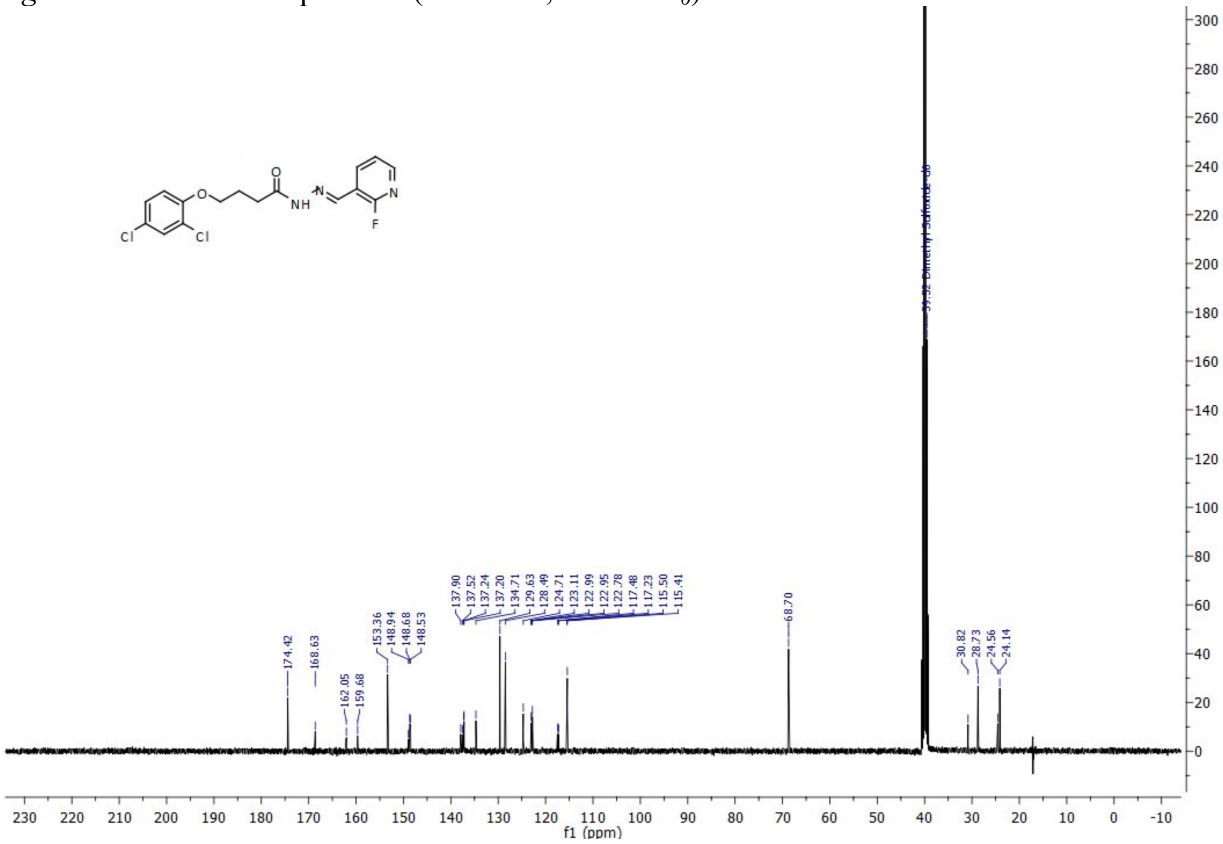

Figure S46. ^{1}H-^{1}H COSY-NMR spectrum (400 MHz, DMSO-d_6) of **7**

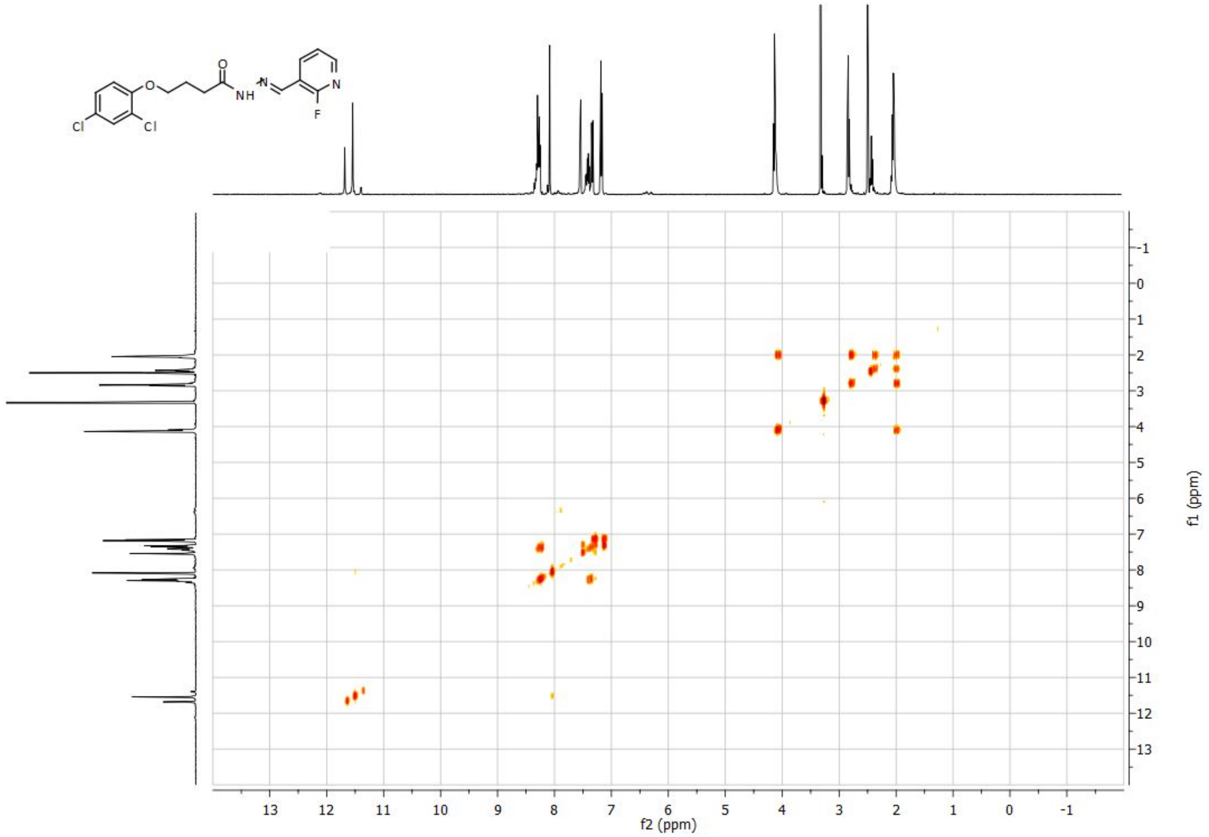

Figure S47. ^{1}H-^{13}C HSQC-NMR spectrum (400 MHz, DMSO-d_6) of **7**

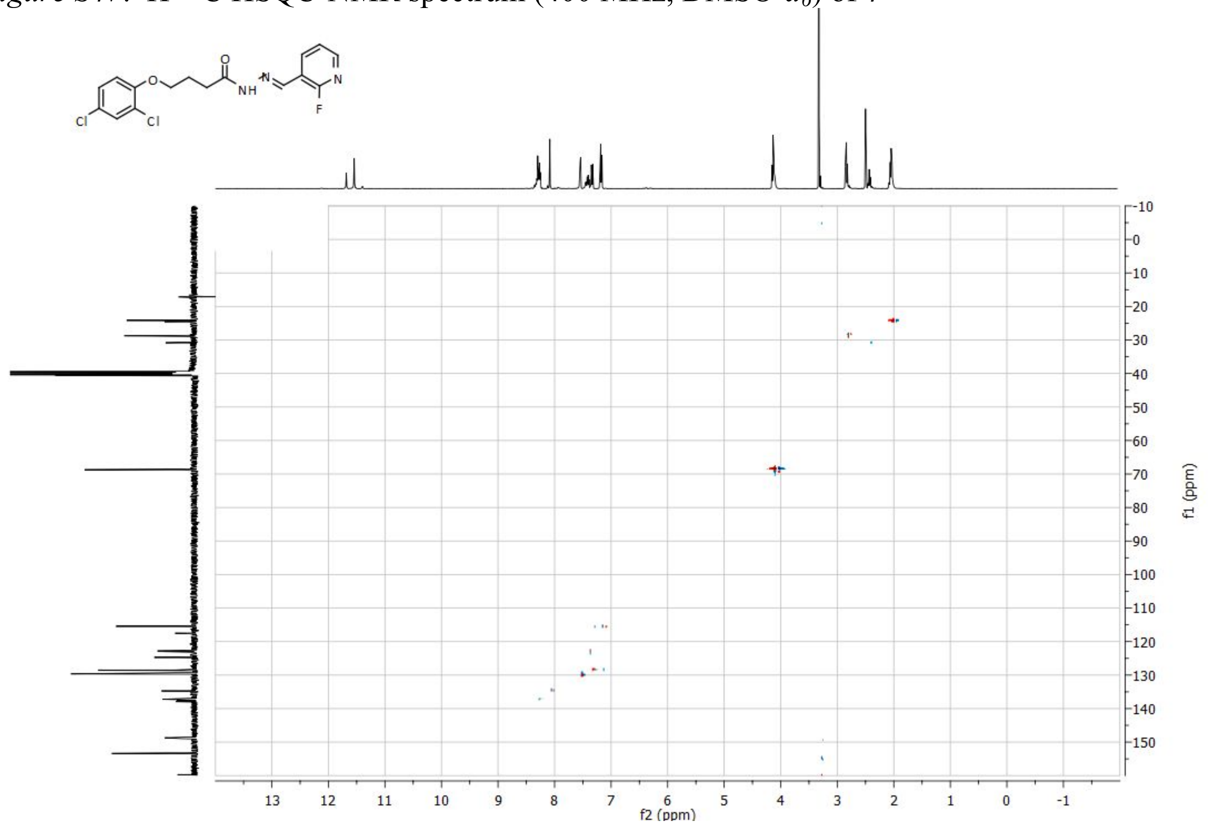

Figure S48. ^{1}H-^{13}C HMBC-NMR spectrum (400 MHz, DMSO-d_6) of **7**

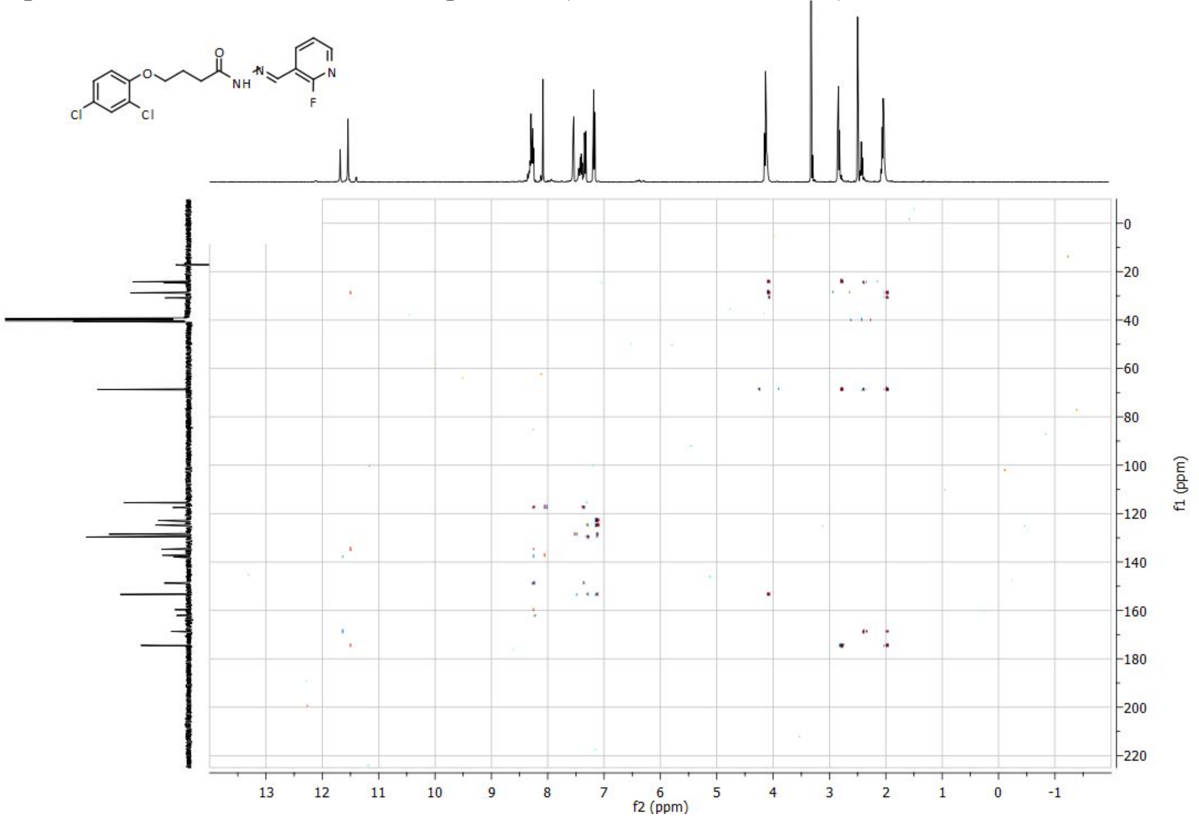

Figure S49. UPLC-MS analysis of **7**

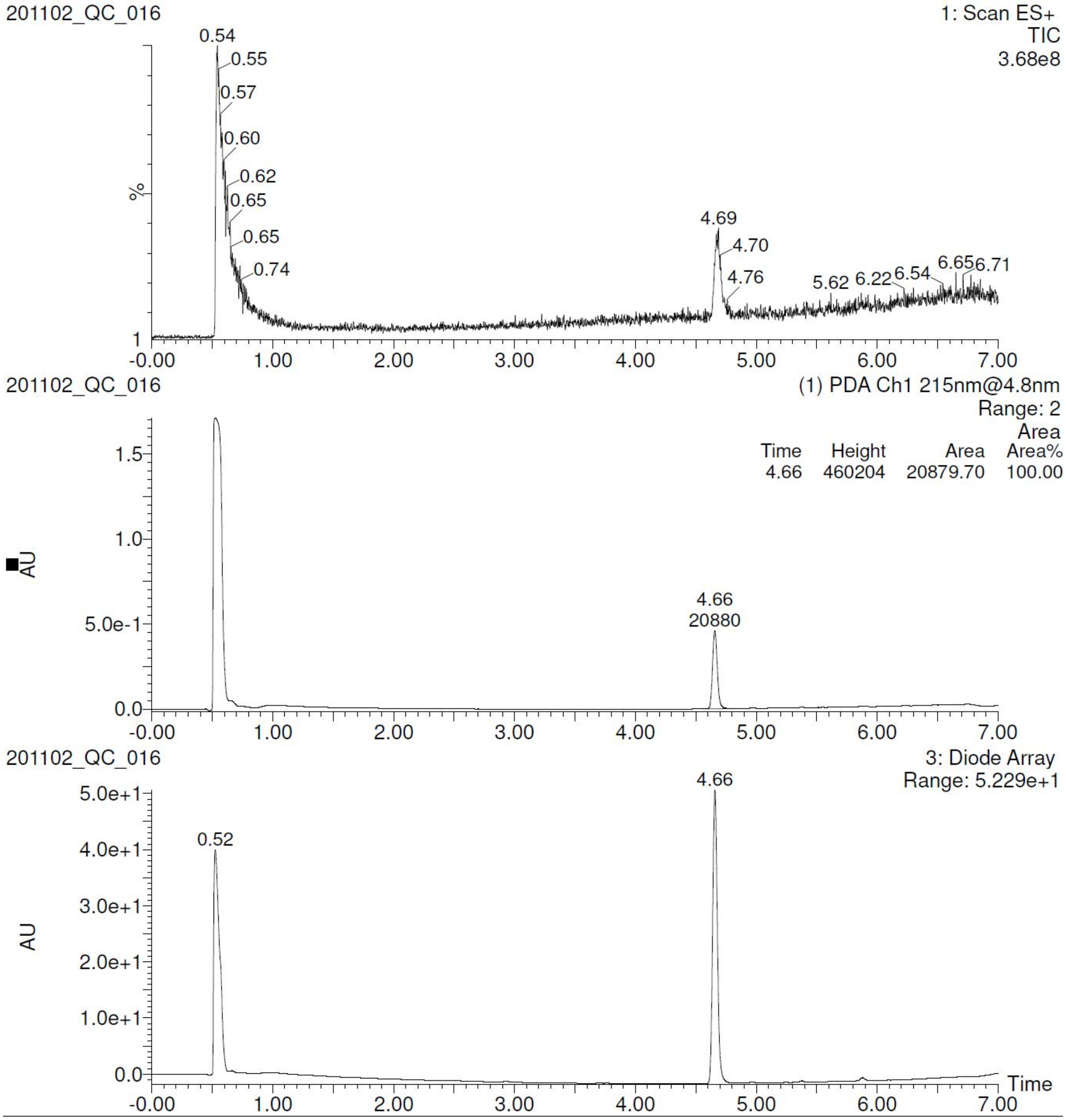

Figure S50. ^{1}H-NMR spectrum (400 MHz, DMSO-d_6) of **8**

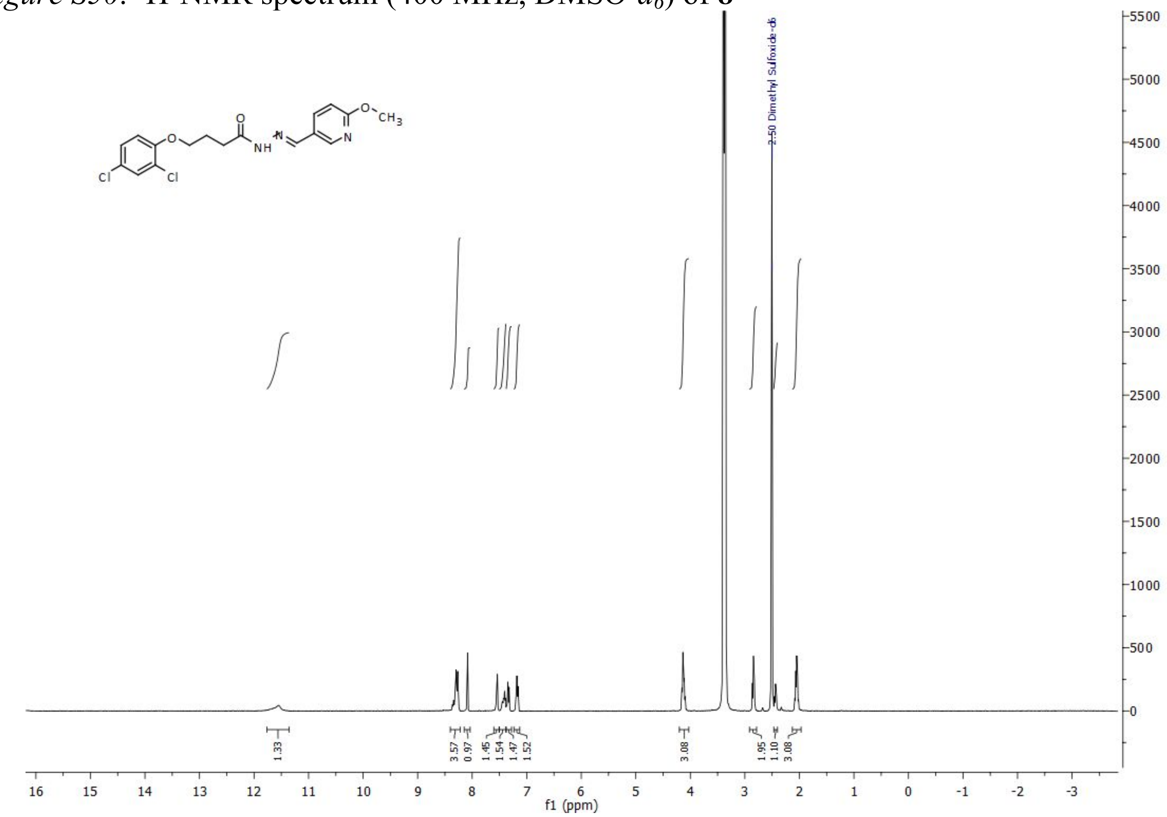

Figure S51. ^{13}C-NMR spectrum (101 MHz, DMSO-d_6) of **8**

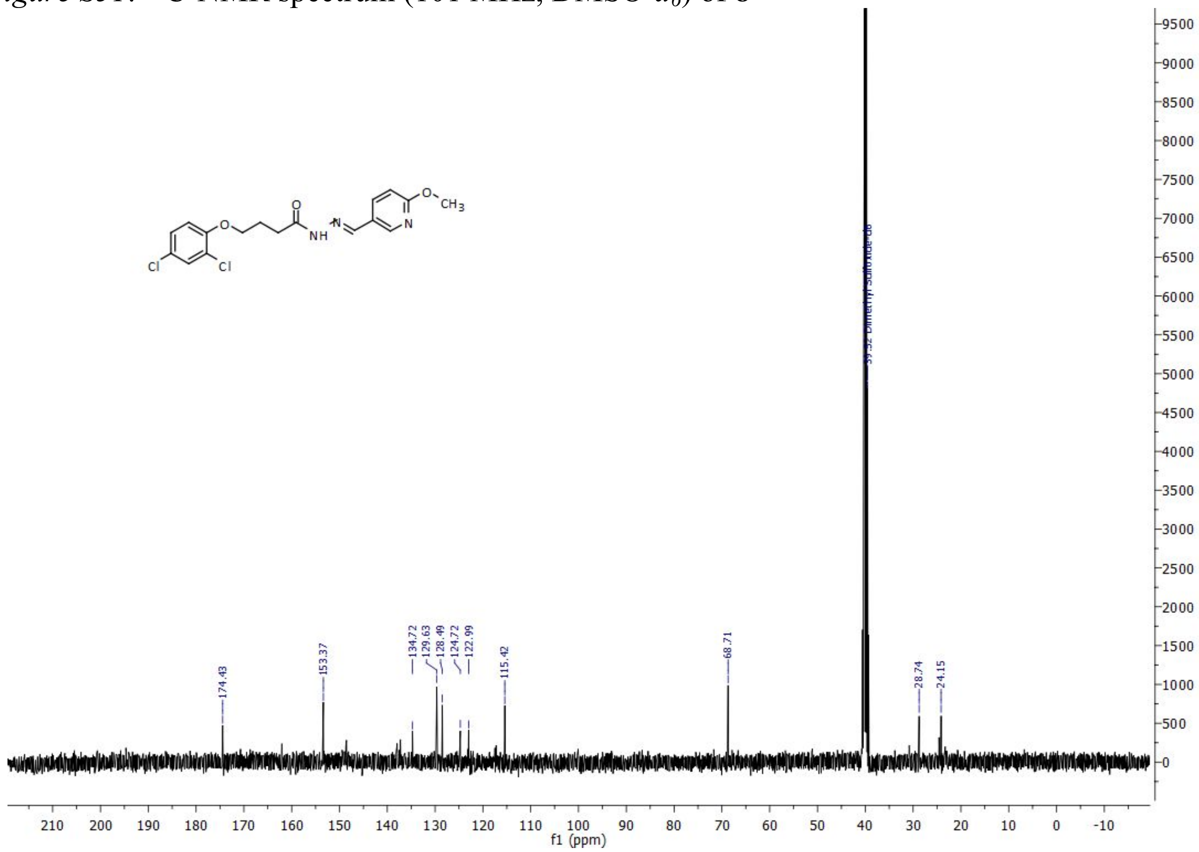

Figure S52. ^{1}H-^{1}H COSY-NMR spectrum (400 MHz, DMSO-d_6) of **8**

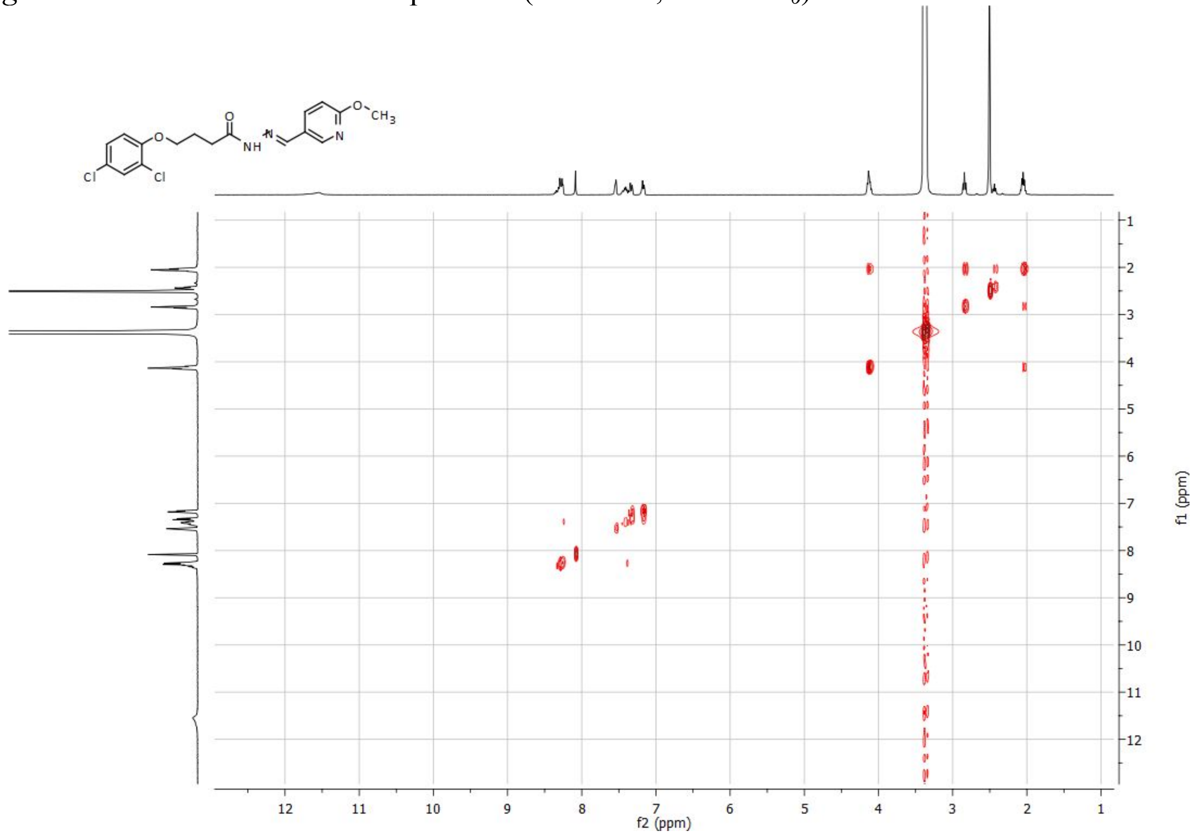

Figure S53. ^{1}H-^{13}C HSQC-NMR spectrum (400 MHz, DMSO-d_6) of **8**

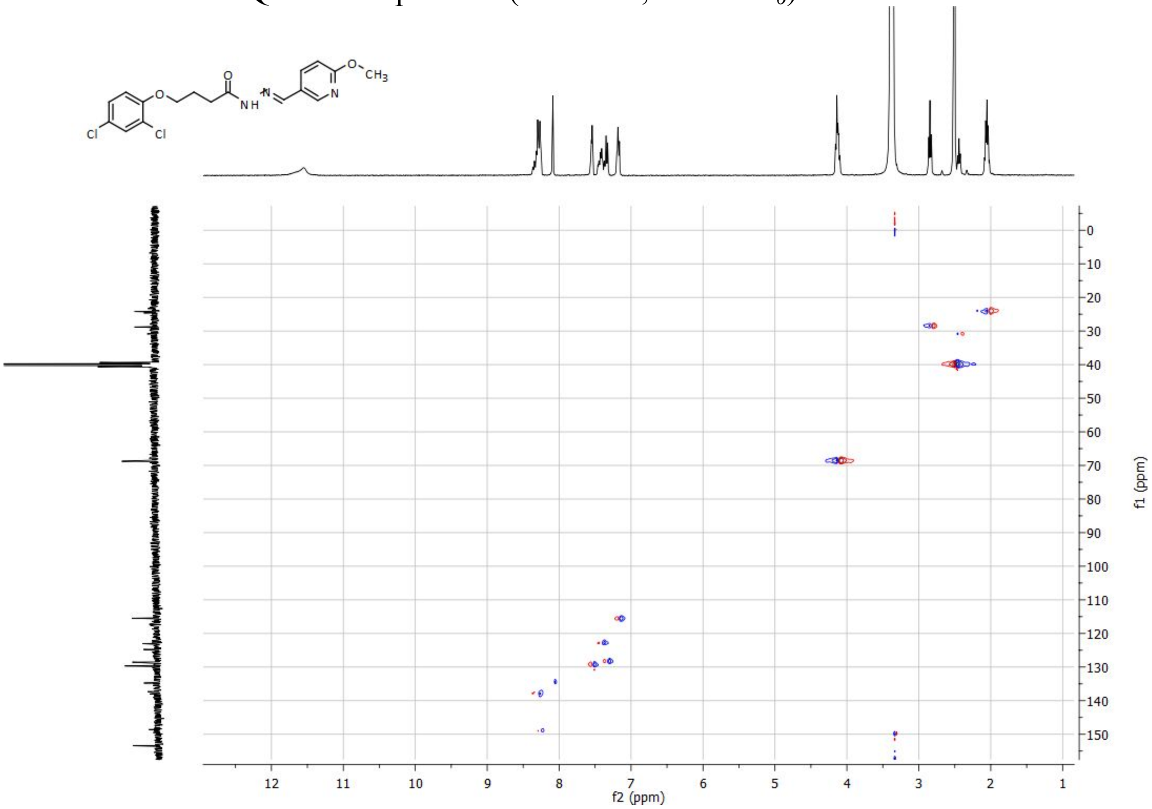

Figure S54. UPLC-MS analysis of **8**

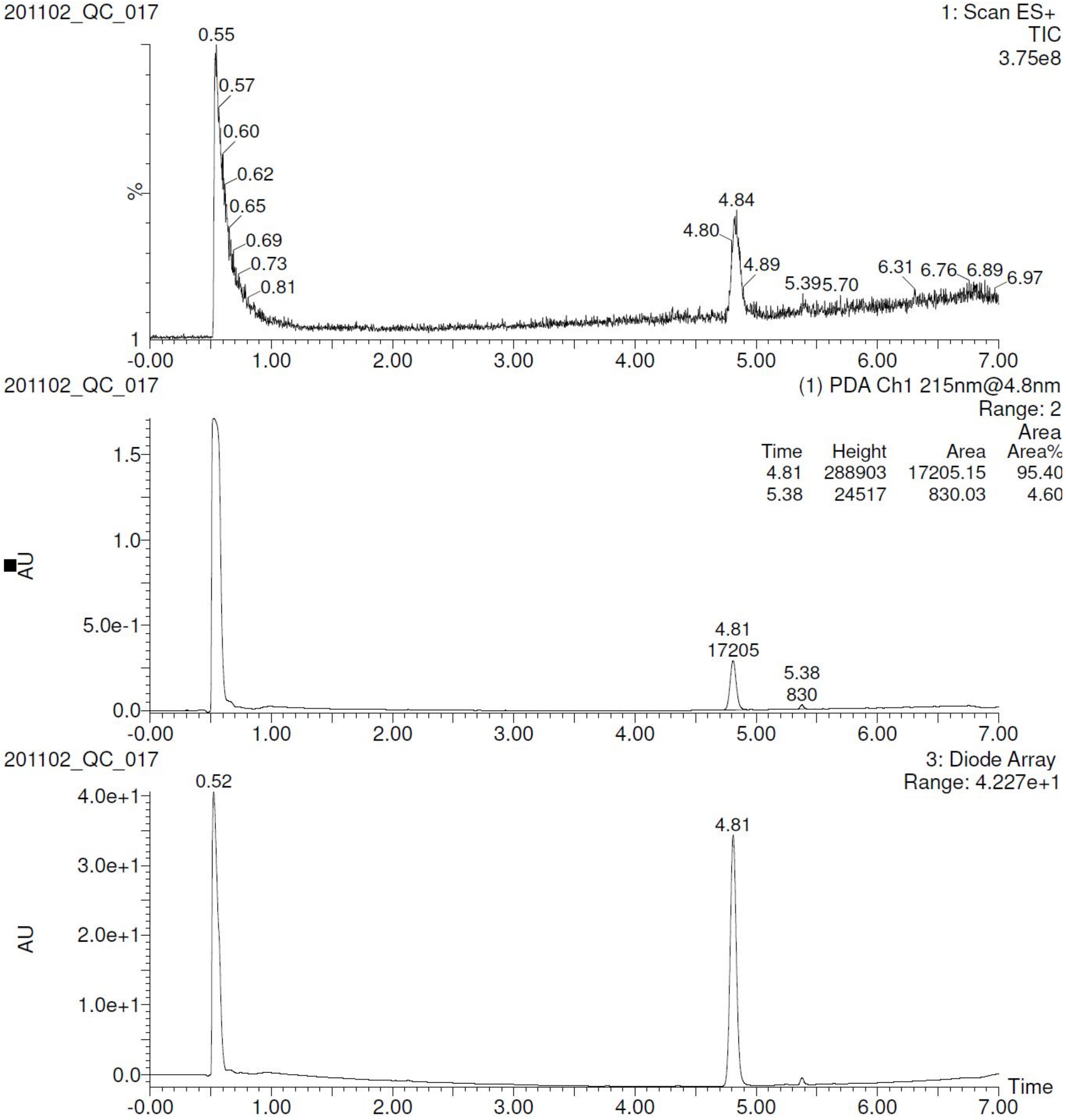

Figure S55. ^{1}H-NMR spectrum (400 MHz, DMSO-d_6) of **9**

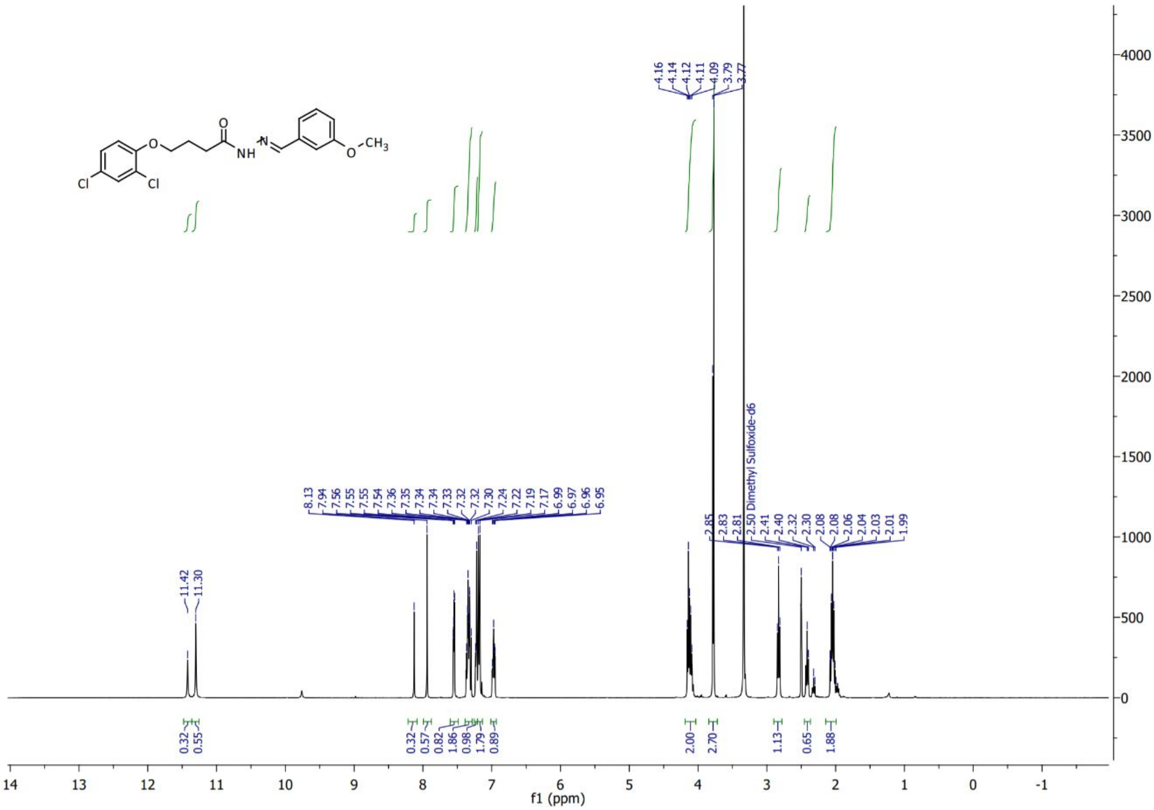

Figure S56. ^{13}C-NMR spectrum (101 MHz, DMSO-d_6) of **9**

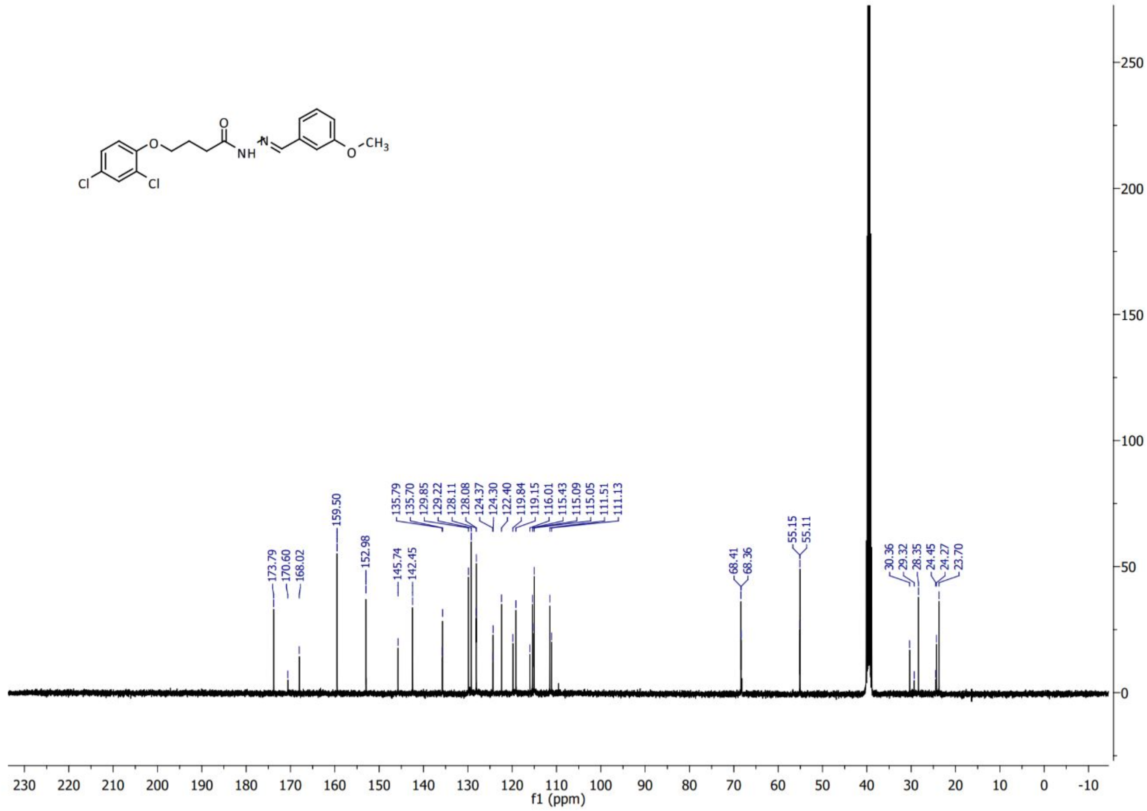

Figure S57. ^{1}H-^{1}H COSY-NMR spectrum (400 MHz, DMSO-d_6) of **9**

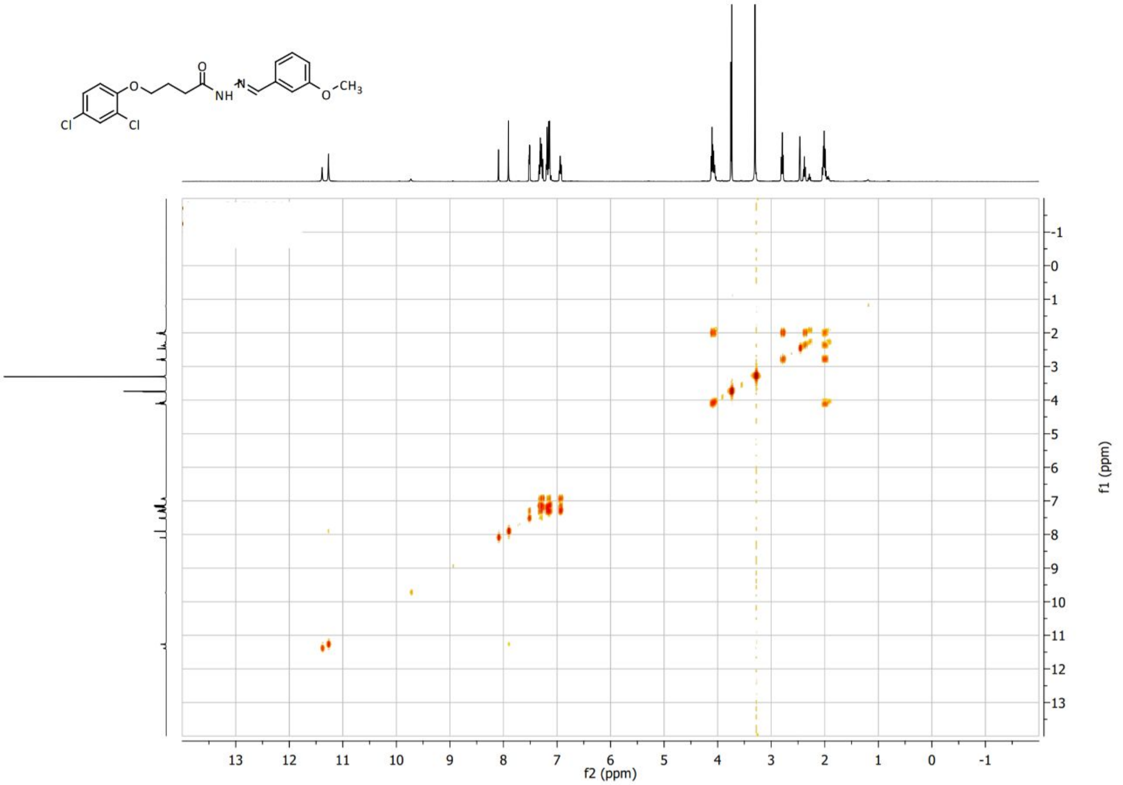

Figure S58. ^{1}H-^{13}C HSQC-NMR spectrum (400 MHz, DMSO-d_6) of **9**

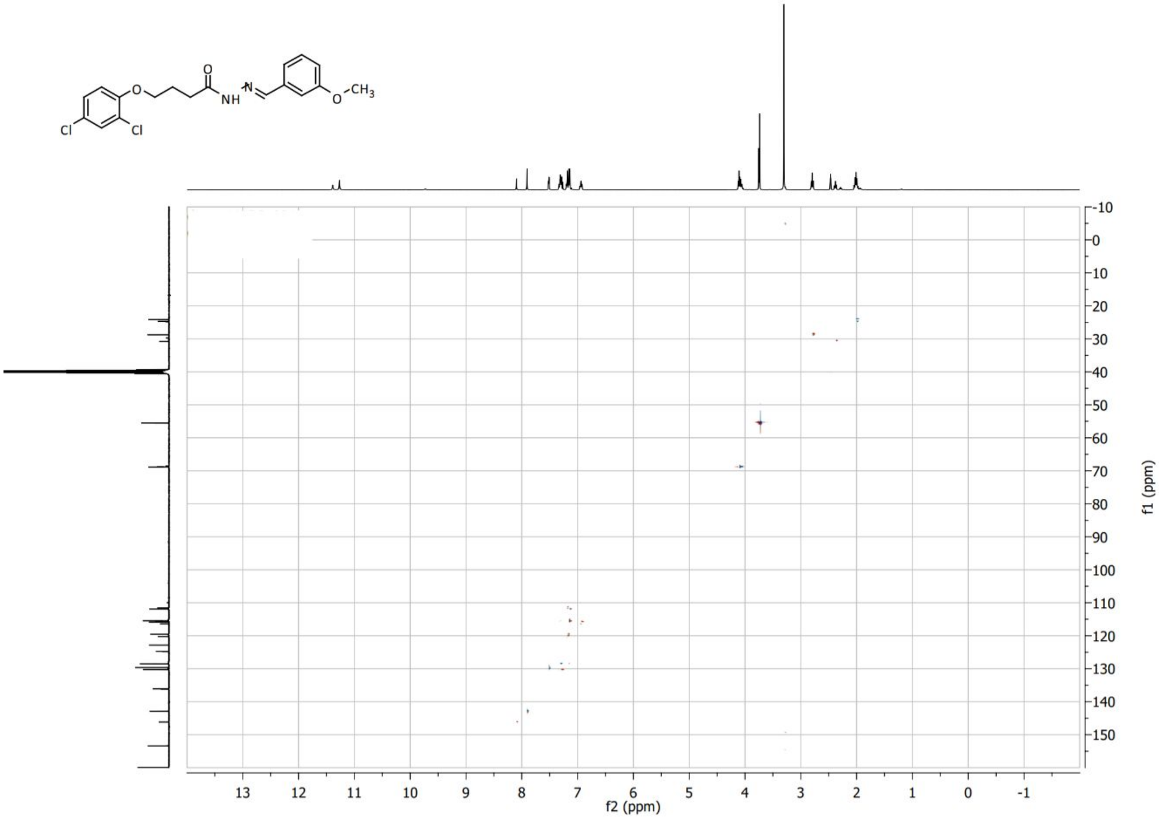

Figure S59. UPLC-MS analysis of **9**

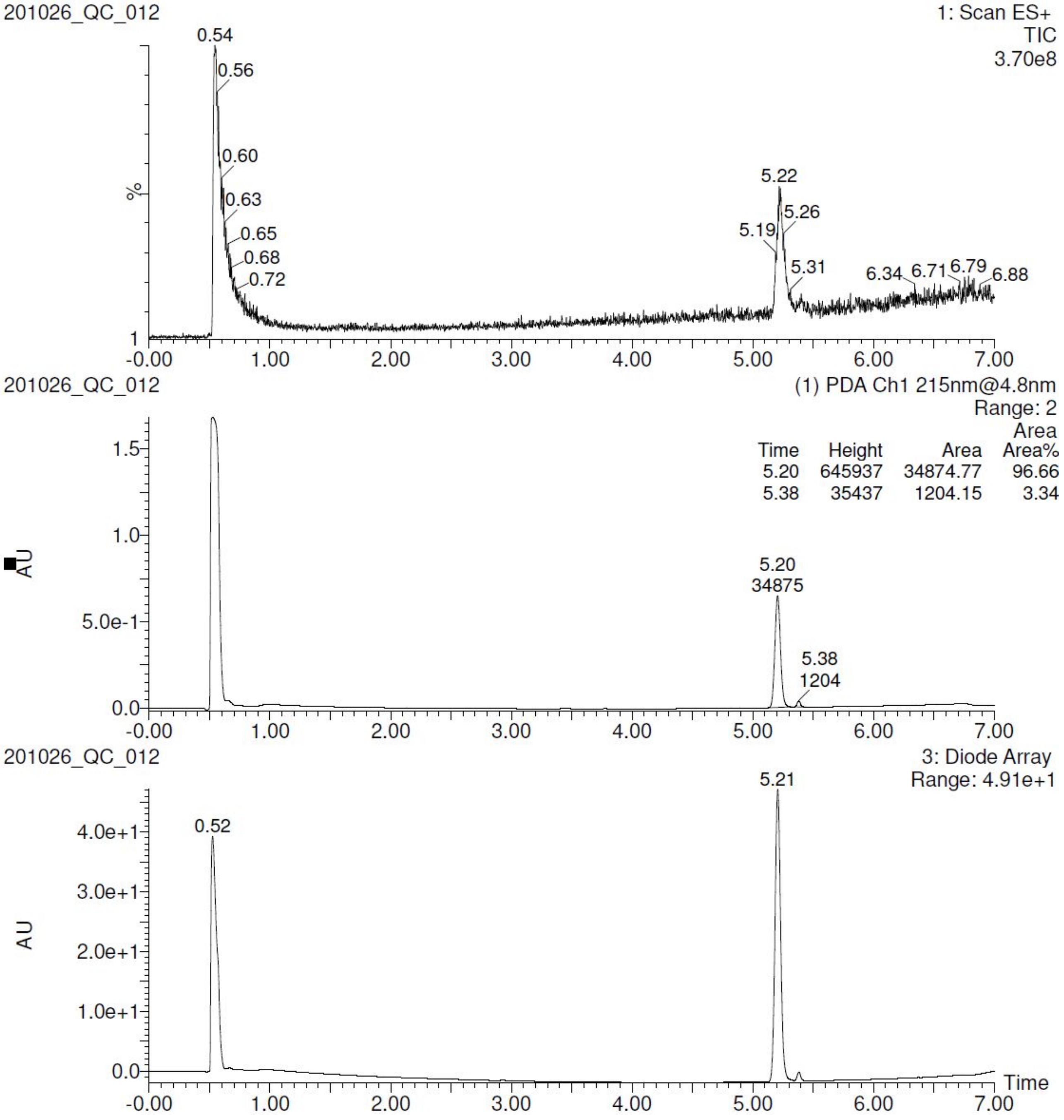

Figure S60. ^{1}H-NMR spectrum (400 MHz, DMSO-d_6) of **10**

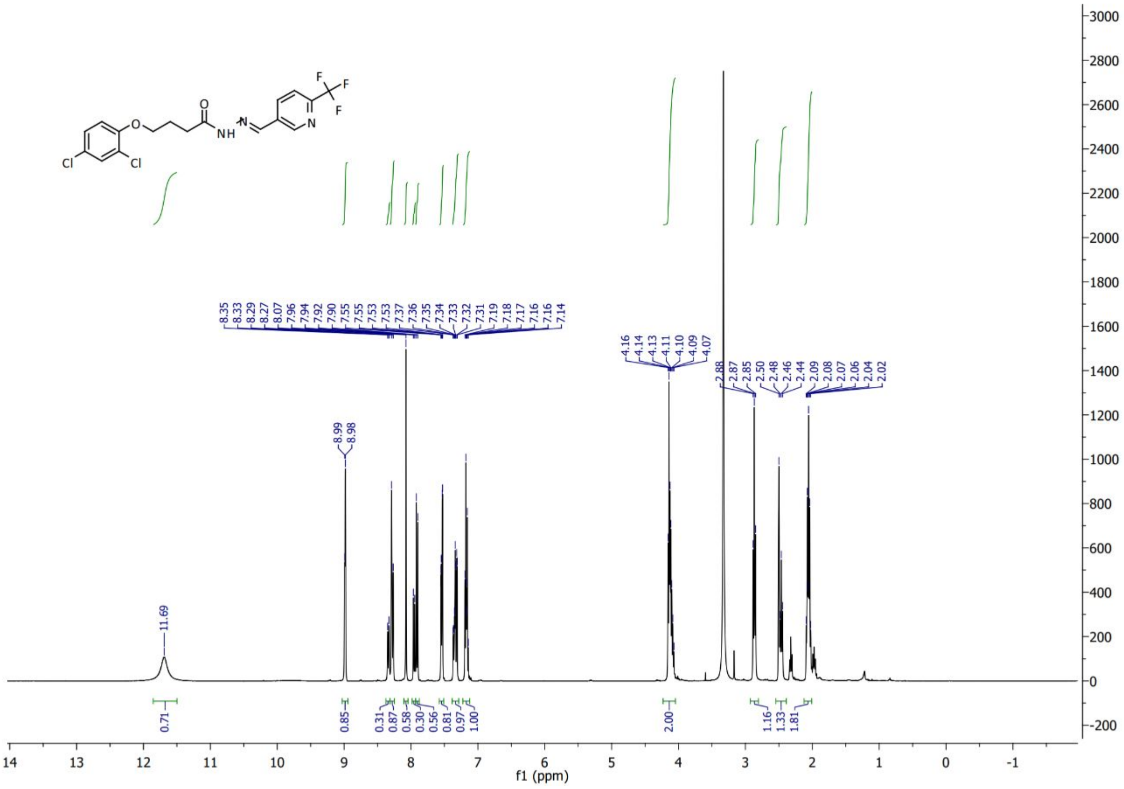

Figure S61. ^{13}C-NMR spectrum (101 MHz, DMSO-d_6) of **10**

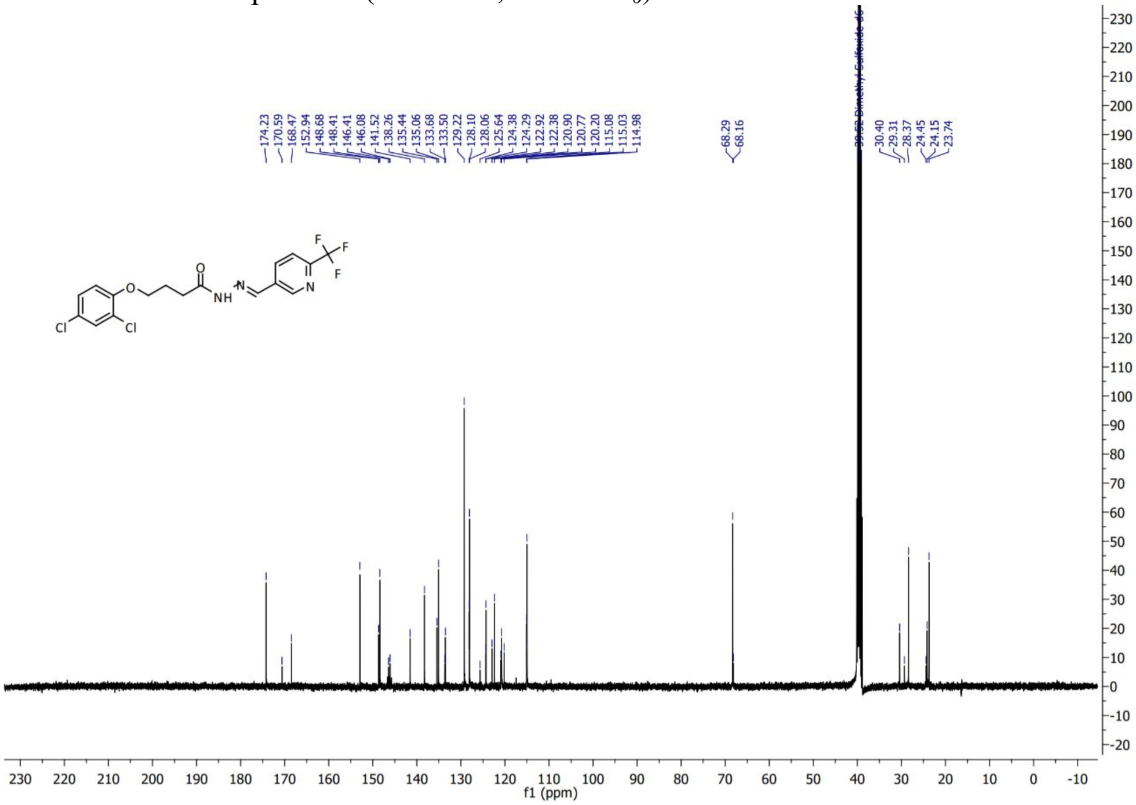

Figure S62. ^{1}H-^{1}H COSY-NMR spectrum (400 MHz, DMSO-d_6) of **10**

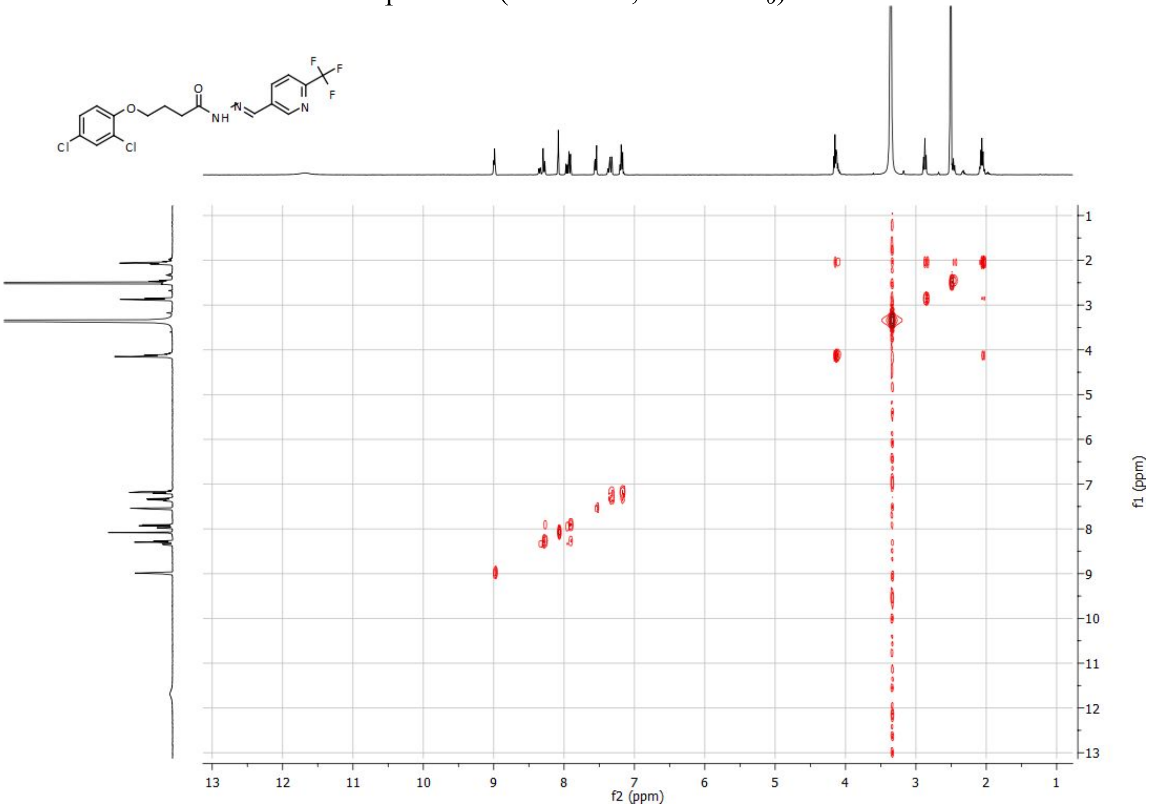

Figure S63. ^{1}H-^{13}C HSQC-NMR spectrum (400 MHz, DMSO-d_6) of **10**

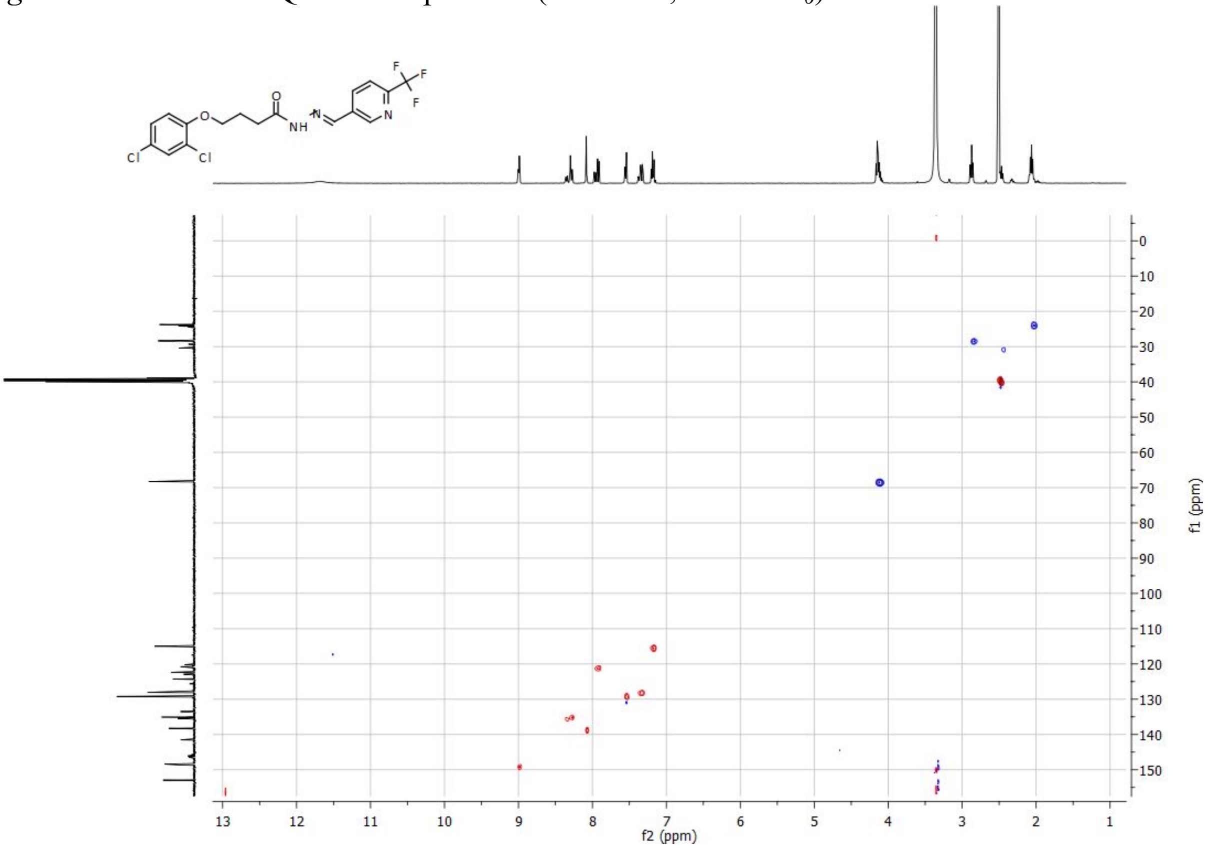

Figure S64. UPLC-MS analysis of **10**

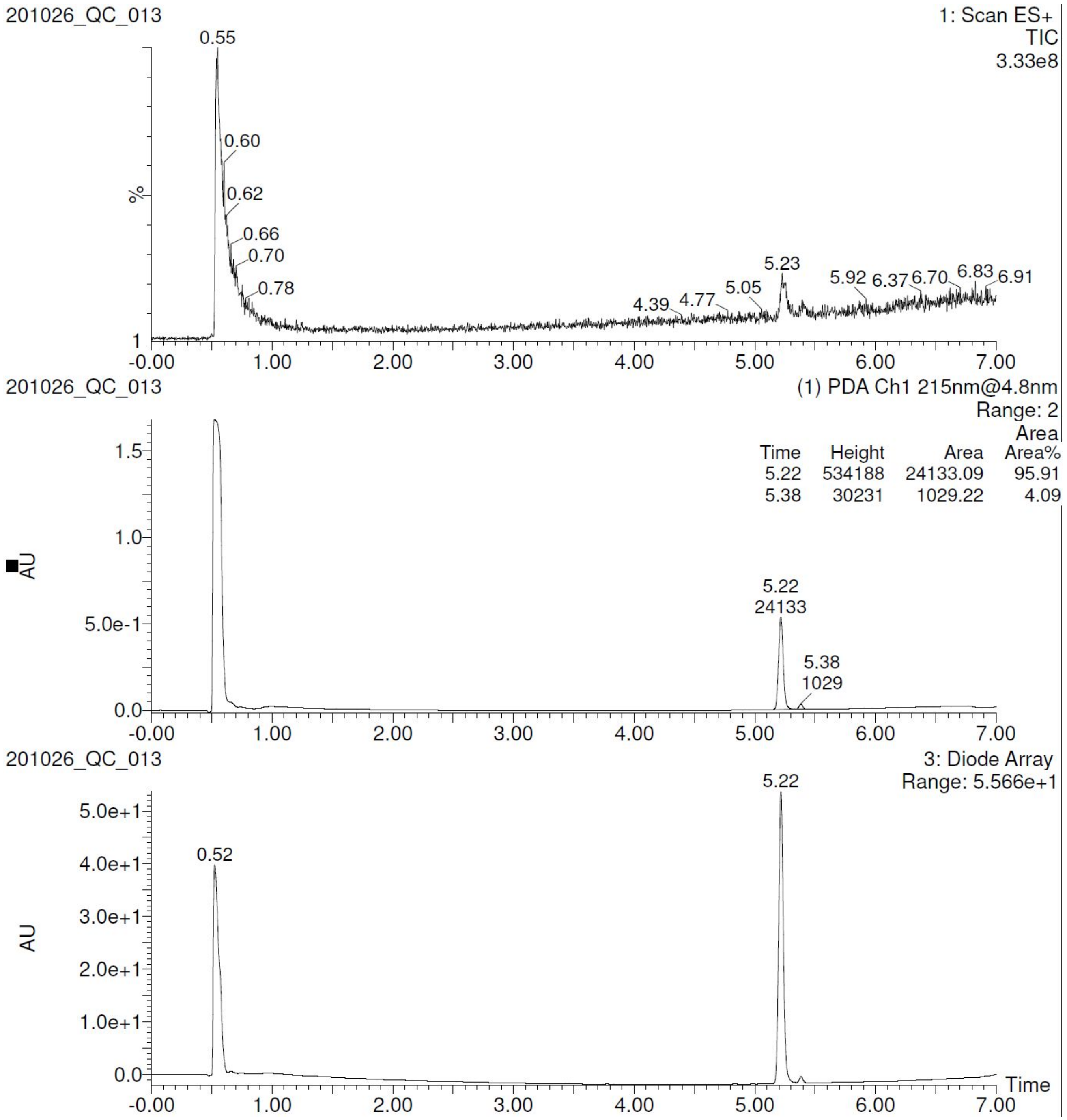

Time	Height	Area	Area%
5.22	534188	24133.09	95.91
5.38	30231	1029.22	4.09

Figure S65. ^{1}H-NMR spectrum (400 MHz, DMSO-d_6) of **11**

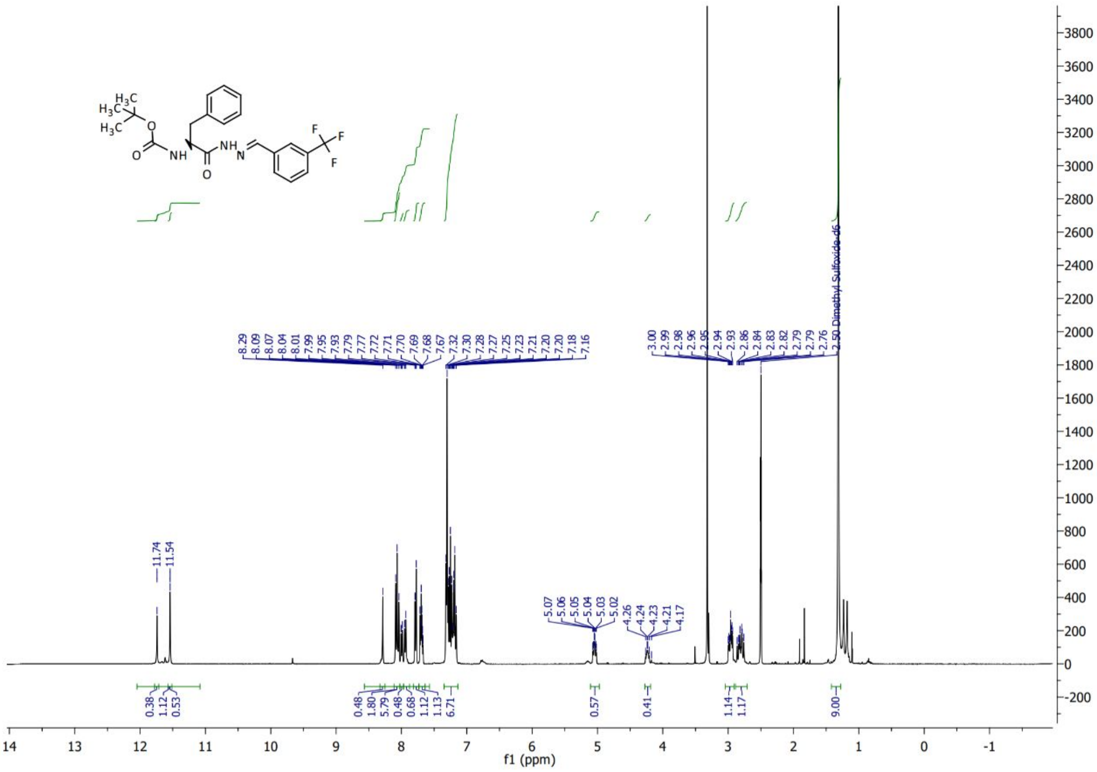

Figure S66. ^{13}C-NMR spectrum (101 MHz, DMSO-d_6) of **11**

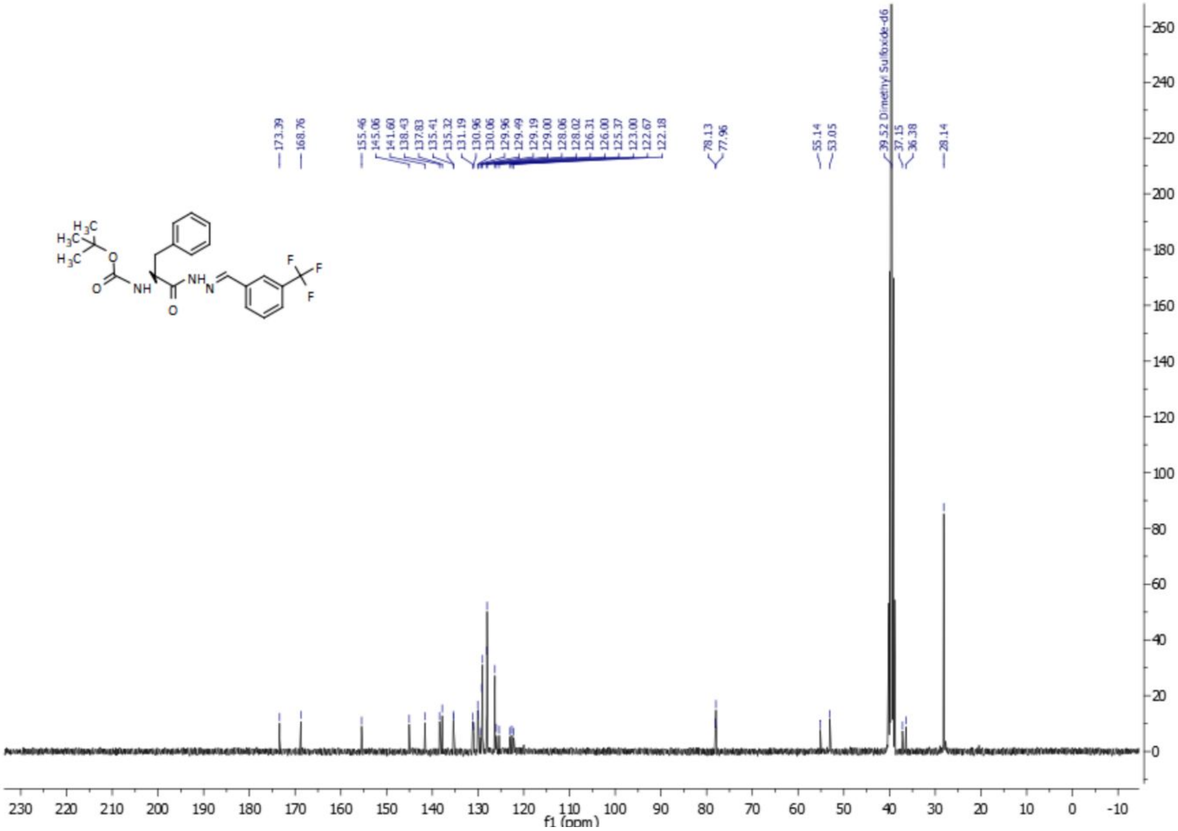

Figure S67. ^{1}H-^{1}H COSY-NMR spectrum (400 MHz, DMSO-d_6) of **11**

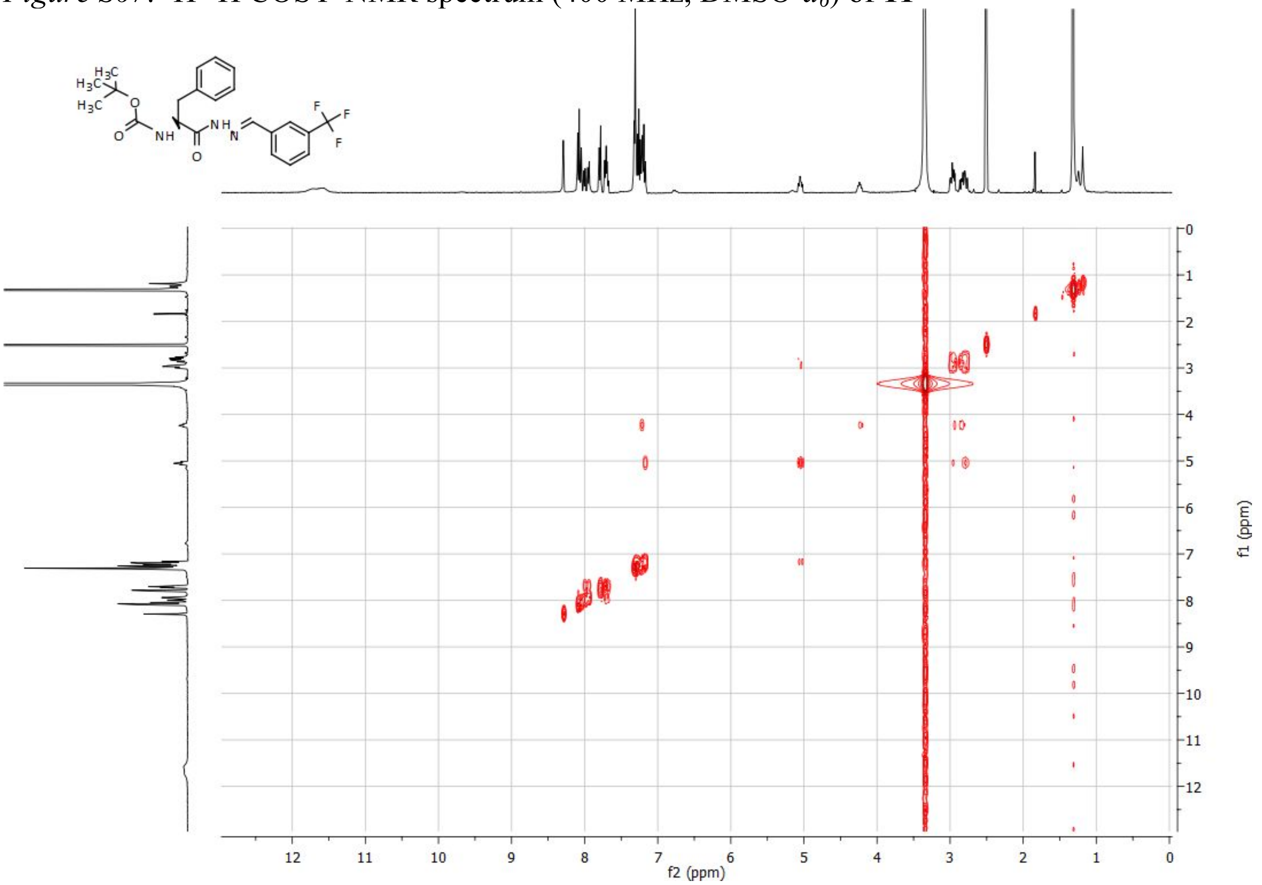

Figure S68. ^{1}H-^{13}C HSQC-NMR spectrum (400 MHz, DMSO-d_6) of **11**

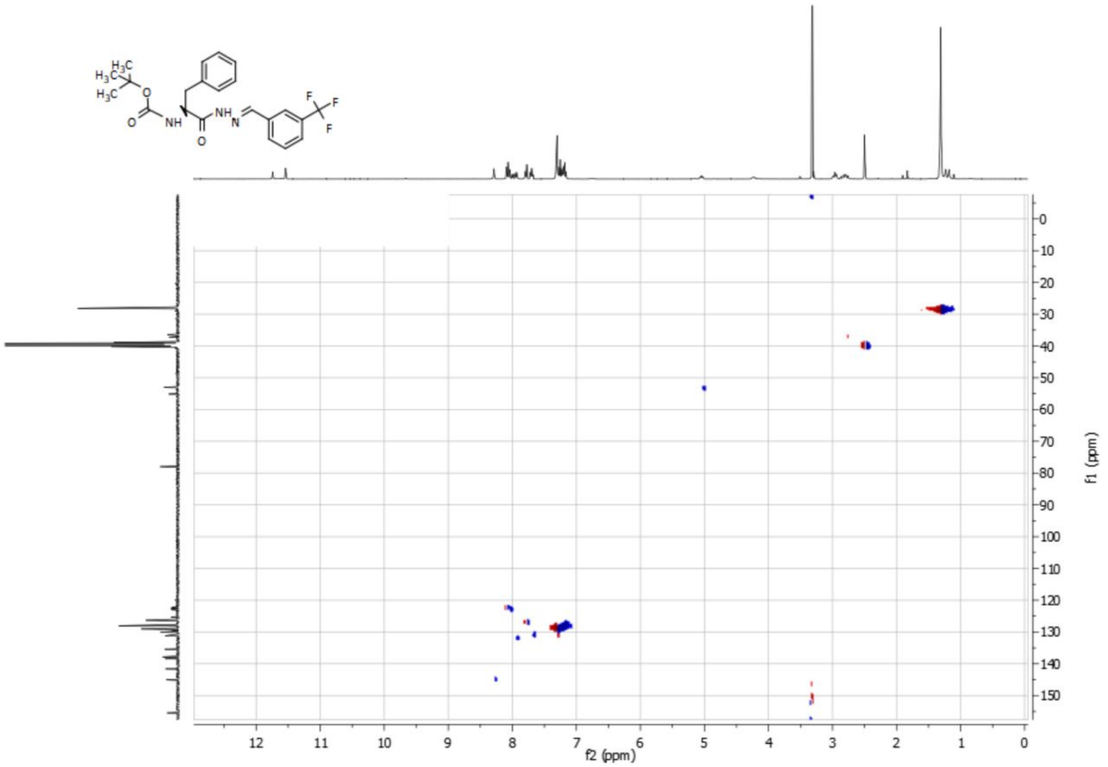

Figure S69. UPLC-MS analysis of **11**

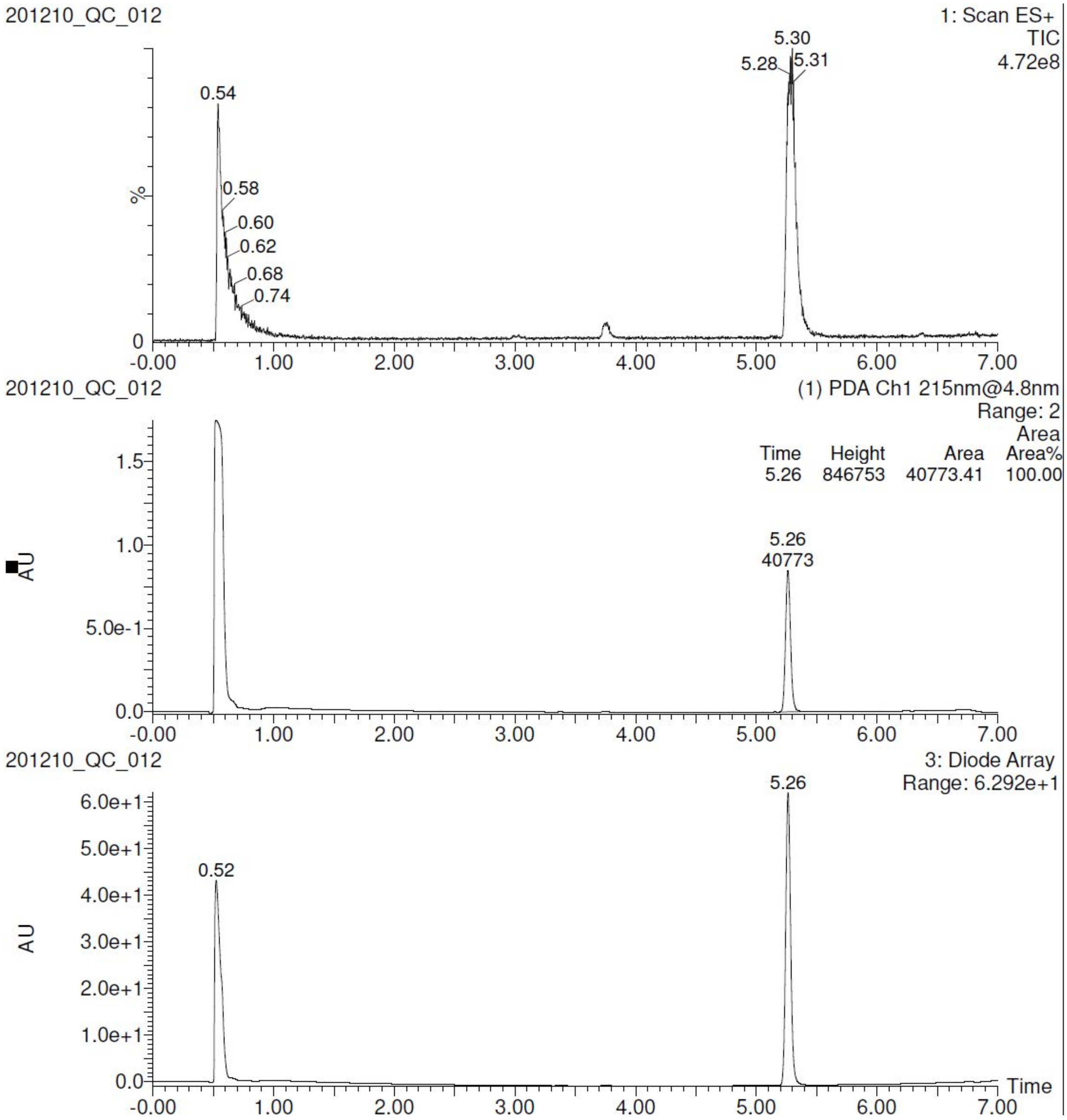

Figure S70. ^{1}H-NMR spectrum (400 MHz, DMSO-d_6) of **12**

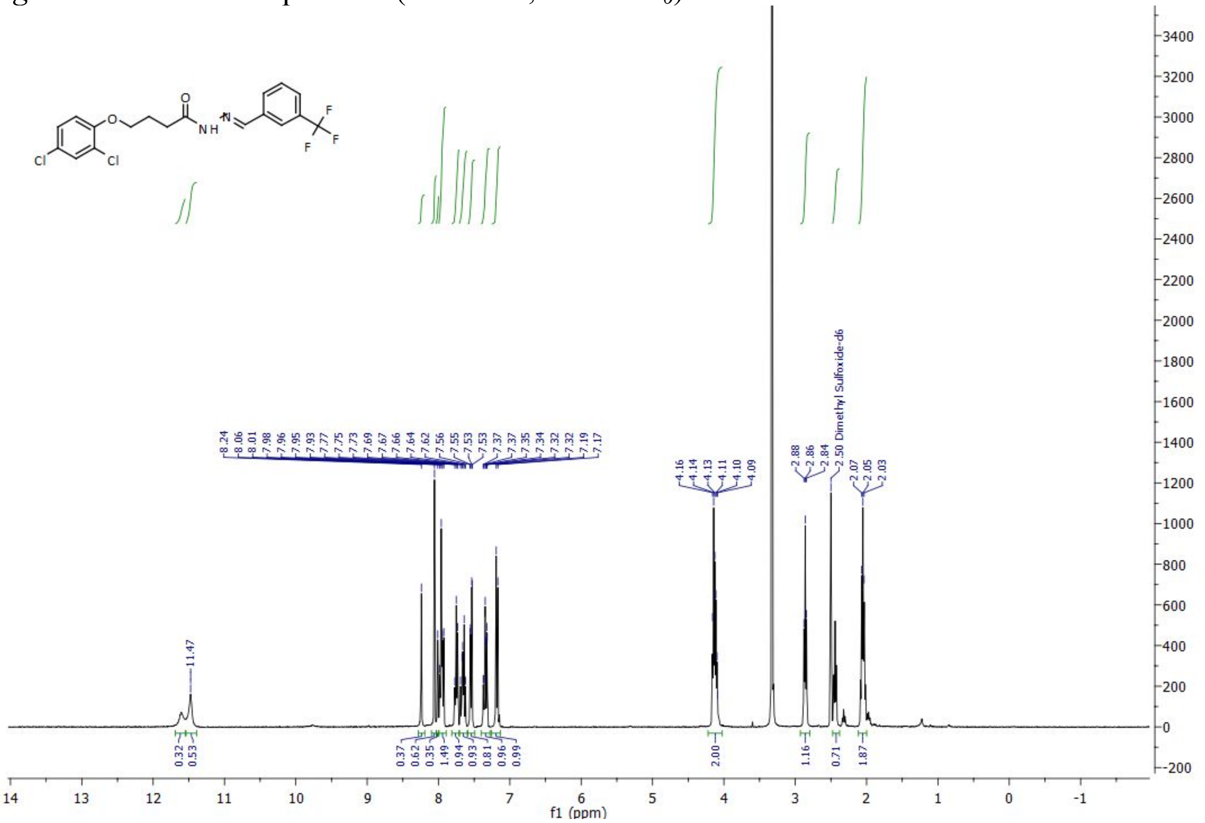

Figure S71. ^{13}C-NMR spectrum (101 MHz, DMSO-d_6) of **12**

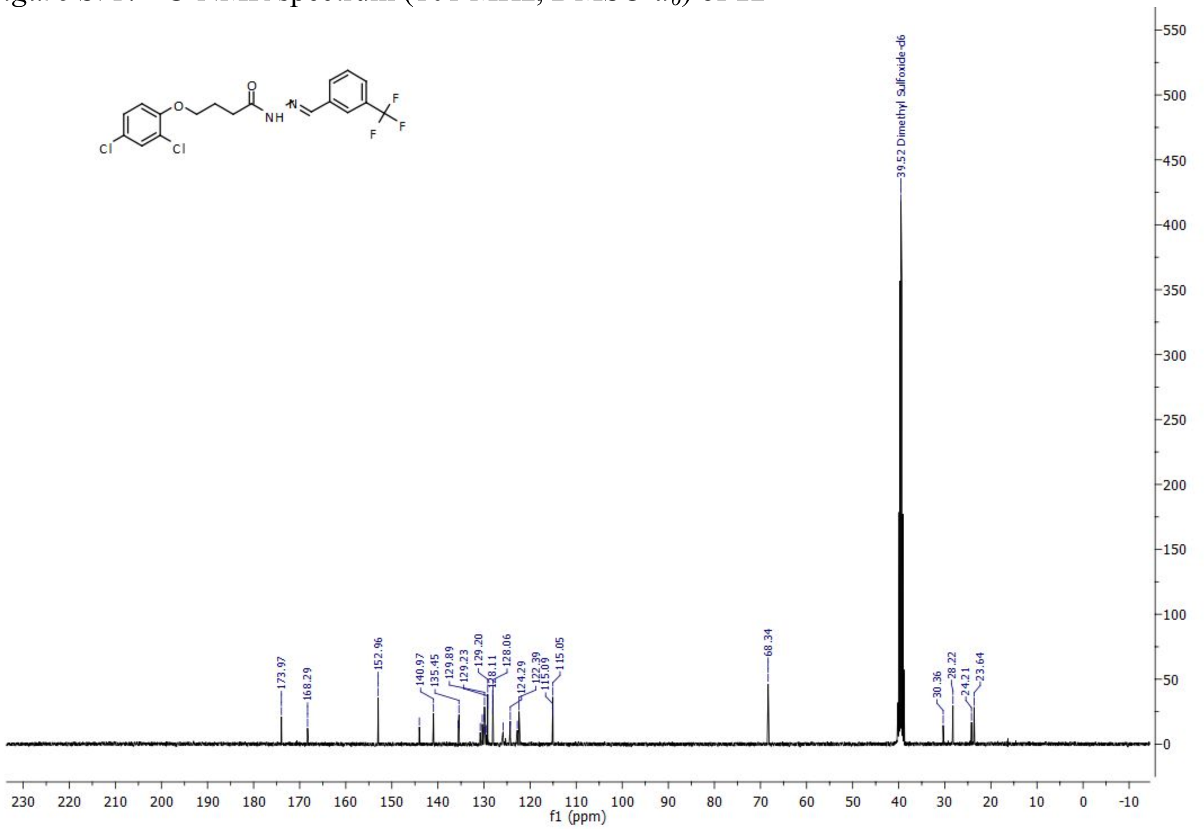

Figure S72. ^{1}H-^{1}H COSY-NMR spectrum (400 MHz, DMSO-d_6) of **12**

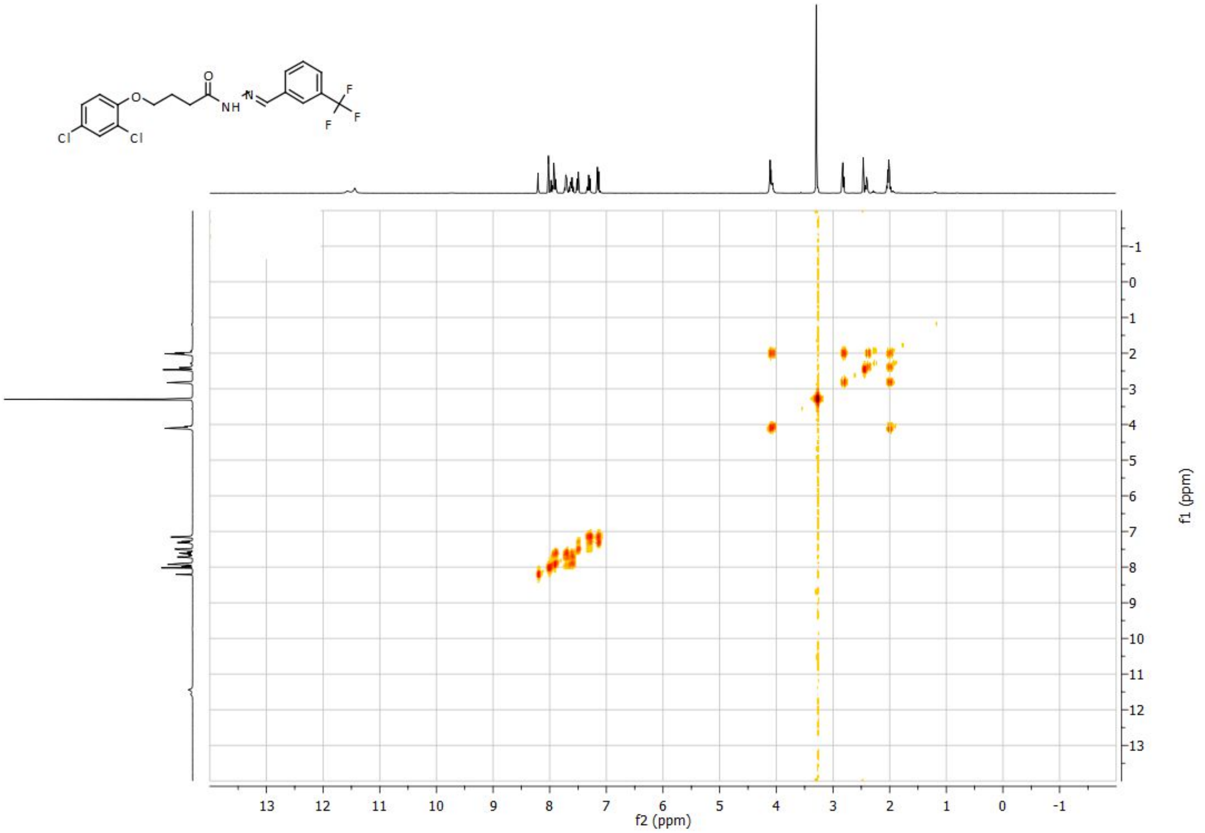

Figure S73. ^{1}H-^{13}C HSQC-NMR spectrum (400 MHz, DMSO-d_6) of **12**

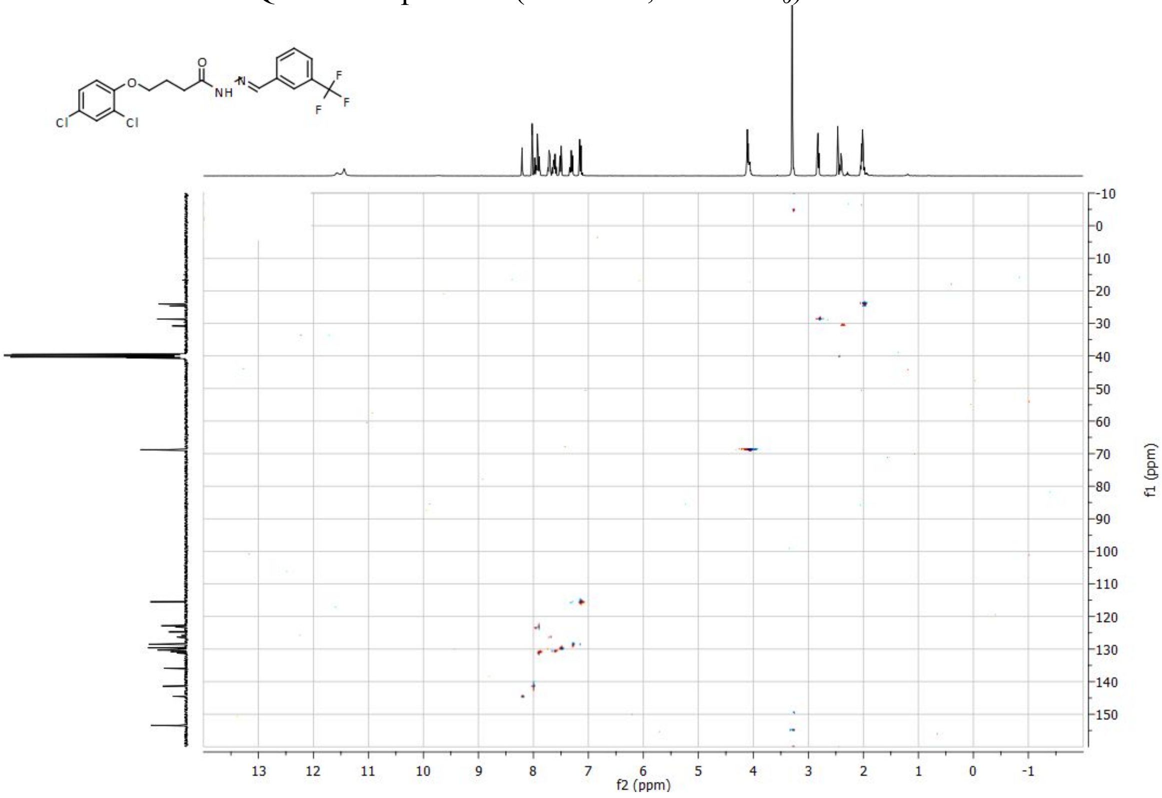

Figure S74. UPLC-MS analysis of **12**

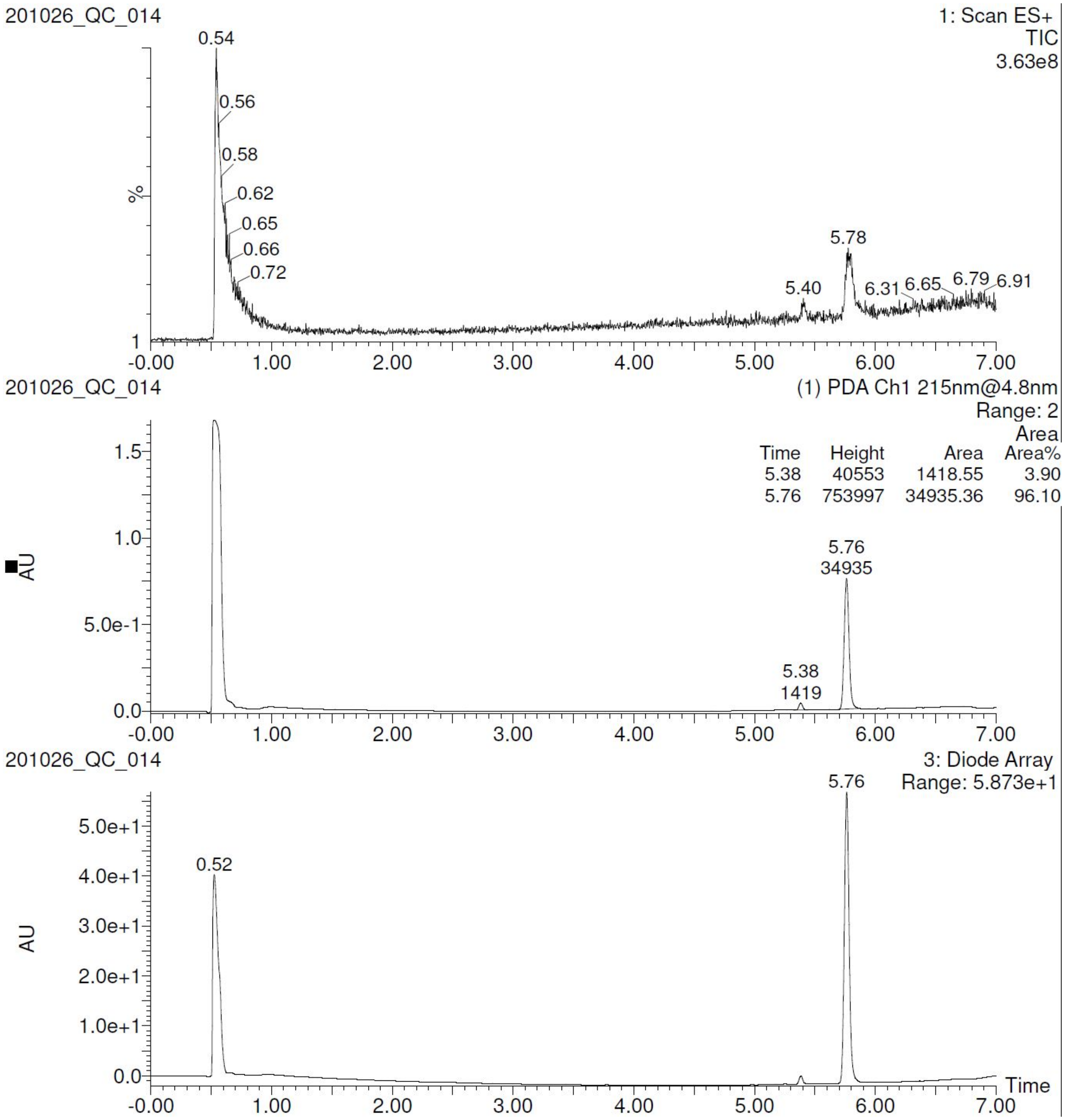

Figure S75. ^{1}H-NMR spectrum (400 MHz, DMSO-d_6) of **13**

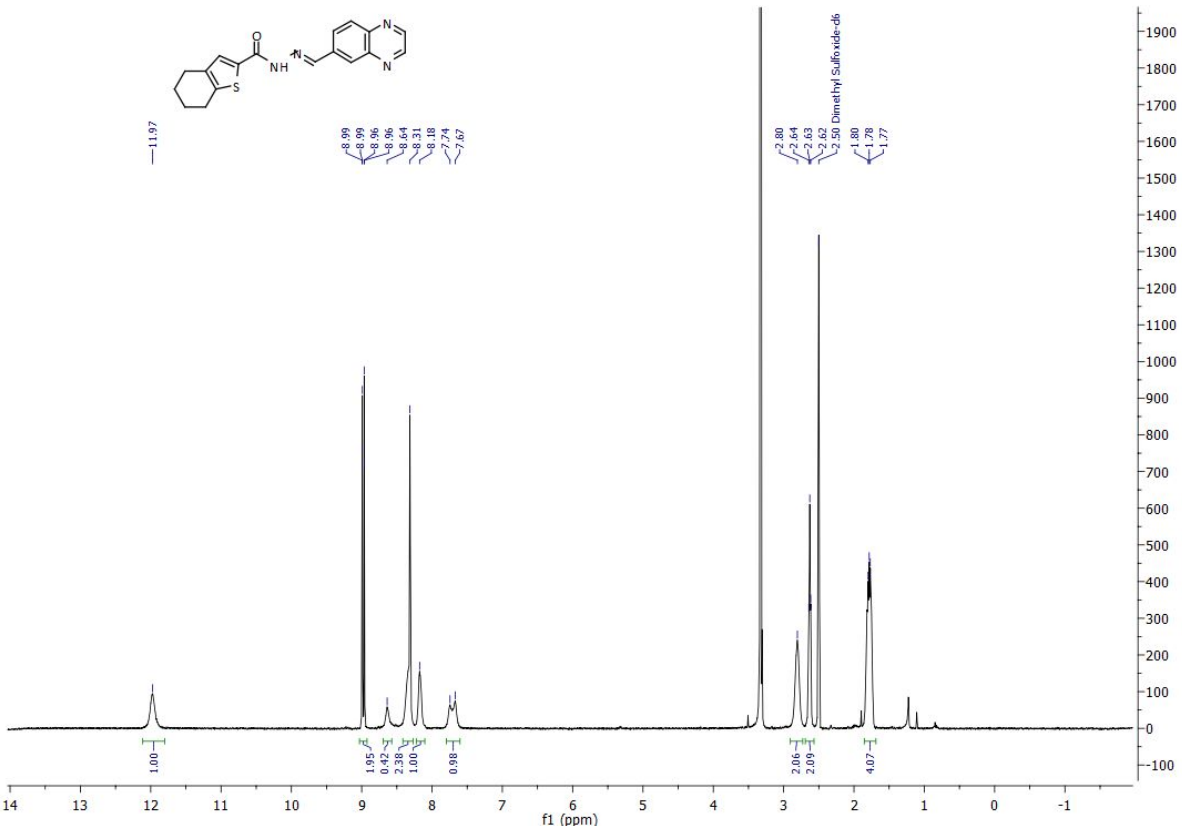

Figure S76. ^{13}C-NMR spectrum (400 MHz, DMSO-d_6) of **13**

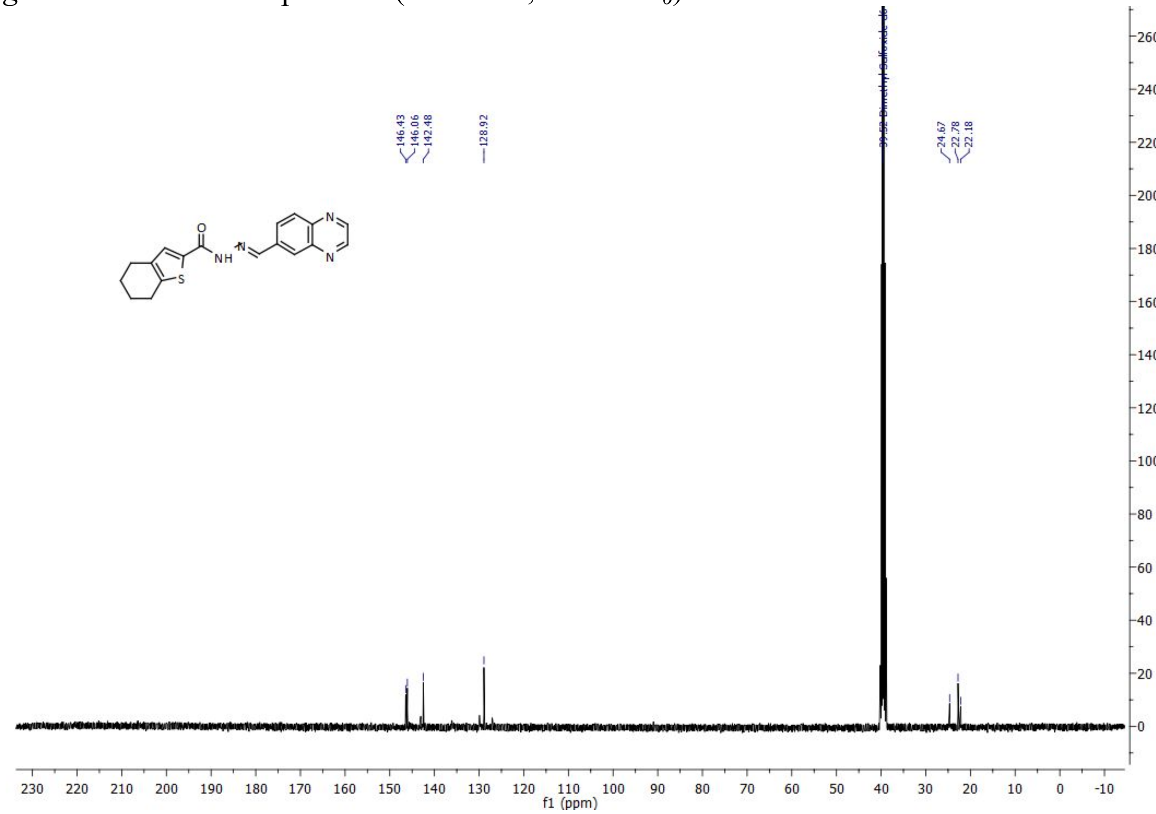

Figure S77. ^{1}H-^{1}H COSY-NMR spectrum (400 MHz, DMSO-d_6) of **13**

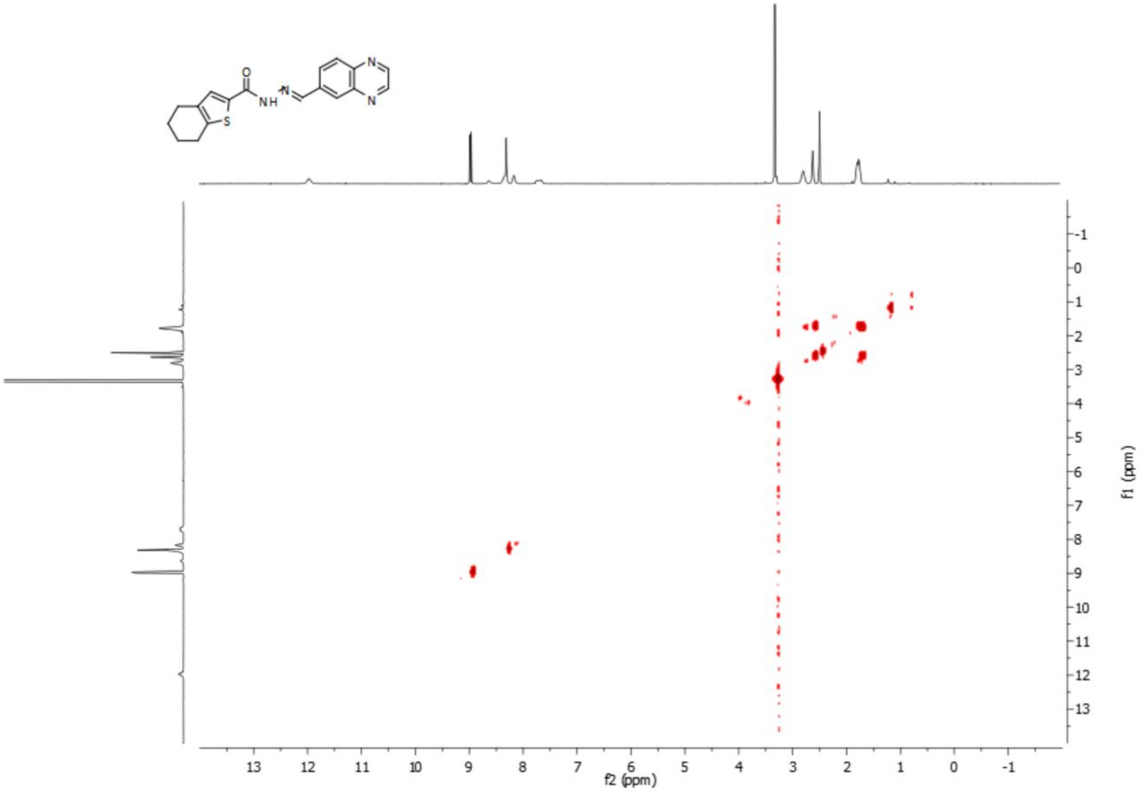

Figure S78. ^{1}H-^{13}C HSQC-NMR spectrum (400 MHz, DMSO-d_6) of **13**

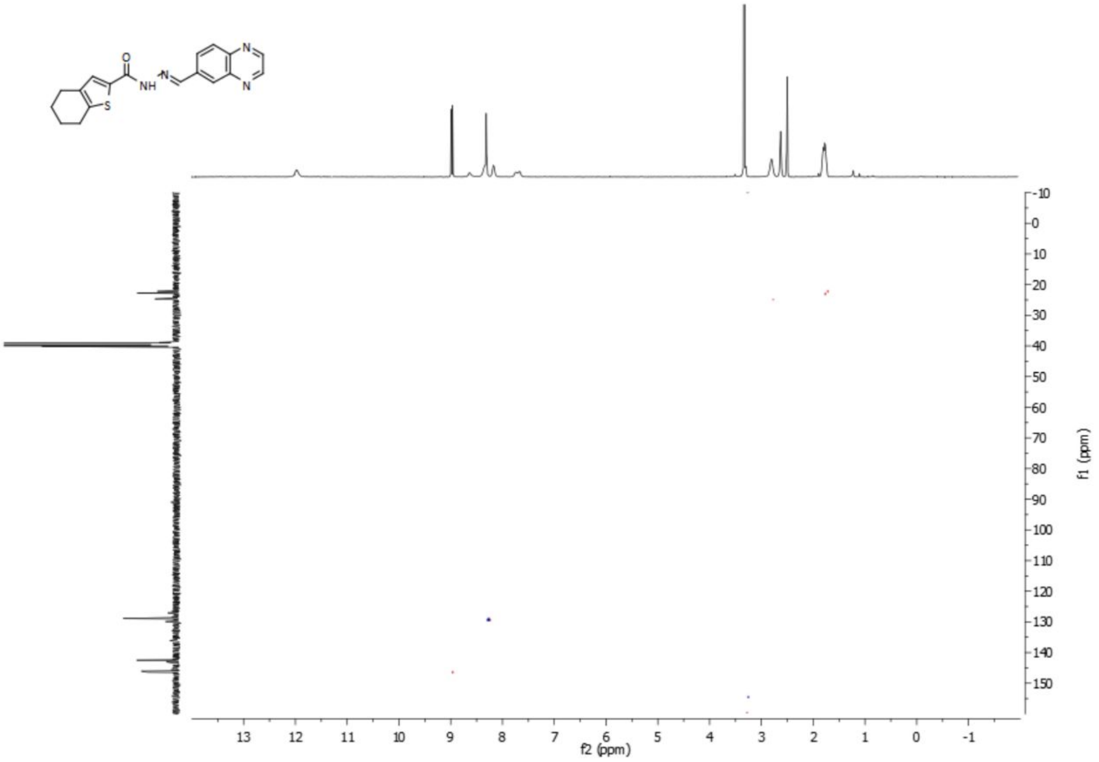

Figure S79. UPLC-MS analysis of **13**

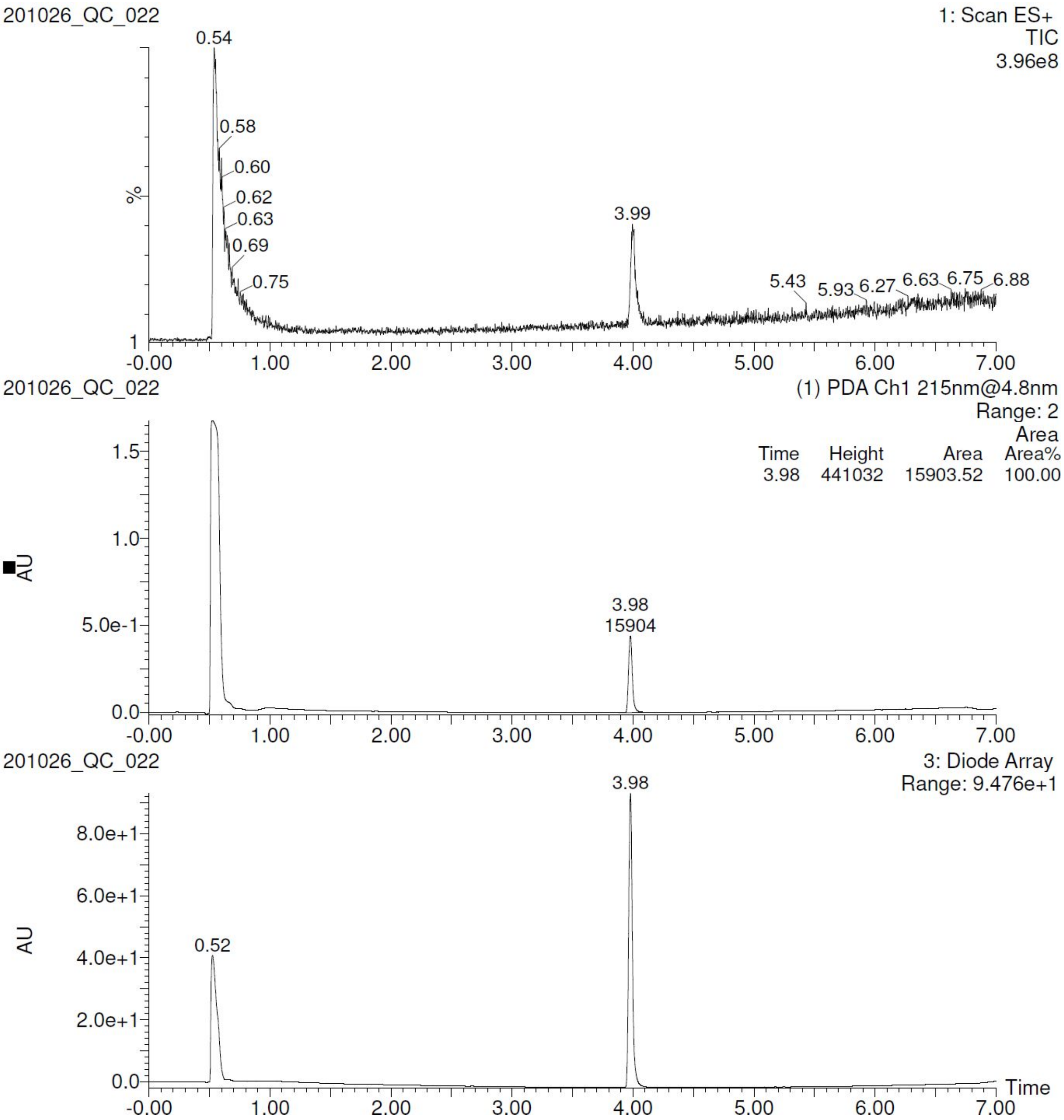

Figure S80. ^{1}H-NMR spectrum (400 MHz, DMSO-d_6) of **14**

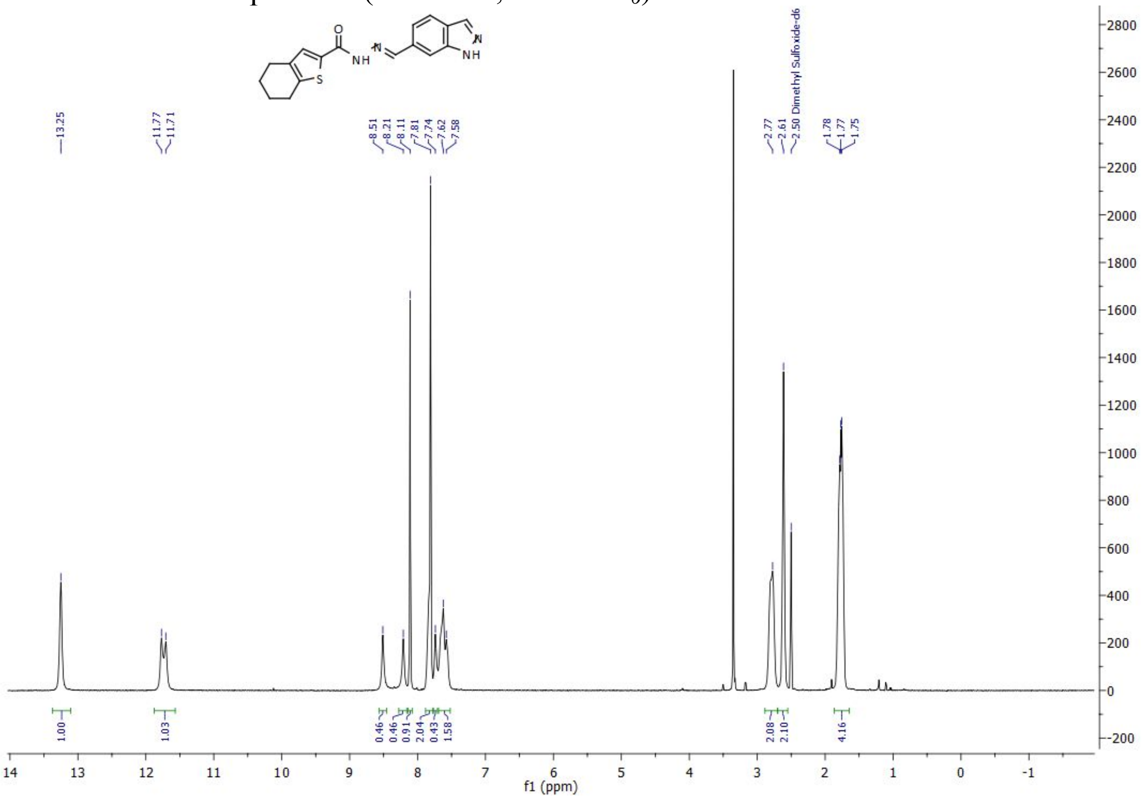

Figure S81. ^{13}C-NMR spectrum (400 MHz, DMSO-d_6) of **14**

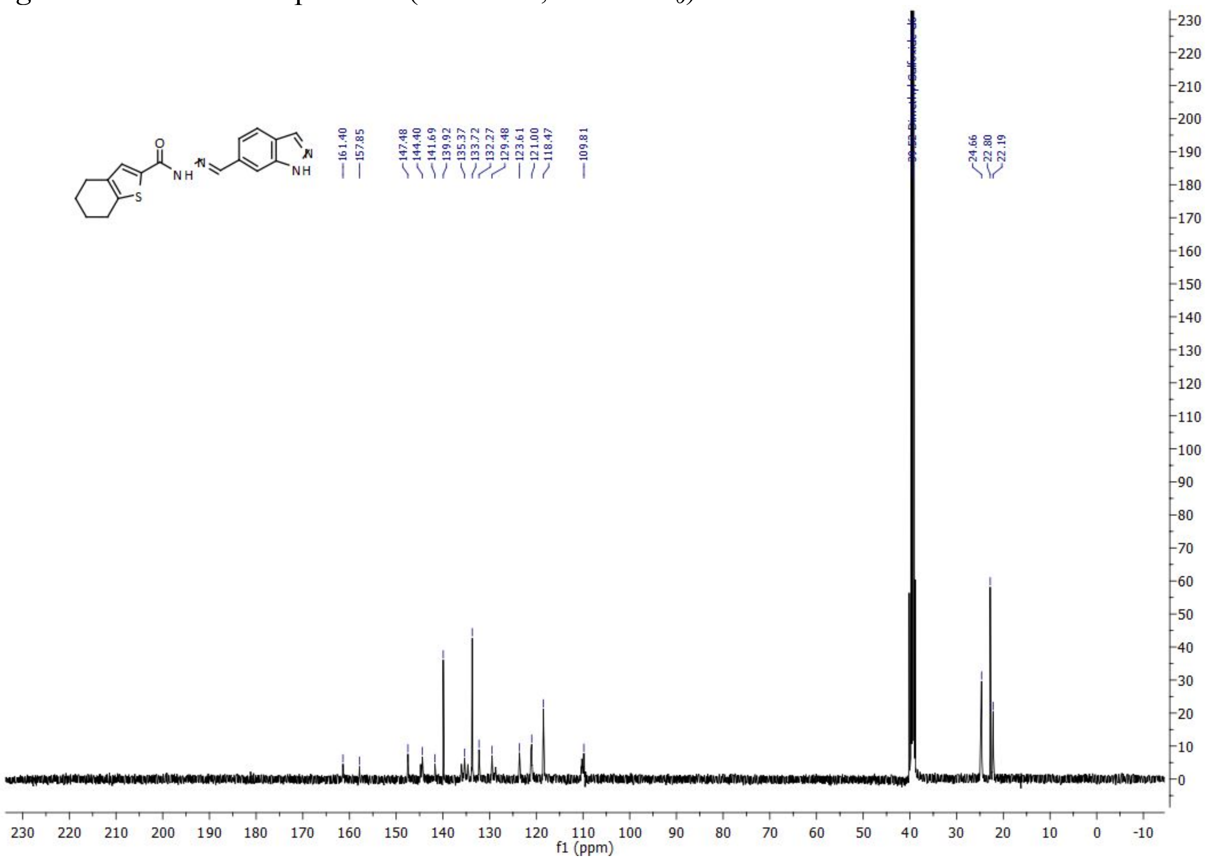

Figure S82. ^{1}H-^{1}H COSY-NMR spectrum (400 MHz, DMSO-d_6) of **14**

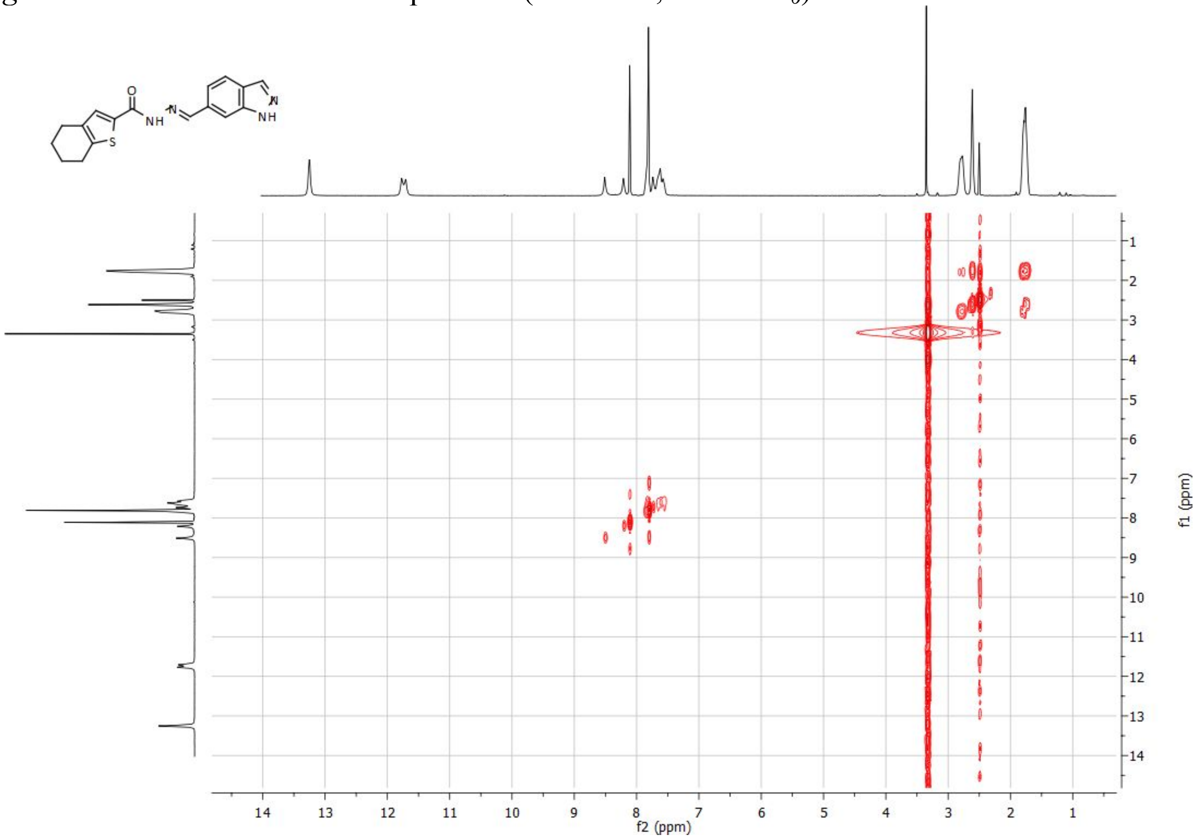

Figure S83. ^{1}H-^{13}C HSQC-NMR spectrum (400 MHz, DMSO-d_6) of **14**

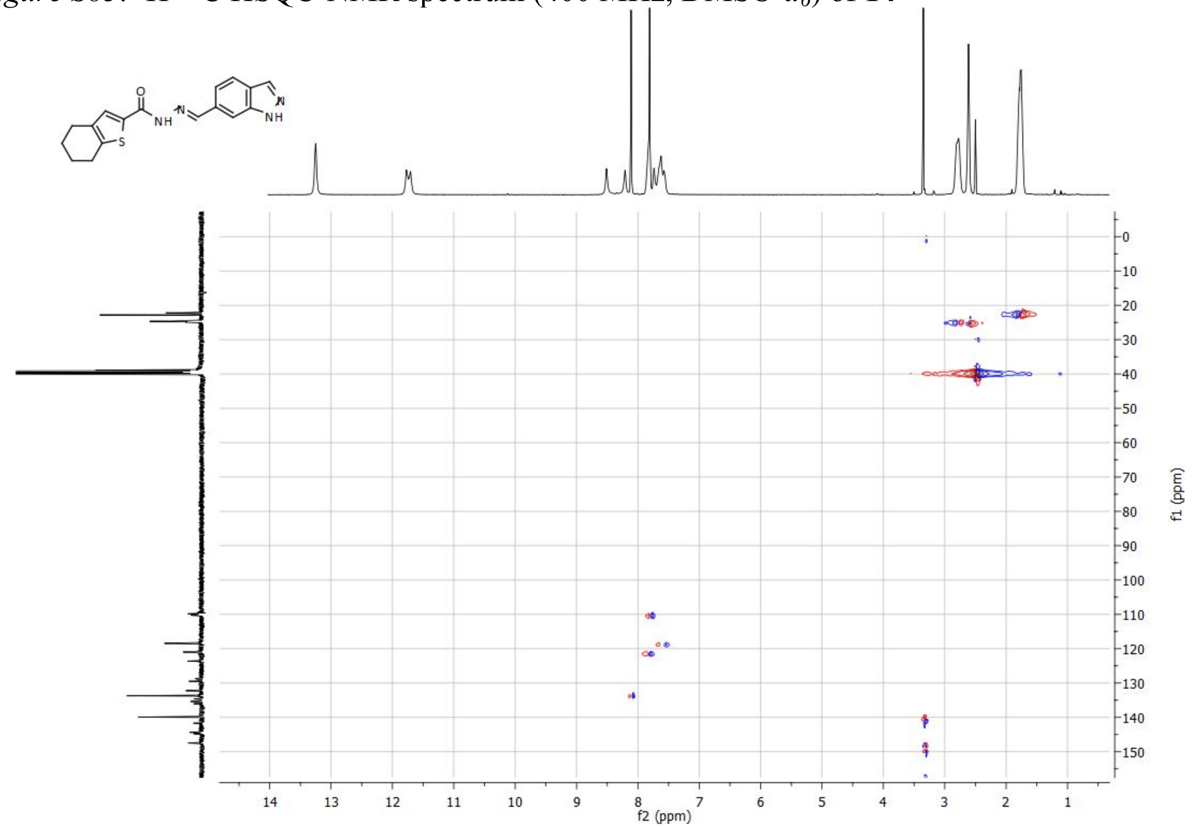

Figure S84. UPLC-MS analysis of **14**

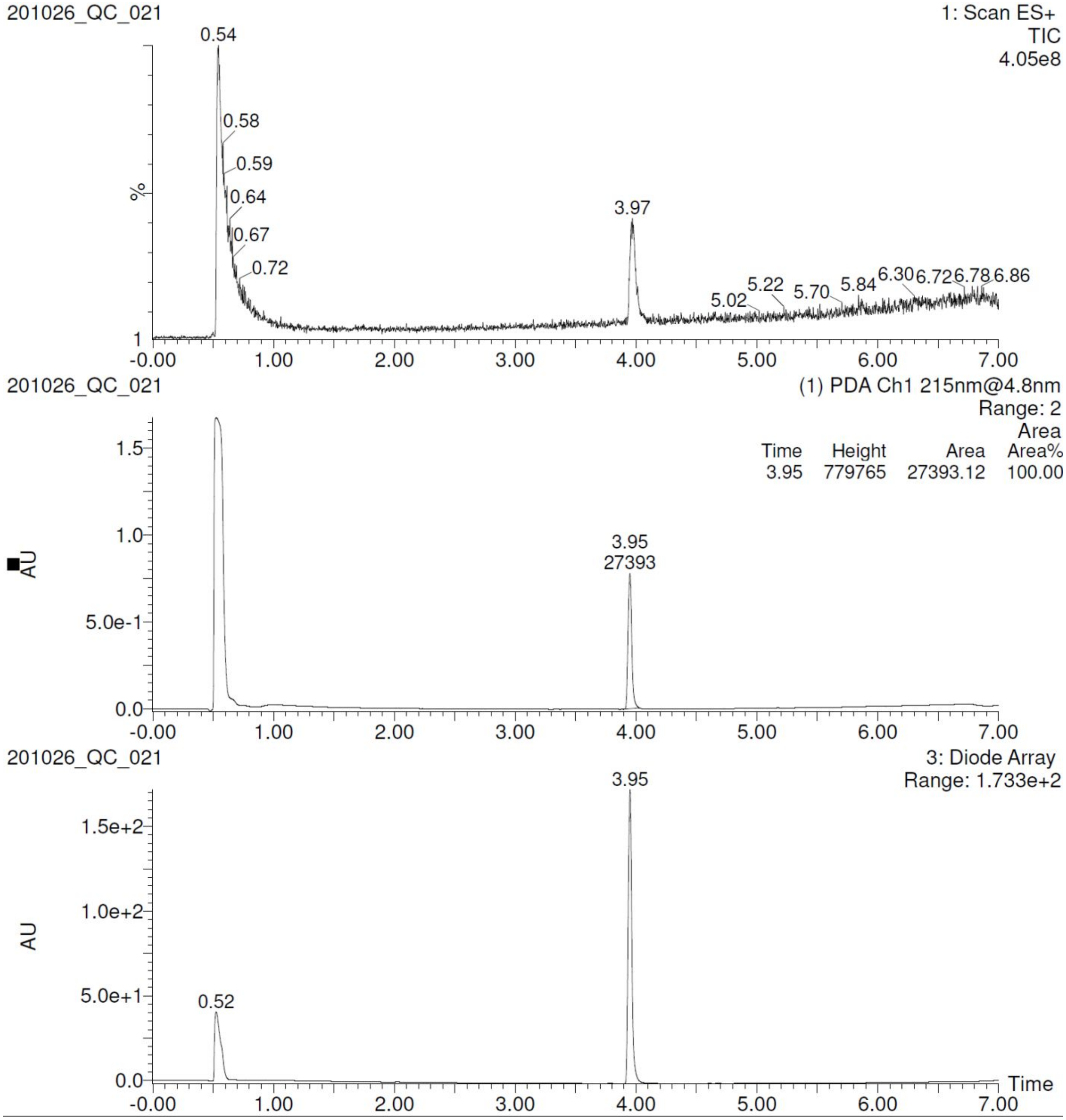

Figure S85. ^{1}H-NMR spectrum (400 MHz, DMSO-d_6) of **15**

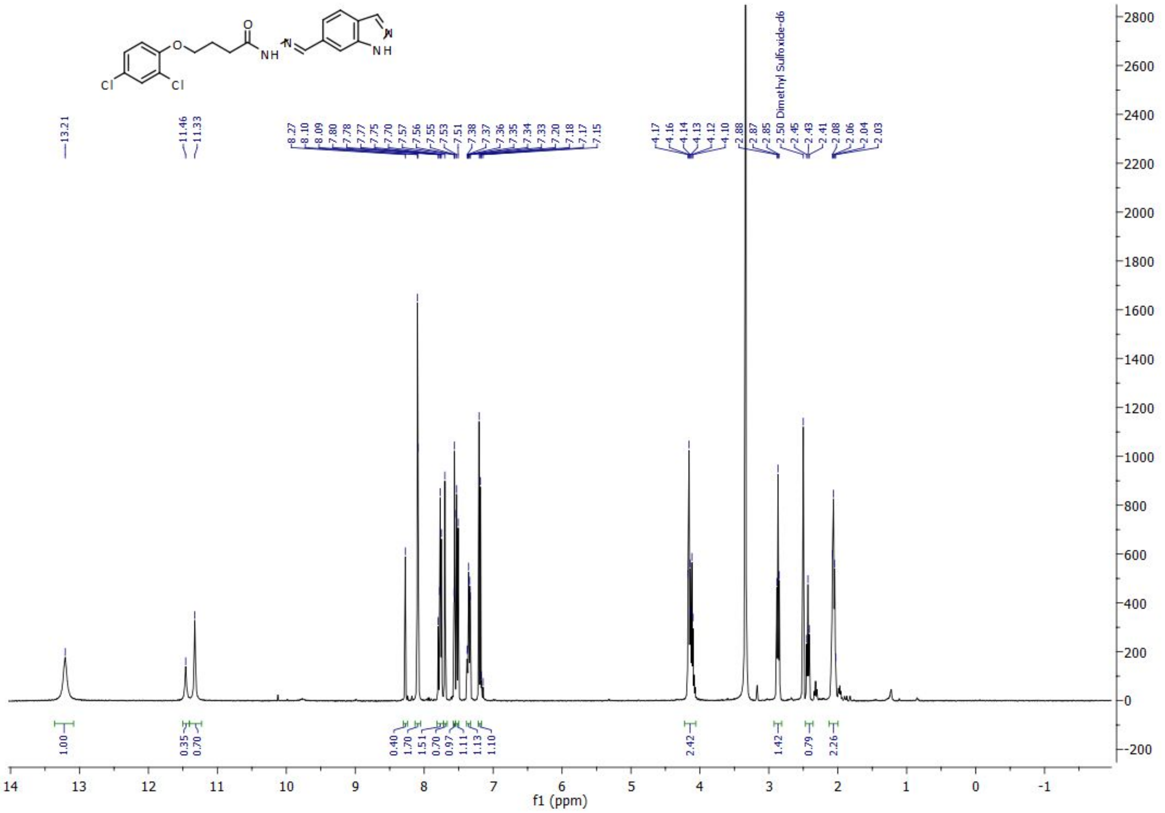

Figure S86. ^{13}C-NMR spectrum (101 MHz, DMSO-d_6) of **15**

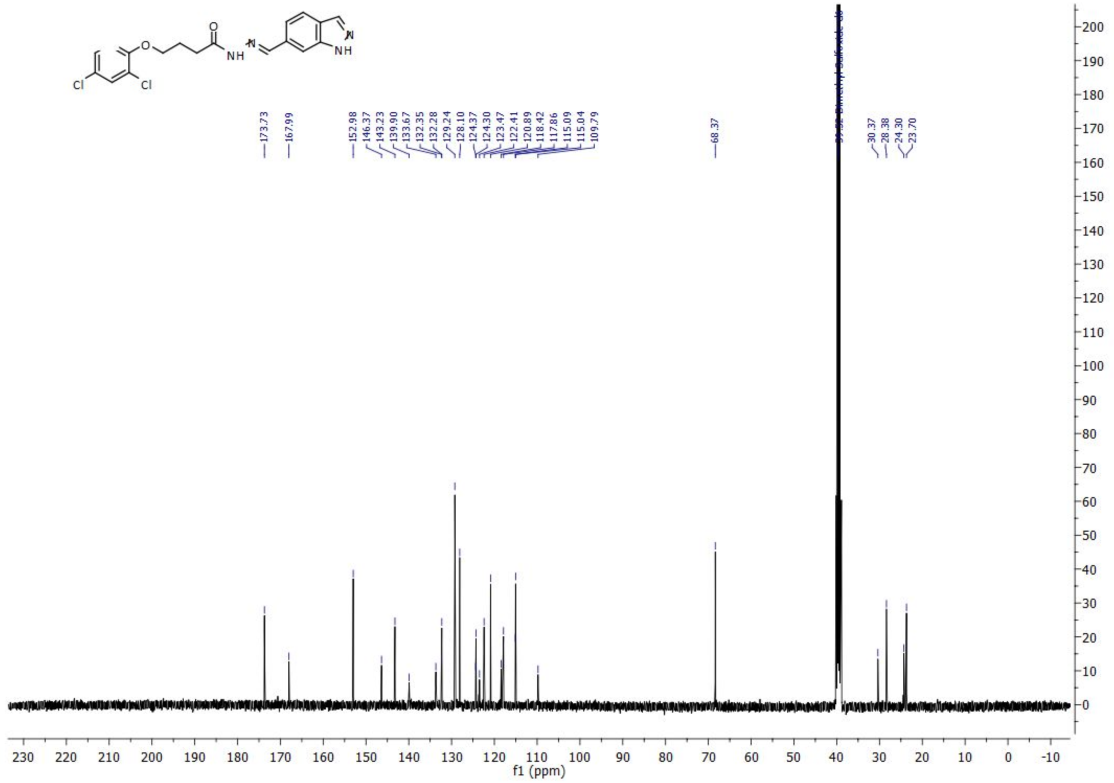

Figure S87. ^{1}H-^{1}H COSY-NMR spectrum (400 MHz, DMSO-d_6) of **15**

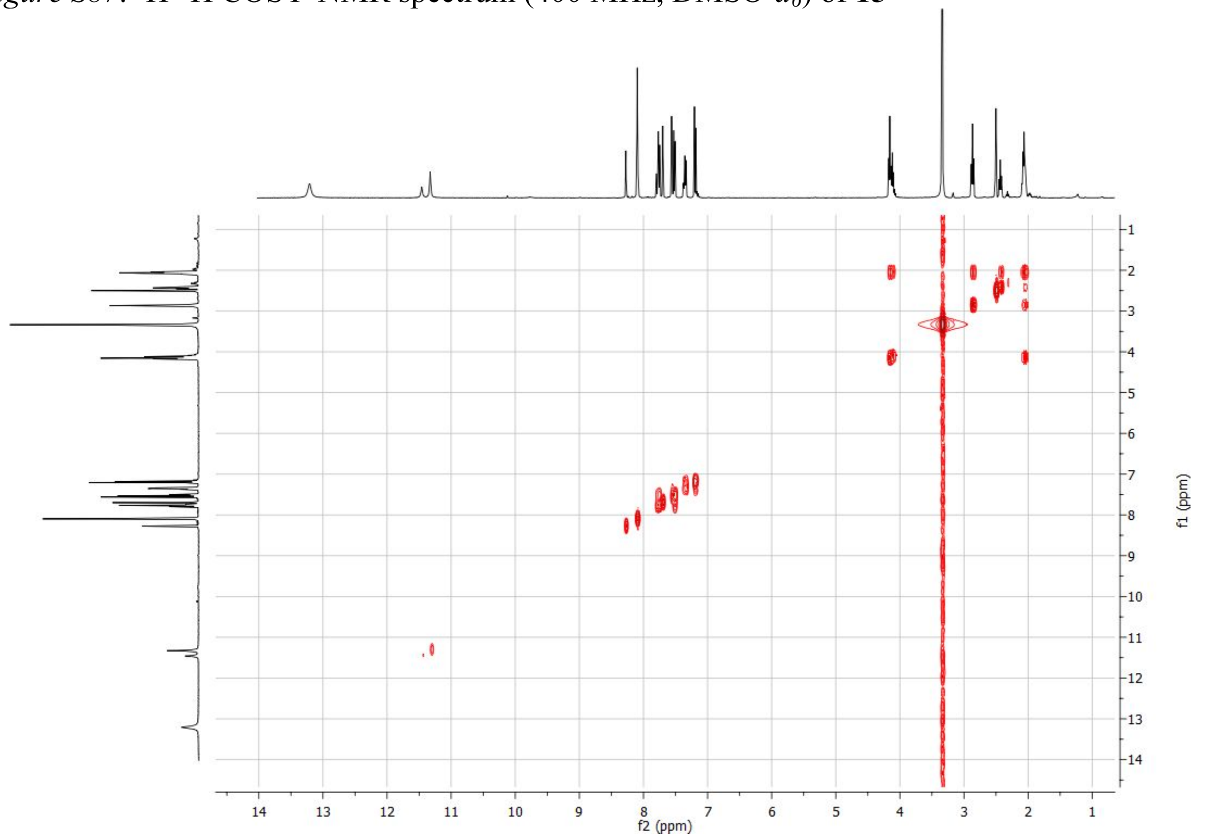

Figure S88. ^{1}H-^{13}C HSQC-NMR spectrum (400 MHz, DMSO-d_6) of **15**

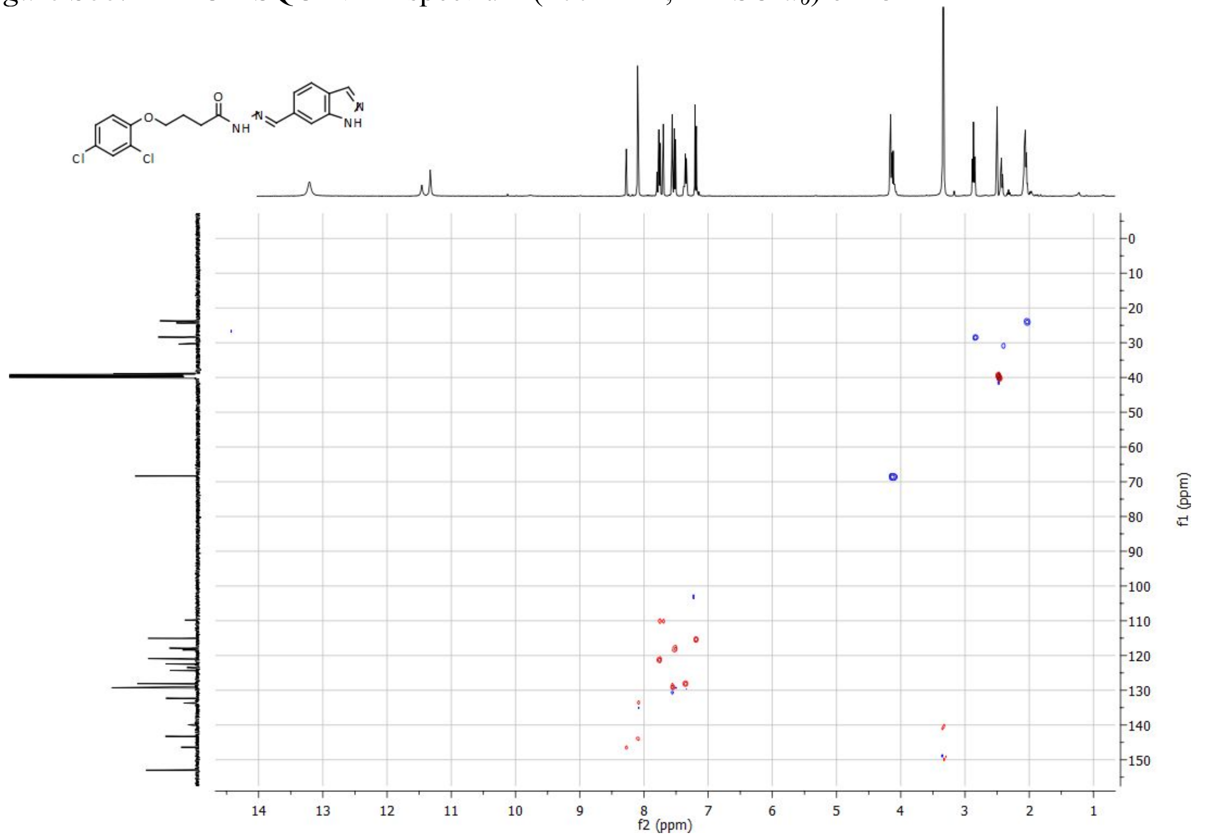

Figure S89. UPLC-MS analysis of **15**

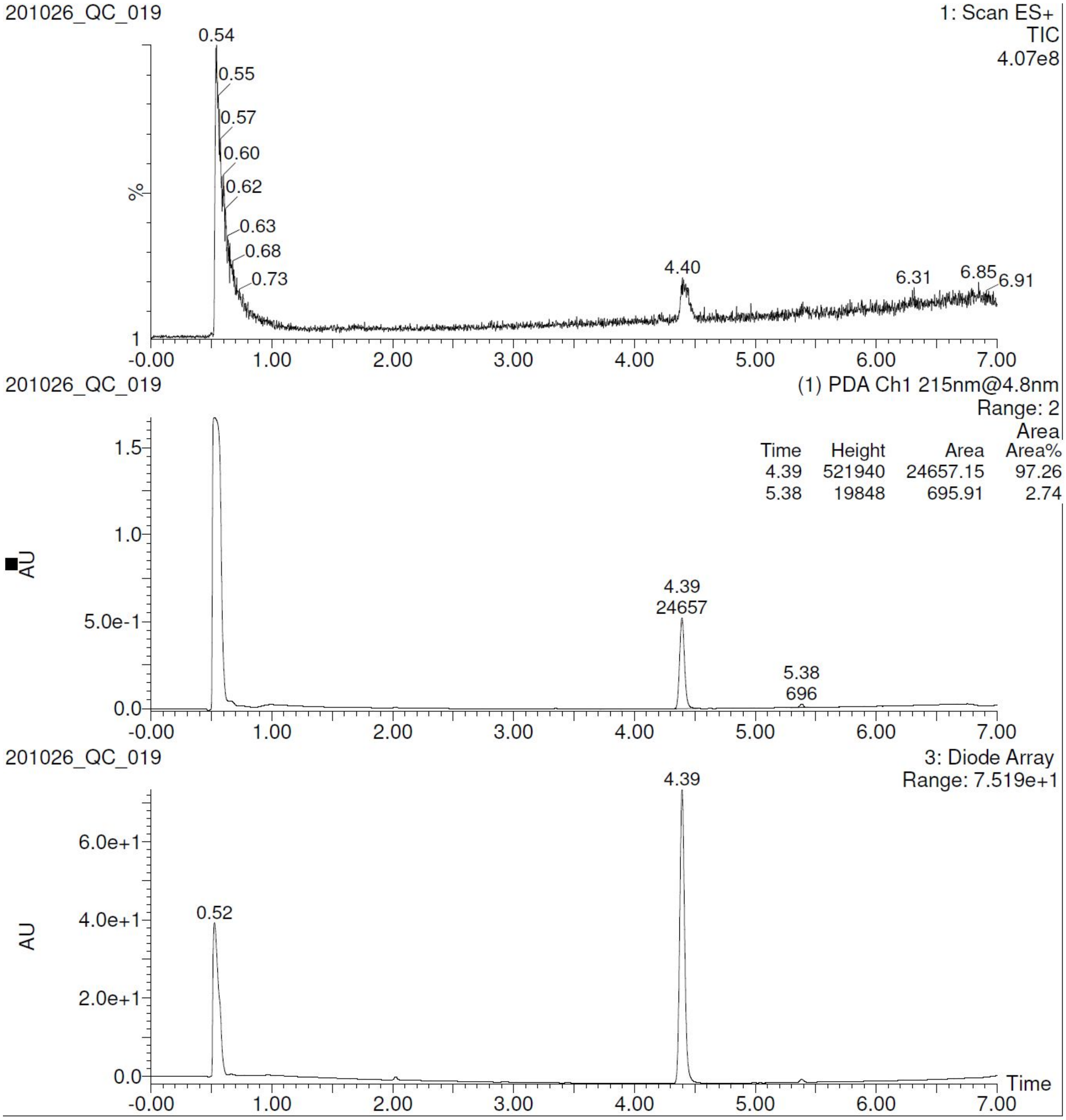

Figure S90. ^{1}H-NMR spectrum (400 MHz, DMSO-d_6) of **16**

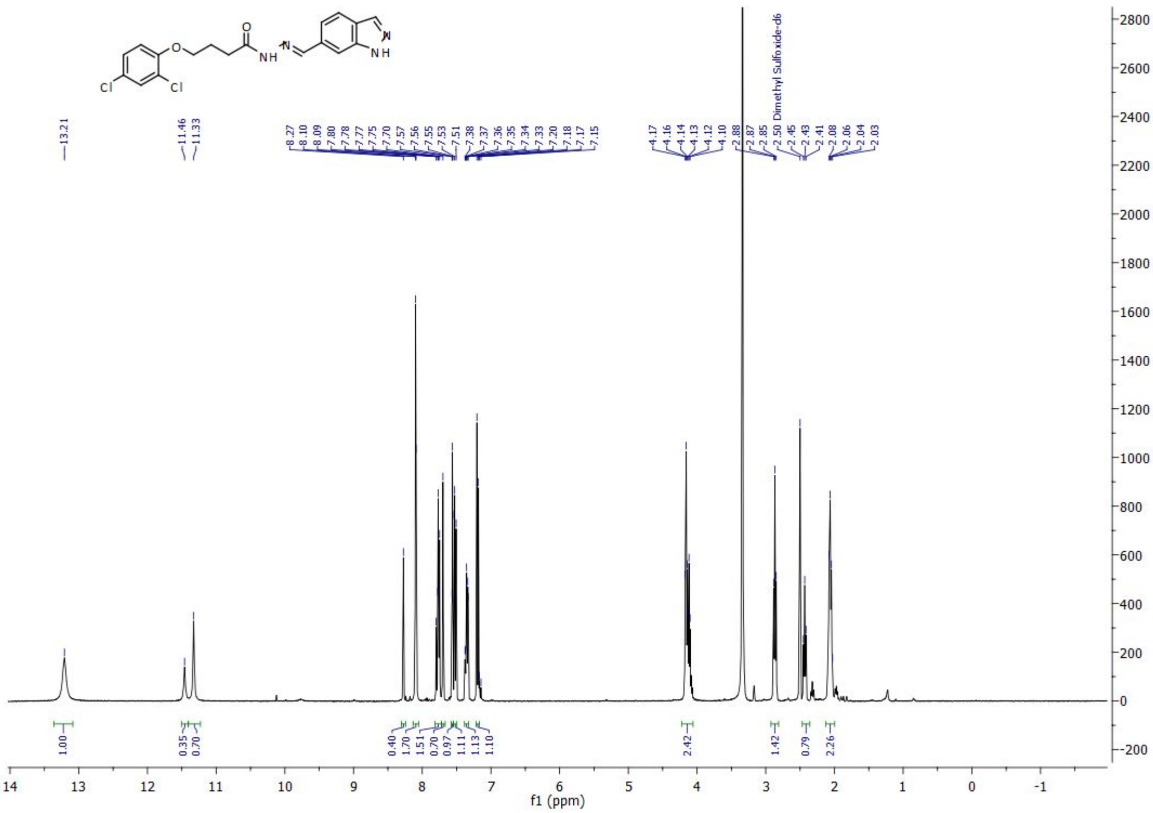

Figure S91. ^{13}C-NMR spectrum (101 MHz, DMSO-d_6) of **16**

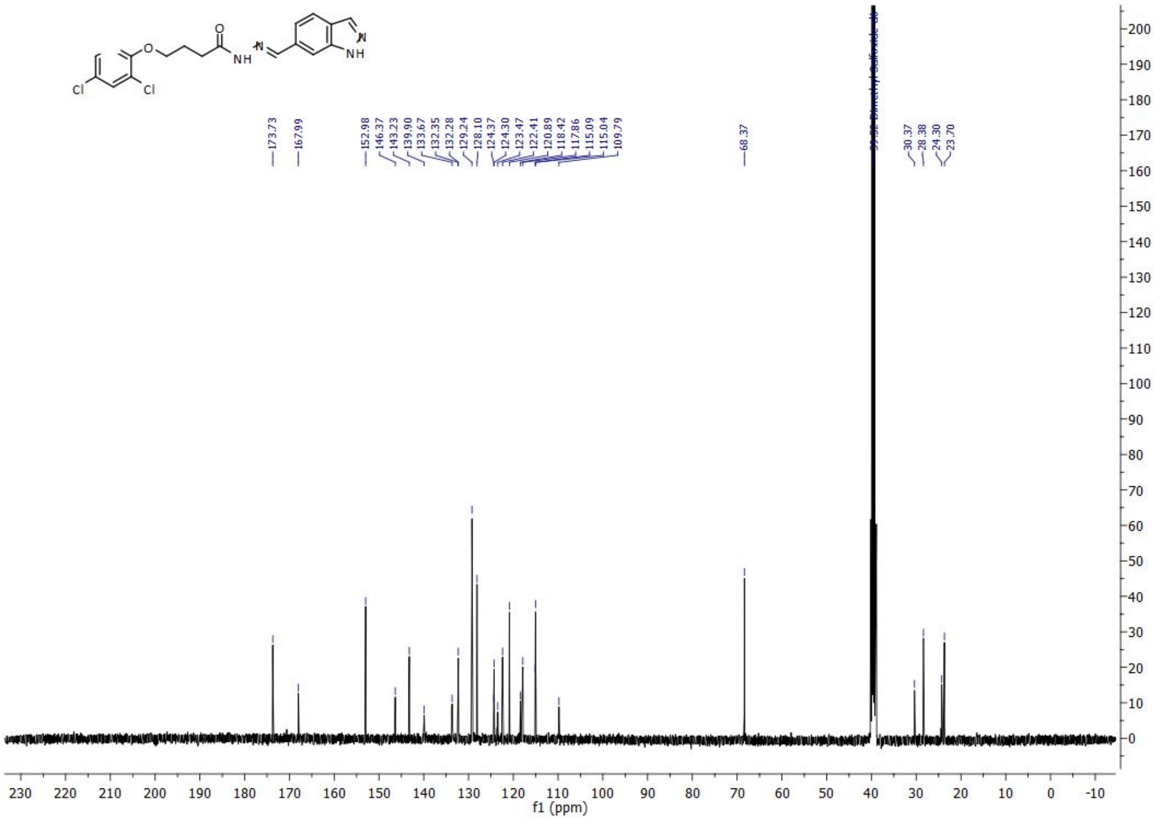

Figure S92. ^{1}H-^{1}H COSY-NMR spectrum (400 MHz, DMSO-d_6) of **16**

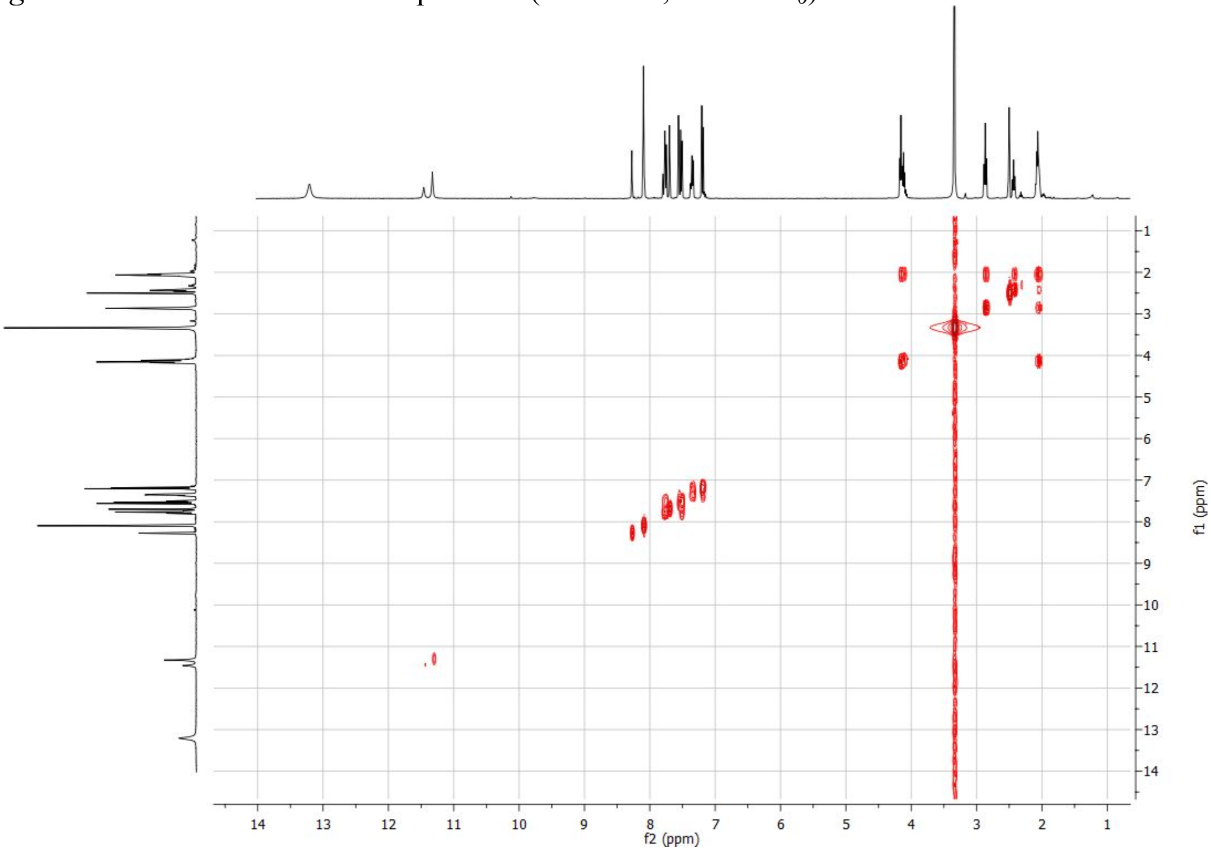

Figure S93. ^{1}H-^{13}C HSQC-NMR spectrum (400 MHz, DMSO-d_6) of **16**

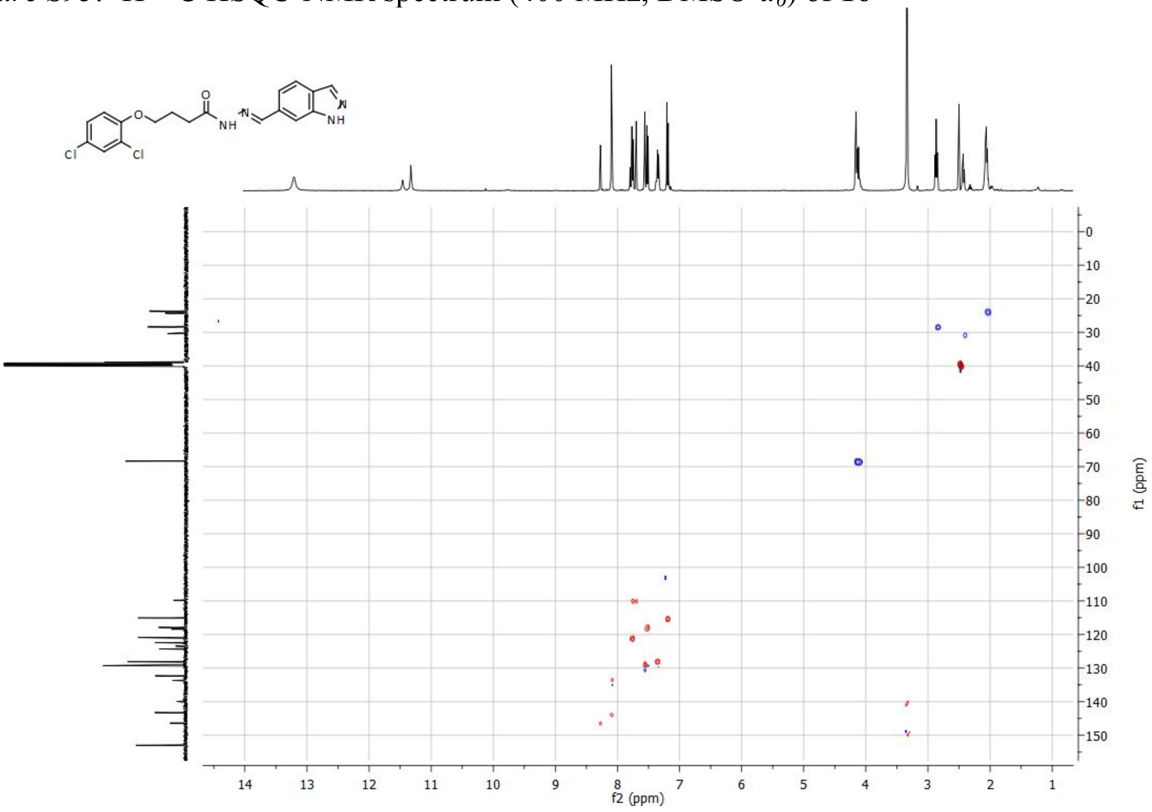

Figure S94. UPLC-MS analysis of **16**

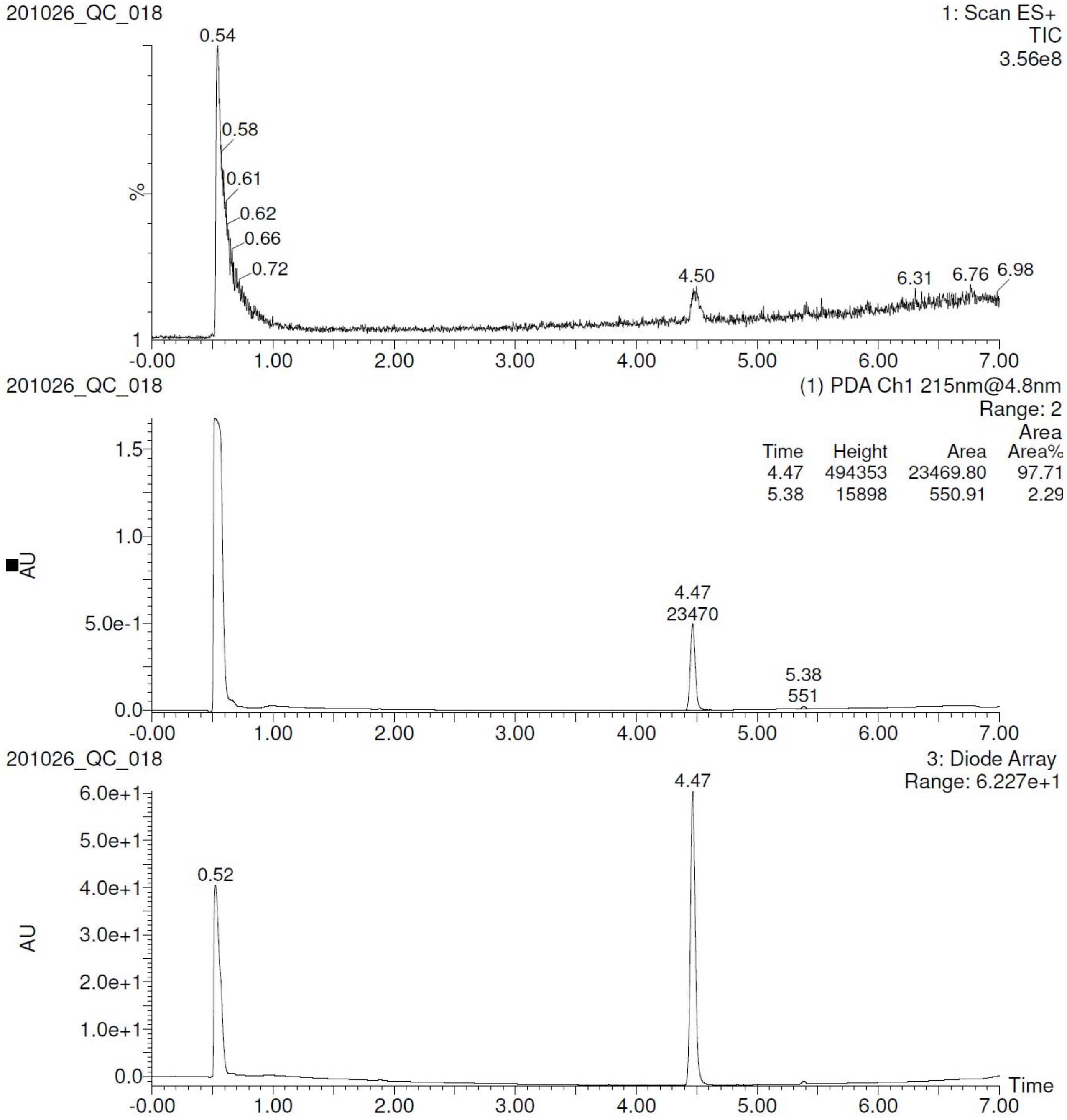

Characterization of *E*/*Z* isomers of *N*-acylhydrazone 7 *via* 2D-NOESY experiment

Figure S95. The 2D-NOESY NMR revealed that the compound **7** was present in as a mixture of *E*,*Z* amide isomers of *E* imine.[5]

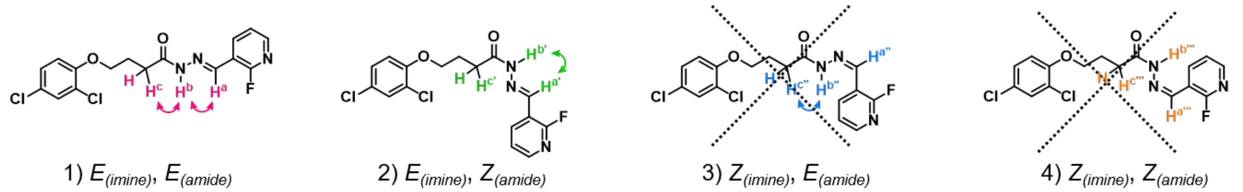

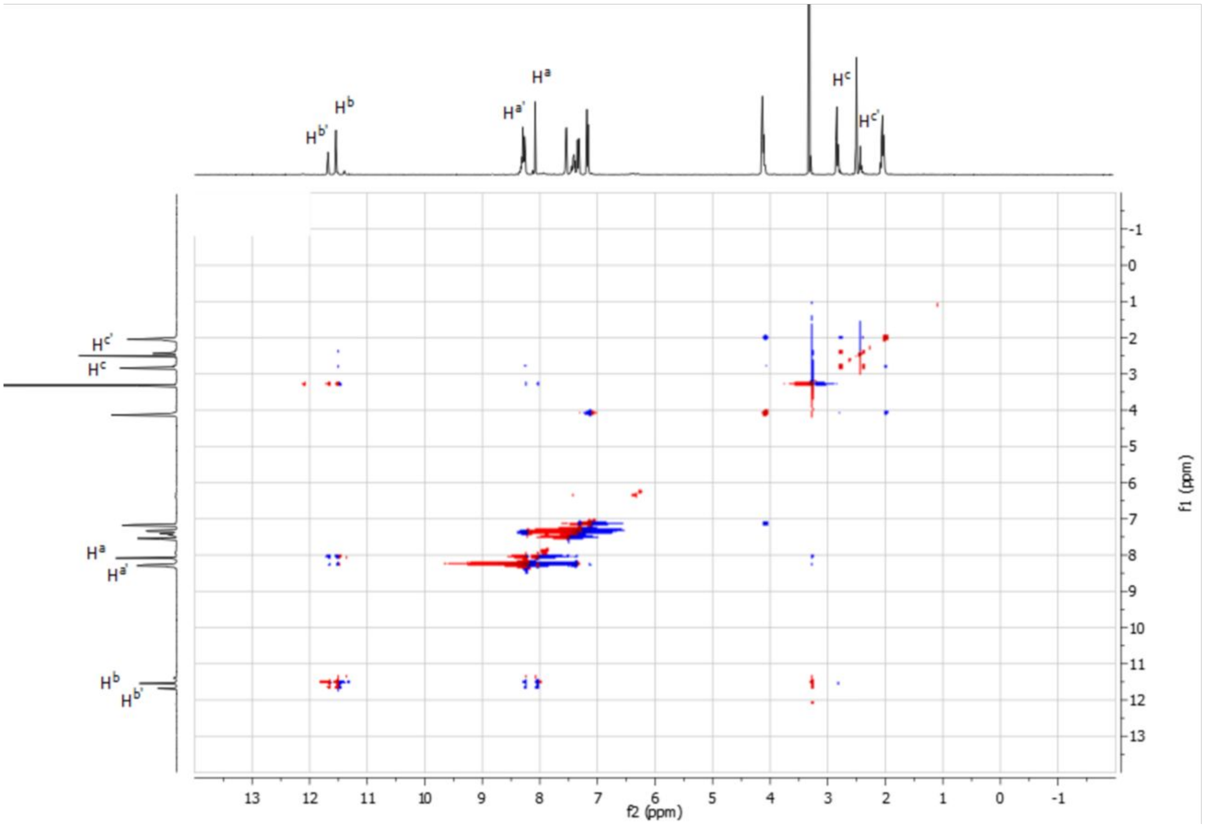

4. Biochemical ELISA assay: dose-response curves of compounds 1─16

Biochemical ELISA assay was performed according to the procedure previously described.[6]

Figure S96. Full dose-response curves for compound **1─16**.

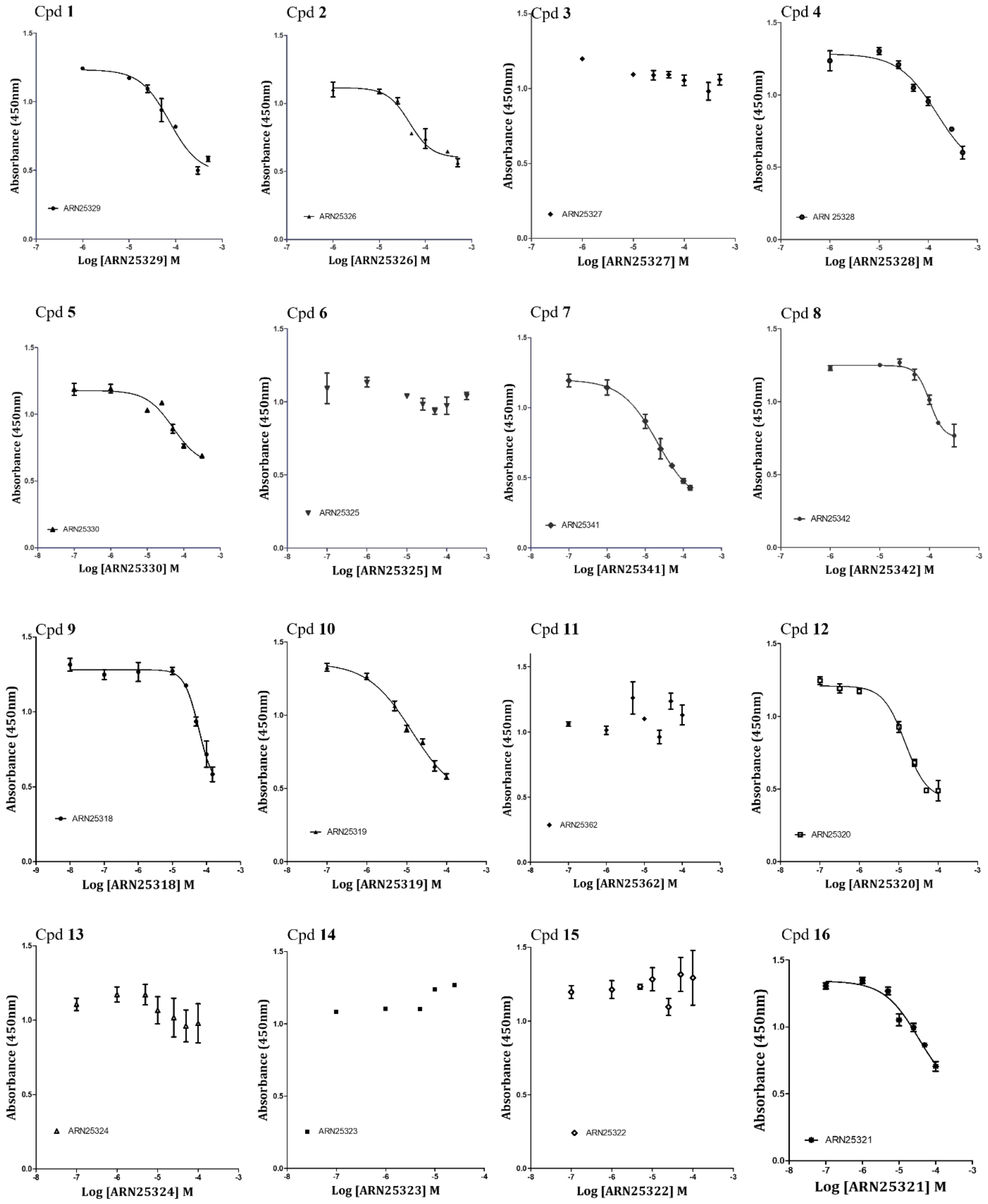

5. ^{19}F-NMR binding assay protocol

Materials and Methods

Recombinant human RAD51 was expressed and purified as previously described.[1]

NMR experiments

All NMR spectra were recorded at 298 K with a Bruker FT NMR AvanceNEO 600 MHz spectrometer, equipped with a 5 mm CryoProbe™ QCI ^{1}H/^{19}F–^{13}C/^{15}N–D with an automatic sample changer SampleJet™ with temperature control. 30 µM of compound 7 was tested by ^{19}F NMR in absence and in presence of 1 µM of RAD51 and 5 µM of BCR4 in 20 mM Hepes pH 8, 250 mM KCl, 0.1 mM EDTA, 2 mM DTT, 1% glycerol, 10% D_2O (for lock signal). For each sample ^{19}F T2-filter experiments were recorded with the Carr-Purcell-Meiboom-Gill scheme[39,40] with a time interval of 20 ms between the 180° pulses and different mixing times (0, 80 and 160 ms respectively). All the NMR experiments were run with proton decoupling using the Waltz 64 composite pulse sequence with a 90° pulse of 120 µs, with a spectral width of 22 ppm, an acquisition time of 1.31 s, a relaxation delay of 5 s and a number of scan of 256. The spectra were transformed using a line broadening of 1 Hz before the Fourier transformation. All the fluorine chemical shift were referred to the $CFCl_3$ signal in water.

6. Computational methods

Docking simulations on Site I and Site II were performed employing the Schrödinger Suite 2020-1. The protein structure model has been prepared with The Protein Preparation Wizard tool[7] starting from the X-ray crystal structure of a RAD51-BRCA2 BRC repeat complex (Protein Data Bank accession code 1N0W). After the removal of all water molecules and ions, all selenomethionines were mutated to methionine prior to add hydrogen atoms and to evaluate and optimize the overall hydrogen bonding network. Then, in order to remove any eventual steric clash, the protein structure was refined with a restrained minimization using the OPLS3 force field.[8] Ligand molecules have been prepared from their 2D structure using the LigPrep utility with the purpose of generating a suitable 3D conformation for the subsequent simulations. Their protonation and tautomeric states at a target pH of 7±1 were evaluated with the Epik software[9] and all the possible rotamers were generated. Docking calculation were run independently on two generated grids for the specific sites (centering on Phe1524 for Site I and Phe1546 for Site II) using the Glide software[10] with the Standard Precision procedure and the OPLS3 force field.

Predicted physicochemical properties for 17–19

Physicochemical properties have been evaluated on the structures (prepared as described above) using the QikProp package (Schrödinger, LLC, New York, NY, 2020).

Table S16. Predicted PhysChem properties for **17─19**.

Compound	**17**	**18**	**19**
#stars	0	2	2
#amine	0	0	0
#amidine	0	0	0
#acid	0	0	0
#amide	0	0	0
#rotor	4	8	8
#rtvFG	0	0	0
CNS	-1	-2	-1
mol_MW	357.444	440.516	424.301
dipole	6.129	4.704	4.583
SASA	654.187	826.151	750.292
FOSA	213.461	265.254	130.787
FISA	119.549	137.091	118.763
PISA	236.537	382.193	317.101
WPSA	84.64	41.613	183.642

volume	1107.419	1443.542	1263.438
donorHB	1	1.25	1
accptHB	4	5.75	4.75
dip^2/V	0.033918	0.015328	0.016623
ACxDN^.5/SA	0.006115	0.007782	0.006331
glob	0.791294	0.747697	0.753304
QPpolrz	37.855	50.13	42.3
QPlogPC16	11.825	16.223	14.765
QPlogPoct	16.725	21.923	18.793
QPlogPw	8.268	10.984	8.932
QPlogPo/w	4.316	5.54	5.455
QPlogS	-6.447	-8.017	-7.677
CIQPlogS	-5.757	-6.821	-6.908
QPlogHERG	-6.037	-7.437	-7.036
QPPCaco	728.137	496.437	740.754
QPlogBB	-0.743	-1.451	-0.863
QPPMDCK	1021.117	392.255	3626.379
QPlogKp	-2.506	-1.932	-1.823
IP(eV)	9.353	9.395	9.159
EA(eV)	1.296	1.078	1.168
#metab	4	3	3
QPlogKhsa	0.673	1.037	0.765
HumanOralAbsorption	1	1	1
PercentHumanOralAbsorption	100	94.678	100
SAfluorine	0	0	0
SAamideO	0	0	0
PSA	77.306	117.889	84.872
#NandO	5	8	6
RuleOfFive	0	1	1
#ringatoms	19	16	16
#in34	0	0	0
#in56	19	16	16
#noncon	4	0	0
#nonHatm	24	31	27
RuleOfThree	1	1	1
Jm	0	0	0

Docking studies: compound 7 and BRC4 overlay

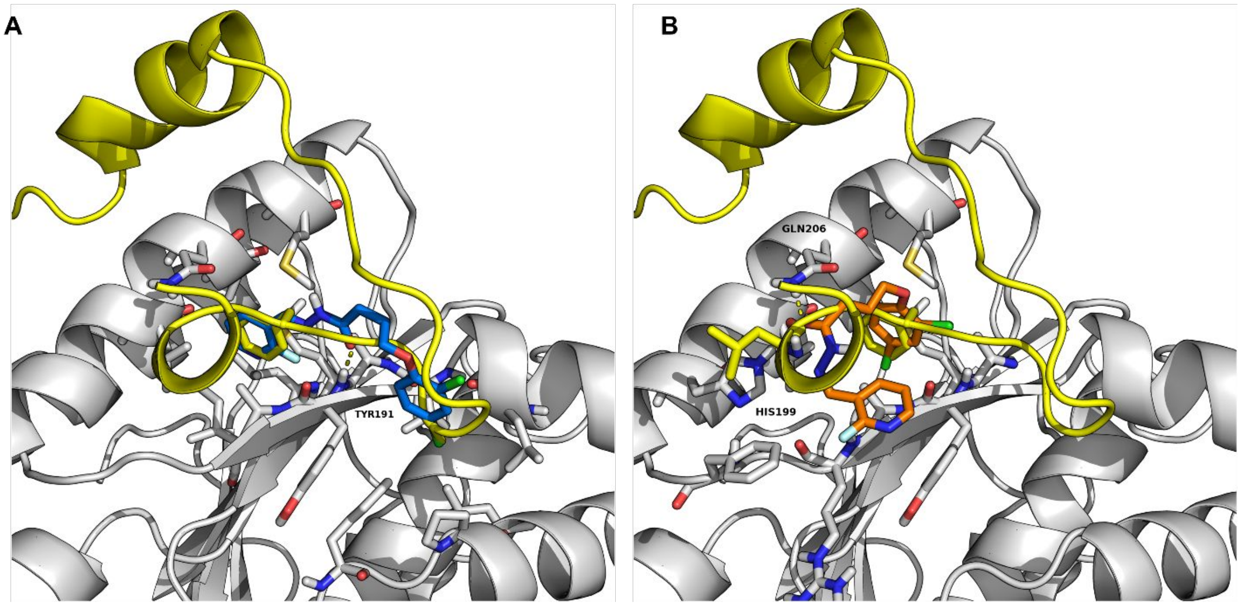

Figure S97. Binding mode of compound 7 in site I as predicted by docking simulations, with BRC4 (yellow cartoon) superimposed to the ligand. RAD51 is depicted as a gray cartoon and rotamer E (A) as blue sticks and Z (B) as orange sticks.

Supplementary References

[1] Bagnolini, G.; Milano, D.; Manerba, M.; Schipani, F.; Ortega, J.A.; Gioia, D.; Falchi, F.; Balboni, A.; Farabegoli F.; De Franco, F.; Robertson, J.; Pellicciari, R.; Pallavicini, I.; Peri S.; Minucci, S.; Girotto, S.; Di Stefano, G.; Roberti, M.; Cavalli, A. Synthetic Lethality in Pancreatic Cancer: Discovery of a New RAD51-BRCA2 Small Molecule Disruptor That Inhibits Homologous Recombination and Synergizes with Olaparib. J. *Med. Chem*. 63, 5, 2588–2619 (**2020**). doi 10.1021/acs.jmedchem.9b01526.

[2] Frei, P.; Pang, L.; Silbermann, M.; Eriş, D.; Mühlethaler, T.; Schwardt, O.; Ernst, B.*i* Target-directed Dynamic Combinatorial Chemistry: A Study on Potentials and Pitfalls as Exemplified on a Bacterial Target. *Chem. Eur. J.* 23, 11570-11577 (**2017**). doi 10.1002/chem.201701601

[3] Frei, P.; Hevey, R.; Ernst, B. Dynamic Combinatorial Chemistry: A New Methodology Comes of Age. *Chem. Eur. J.* 25, 60-73 (**2019**). doi 10.1002/chem.201803365

[4] Jumde, R.P.; Guardigni, M.; Gierse, R.M.; Alhayek, A.; Zhu, D.; Hamid, Z.; Johannsen, S.; Elgaher, A. M.W.; Neusens, P.J.; Nehls, C.; Haupenthal, J.; Reilingfg, N.; Hirsch, A. K. H. Hit-optimization using target-directed dynamic combinatorial chemistry: development of inhibitors of the anti-infective target 1-deoxy-d-xylulose-5-phosphate synthase. *Chem Sci* **2021***, 12*, 7775-7785.

[5] Van der, R.; Guo, H.; Hapko, U.; Eleftheriadis, N.; Monjas, L.; Dekker, F.J.; Hirsch, A.K.H. A combinatorial approach for the discovery of drug-like inhibitors of 15-lipoxygenase-1, Eur. J. Med. Chem. 174, 45–55 (**2019**) doi 10.1016/j.ejmech.2019.04.021

[6] Rajendra, E.; Venkitaraman, A. R. Two modules in the BRC repeats of BRCA2 mediate structural and functional interactions with the RAD51 recombinase. *Nucleic Acids Res* **2010***, 38*, 82-96.

[7] Sastry, G.M.; Adzhigirey, M.; Day, T.; Annabhimoju, R.; Sherman, W., "Protein and ligand preparation: Parameters, protocols, and influence on virtual screening enrichments," J. Comput. Aid. Mol. Des., 2013, 27(3), 221-234

[8] Harder, E.; Damm, W.; Maple, J.; Wu, C.; Reboul, M.; Xiang, J.Y.; Wang, L.; Lupyan, D.; Dahlgren, M.K.; Knight, J.L.; Kaus, J.W.; Cerutti, D.S.; Krilov, G.; Jorgensen, W.L.; Abel, R.; Friesner, R.A., "OPLS3: A Force Field Providing Broad Coverage of Drug-like Small Molecules and Proteins," J. Chem. Theory Comput., 2015, DOI: 10.1021/acs.jctc.5b00864

[9] Greenwood, J. R.; Calkins, D.; Sullivan, A. P.; Shelley, J. C., "Towards the comprehensive, rapid, and accurate prediction of the favorable tautomeric states of drug-like molecules in aqueous solution," J. Comput. Aided Mol. Des., 2010, 24, 591-604

[10] Friesner, R. A.; Banks, J. L.; Murphy, R. B.; Halgren, T. A.; Klicic, J. J.; Mainz, D. T.; Repasky, M. P.; Knoll, E. H.; Shaw, D. E.; Shelley, M.; Perry, J. K.; Francis, P.; Shenkin, P. S., "Glide: A New Approach for Rapid, Accurate Docking and Scoring. 1. Method and Assessment of Docking Accuracy," J. Med. Chem., 2004, 47, 1739–1749

Abbreviations

BBs	Building Blocks
DCC	Dynamic Combinatorial Chemistry
DCL	Dynamic Combinatorial Library
DMSO	Dimethyl Sulfoxide
Exp	Experiment
GP	General Procedure
NAH	N-Acylhydrazone
ptDCC	protein-directed Dynamic Combinatorial Chemistry
RPA	Relative Peak Area
TSA/DSF	Thermal-Shift Assay/Differential Scanning Fluorimetry
